# The energetic cost of human standing balance and gait initiation over a range of natural postures

**DOI:** 10.1371/journal.pcbi.1013522

**Published:** 2026-07-07

**Authors:** Matto Leeuwis, Nikki van Aerts, Ajay Seth, Patrick A. Forbes

**Affiliations:** 1 Department of Neuroscience, Erasmus MC, University Medical Center Rotterdam, Rotterdam, The Netherlands; 2 Department of Biomechanical Engineering, Delft University of Technology, Delft, The Netherlands; Northeastern University, UNITED STATES OF AMERICA

## Abstract

Human movement control is shaped by competing objectives, among which minimizing energy expenditure plays a central role, particularly in determining preferred walking patterns. Whether energetic cost similarly influences standing balance remains unclear because it has not been systematically quantified across a range of natural postures. Importantly, standing is the resting state from which most walking begins, suggesting that the optimization of posture may also reflect the energetic demands of initiating gait. In this study, we use a combination of indirect calorimetry and musculoskeletal simulations to characterize the energetic cost of standing and gait initiation across natural standing postures and investigate whether humans optimize energy expenditure under these conditions. In Experiment 1 (*N* = 13), we measured metabolic cost at preferred and six different prescribed whole-body lean angles. Energy expenditure was lowest at a slight anterior lean (1.15°) and increased monotonically with whole-body lean angle in either direction, rising twice as fast posteriorly compared to anteriorly. This asymmetry challenges the common modeling simplification that effort is symmetric and linear or quadratic with lean angle. Furthermore, participants preferred body angles (1.50 ± 0.73°) with similar energy expenditure to the minimum-cost lean but with significantly more postural variability, suggesting that strict postural regulation was not necessary for minimizing energetic cost. In Experiment 2 (*N* = 20), participants initiated forward and backward walking from preferred or prescribed lean angles. Participants did not alter their standing posture before expected gait initiations in the forward or backward direction, consistent with musculoskeletal simulations showing that leaning further in the anticipated direction did not significantly improve gait initiation time or energetic costs. Together, these findings suggest that postural strategies optimize energy efficiency when permitted by the demands of movement readiness. Our study quantifies the energetic cost landscape that governs human postural control, challenges widely used symmetric estimations of this cost, and offers an empirical foundation for developing more accurate simulations of posture and energy expenditure.

## Author summary

In this study, we investigated whether energy expenditure helps explain how humans select their standing posture. We quantified the energetic cost of standing at various lean angles using indirect calorimetry and musculoskeletal simulations, and found that participants stood close to the lowest-cost angle. Leaning backward incurred an additional energetic cost at twice the rate of forward-leaning postures, which was captured by musculoskeletal simulations but is often omitted in balance control models. Because standing is our idle state from which locomotion is initiated, we also considered how posture changes the energetic cost of walking initiation, and whether expecting to walk forward or backward alters this preference. Interestingly, participants did not change their posture in preparation for the known walking direction, consistent with simulations showing that the energetic benefit of doing so was small. These findings show that energetic cost helps explain preferred human posture during standing and prior to gait initiation, but also reveal that commonly used symmetric assumptions about control effort in inverted pendulum models are inaccurate. Our results provide an experimental basis for the cost landscape that can improve simulations of human standing balance and gait initiation.

## Introduction

Human movement control must balance a variety of competing objectives, such as speed, accuracy, and energy expenditure. During walking, for example, humans tend to use gait patterns (e.g., cadence and speed) that minimize energetic cost per distance traveled [1–11], but trade efficiency for other objectives when the goal demands it (e.g., speed during competition). These tradeoffs can be identified because the energetic landscape for gait is well characterized using simulations [4,12–15] and indirect calorimetry measurements [1,5,6,11], making it possible to determine energy-optimal gait patterns [13–15], simulate new ones [6,10], and even further optimize them with assistive devices [5,16]. Given the evidence of energy optimization in locomotion, it is interesting to consider whether humans also minimize energetic cost while standing. Models of quiet standing often represent postural control as a single- or multi-link inverted pendulum actuated by joint moments [17–30]. In these simplified representations, the moments required to maintain a static orientation scale linearly with the ankle angle and are minimal when standing straight upright. Accordingly, models of postural control are commonly formulated as linear controllers (e.g., proportional-derivative control or linear quadratic regulator) that use the effort (i.e., joint moment; [21,24,28,30]) and/or deviations from a single reference angle or posture [21–27,30] as cost functions. As a result, most models of posture implicitly assume that the cost function representing effort in standing can be characterized as a linear or quadratic function of lean angle, with a single optimal posture in the center.

The actual metabolic cost of standing balance, however, may not follow this relationship. Human anatomy and physiology are more suited for leaning in the anterior direction, with a long forefoot and plantarflexor muscles that enable efficient tonic load bearing [31,32]. Furthermore, numerous joints must be stabilized simultaneously [26], with muscles spanning multiple joints and having different functions and metabolic properties [31–33]. As a result, energy expenditure may not change linearly with standing angle and is likely asymmetric in the anterior and posterior directions. Although energy expenditure has been measured extensively in preferred posture with and without vision [34–40], and in challenging postural conditions that increase variability [38–40], there is limited direct experimental evidence characterizing the metabolic cost across a range of natural standing postures. Without this characterization, it is not possible to determine whether humans minimize energy expenditure during quiet standing in a similar way they do during gait [1–10]. Furthermore, the common modeling simplification that the cost function for effort in posture can be described as symmetric and directly proportional to lean angle [21–27] remains unverified.

An important second consideration for optimizing energy expenditure is that, in daily life, static postures often serve as idle states between brief periods of walking. The most common duration between bouts of walking is ~ 10 seconds, and 60% of walking intervals last less than 30 seconds [41]. Given the abundance of walking transitions from quiet standing, this raises the possibility that the optimization of postural control should also include the energetic cost or time required to initiate movement from that position. For example, athletes begin their sprint from a forward-leaning crouched position, as this enables maximum acceleration [42,43]. However, this posture likely demands more energy and is unsuitable as a resting state. Conversely, sitting expends less energy than standing [34] but is costly for initiating gait. Optimal control in quiet bipedal standing could represent a compromise between the cost of static standing and the cost of gait initiation from that posture, under the constraint of movement onset time. Previous work has shown that kinematic and temporal features of forward gait initiation remain relatively stable across whole-body lean angles [44]. However, it remains unknown whether these different postures differ in energetic costs for gait initiation, or how these costs might affect backward gait initiation. We hypothesize that postural control during quiet standing reflects the optimization of total metabolic cost that includes both the energetic demands of static balance and those of initiating movement in the expected direction. We further expect this function to be shaped by physiological asymmetries in the human body, resulting in a cost that may not be symmetric or proportional to lean angle.

In this study, we examined (i) how metabolic cost varies with standing posture, (ii) how posture affects the energetic cost and time required to complete gait initiation, and (iii) whether humans optimize posture to minimize these costs during quiet standing and in preparation for walking. In Experiment 1 (*N* = 13), we measured kinematics, metabolic cost, and muscle activity as participants stood at a preferred posture and six prescribed sagittal-plane postures, and simulated energy expenditure using a musculoskeletal model. In Experiment 2 (*N* = 20), we used our validated musculoskeletal model to estimate the metabolic cost of initiating gait from three prescribed postures or each participant’s preferred posture in both forward and backward directions. Our results suggest that human postural control approximates optimal energy expenditure of balance and gait initiation by operating near, but not strictly at, a slight anterior minimum-cost whole-body lean angle.

## Results

### Energetic cost landscape of human standing balance

**Fig 1:**
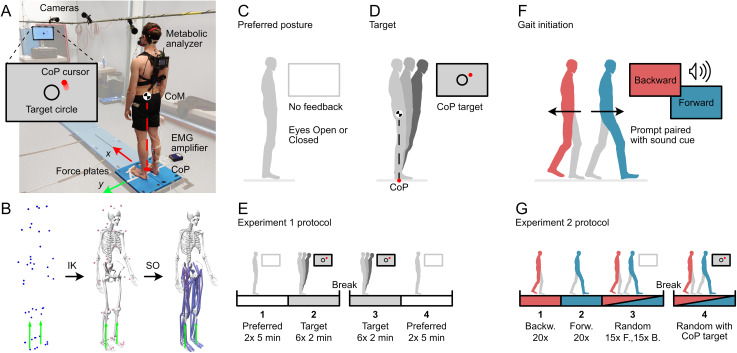
Overview of Experiments 1 and 2. **(A)** The setup of Experiment 1. Participants stood on two force plates and had a total of 43 motion capture markers on their bodies to measure their three-dimensional movement. The back-worn metabolic analyzer measured the flow and air composition at the facemask worn by the participant. The EMG amplifier recorded EMG signals of 8 muscles on the right leg (medial gastrocnemius, soleus, tibialis anterior, vastus medialis, rectus femoris, biceps femoris, semitendinosus, and erector spinae). During target trials, the screen presented a target circle and a cursor of the center of pressure (CoP) in real-time. In Experiment 2, a single force plate was used, and energy expenditure was only estimated from our musculoskeletal model. **(B)** Workflow to estimate muscle activation and energy expenditure from motion data, depicted with simulations from a representative participant. Motion capture data (first panel, blue dots) were used to scale the musculoskeletal model ([13], https://simtk.org/projects/full_body, available under MIT license) to each participant in OpenSim ([14], https://simtk.org/projects/opensim, available under Apache 2.0 license). Inverse kinematics (IK) were then performed for all trials to find their joint angles (second panel). The ground reaction forces (green arrows) were then applied to calculate forces and activations in all 80 modeled muscles using static optimization (SO, implementation by Uhlrich, Jackson [45], including passive forces) at 20 Hz (third panel). Lastly, the model by Umberger, Gerritsen [32] was used to compute the energy expended by each muscle. **(C)** Preferred posture trial; participants self-selected a posture throughout the trial without any feedback on the screen. **(D)** Target trials; whole-body lean angles were prescribed by asking participants to maintain the CoP cursor in a target, which was estimated as the projection of the CoM on the ground plane at the given angle. **(E)** Protocol for Experiment 1. Participants performed two blocks, both consisting of a preferred (panel C, 5 min eyes open and 5 min eyes closed) and target (panel D, 6 targets for 2 min each) trial set with a ~ 10 min sitting break between each block. The starting set (i.e., preferred vs. target) was random in the first block and reversed in the second. **(F)** Gait initiation trials; participants stood at a preferred posture or at a CoP target, and after 5-10 seconds, the screen depicted a prompt (Forward or Backward) to walk in the corresponding direction. Both prompts were paired with a unique sound cue, and a second tone was played 3.1 s after the first prompt to end the trial. **(G)** Protocol for Experiment 2. In the first block, participants stood at a self-selected preferred posture (panel C) and performed three sets of gait initiation trials (panel F) in random order (Forward, Backward, Random). During the second block, participants were presented with CoP targets (panel D) and received a prompt to walk in the forward or backward direction chosen at random.

**Fig 2:**
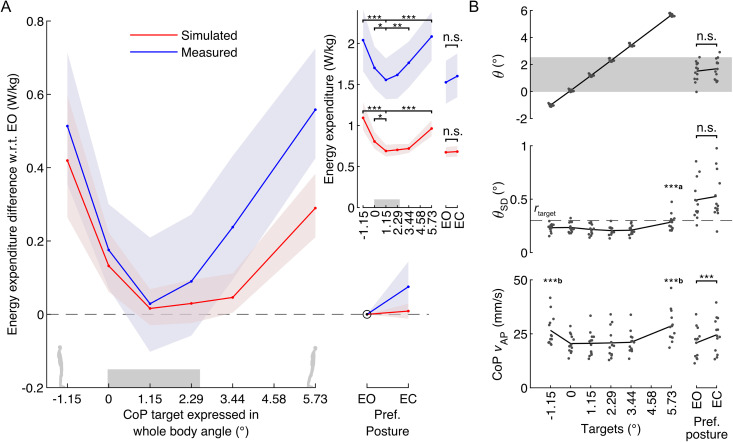
Metabolic energy expenditure of different postures in Experiment 1. **(A)** The means and bootstrapped 95% confidence interval of measured (blue) and simulated (red) metabolic expenditure at each of the target lean angles (-1.15, 0, 1.15, 2.29, 3.44, 5.73°) and the preferred posture with eyes open (EO) and eyes closed (EC). The main panel shows the mass-normalized energy expenditure difference relative to the preferred posture EO condition (subtraction performed for each participant independently). The inset shows the mass-normalized absolute energy expenditure. Note that the simulated expenditure as computed from the musculoskeletal simulation only considers lower-limb skeletal muscles and omits contributions from any other source, whereas indirect calorimetry measurements represent whole-body energy expenditure. **(B)** Participant means for whole-body angle *θ*, within-trial standard deviation of whole-body angle *θ*_SD_, and average magnitude of the CoP velocity in the anteroposterior (AP) direction during target and preferred posture trials. The radius of the target (0.3°) is denoted as *r*_target_. The range between the most posterior and anterior preferred postures observed in the EO condition (-0.03 to 2.54°) is marked in gray in both panels. ^**a**^ Significant difference w.r.t. all other targets, ^**b**^ Significant difference w.r.t. targets in the range of 0 to 3.44°, *: *p* ≤ 0.05, **: *p* ≤ 0.01, ***: *p* ≤ 0.001.

In our first experiment (*N* = 13), we measured participants’ energy expenditure at preferred and prescribed postures using indirect calorimetry to determine how energy expenditure varies over natural postures. During preferred posture trials, participants self-selected a lean angle throughout the trial (eyes open and eyes closed, 2 x 5 min each, Fig 1A, 1C, 1E). Most participants adopted a slight forward lean in both the eyes-open (12/13) and eyes-closed (13/13) conditions (Fig 2B). The range of average preferred postures across participants spanned from -0.03 to 2.54° for eyes-open and 0.70 to 2.91° for eyes-closed standing (see Table 1 and Fig 2B). On average, these whole-body lean angles did not differ significantly between the eyes open and closed conditions (*t*(12) = -2.10, *p* = 0.058). Similarly, there was no significant difference in measured energy expenditure between eyes open and closed standing (see Table 1; Fig 2A inset).

**Table 1:** Energy expenditure (measured and simulated) and balance characteristics for Experiment 1.

	EE Measured			EE Simulated			*θ*	*θ*_SD_	CoP *v*_AP_
	W/kg	*t*(11)	*p*	W/kg	*t*(12)	*p*	°	°	mm/s
Target									
-1.15°	2.04 ± 0.68	5.18^**a**^	**<0.001**	1.09 ± 0.31	5.03^**a**^	**<0.001**	-1.01 ± 0.08	0.23 ± 0.04	26.6 ± 7.0^**e**^
0°	1.70 ± 0.46	2.99^**a**^	**0.012**	0.81 ± 0.19	3.01^**a**^	**0.016**	0.07 ± 0.07	0.24 ± 0.05	20.5 ± 4.3
1.15°	1.56 ± 0.46	–		0.69 ± 0.13	–		1.17 ± 0.07	0.22 ± 0.05	20.6 ± 5.5
2.29°	1.61 ± 0.54	0.65^**a**^	0.265	0.70 ± 0.13	0.68^**a**^	0.318	2.29 ± 0.08	0.21 ± 0.04	20.8 ± 6.8
3.44°	1.76 ± 0.52	3.54^**a**^	**0.007**	0.72 ± 0.10	1.04^**a**^	0.318	3.41 ± 0.08	0.21 ± 0.04	21.1 ± 5.1
5.73°	2.08 ± 0.53	11.50^**a**^	**<0.001**	0.96 ± 0.17	5.45^**a**^	**<0.001**	5.66 ± 0.09	0.29 ± 0.06^**d**^	28.7 ± 7.6^**e**^
Preferred posture									
EO	1.53 ± 0.48	-0.36^**b**^	0.727	0.67 ± 0.11	-0.64^**b**^	0.535	1.50 ± 0.73	0.49 ± 0.18	20.9 ± 6.9
EC	1.60 ± 0.49	2.10^**c**^	0.059	0.68 ± 0.12	0.84^**c**^	0.418	1.68 ± 0.75	0.53 ± 0.22	24.6 ± 8.5

EO: Eyes Open, EC: Eyes Closed, EE: Energy Expenditure. Post-hoc statistics for rmANOVA on *θ*_SD_ and CoP *v*_AP_ are provided in S1 Appendix. Significant differences are highlighted in bold. ^**a**^ one-sided *t*-tests against lowest-cost target (1.15°, Holm-corrected), ^**b**^ *t*-test against target 1.15°, ^**c**^ *t*-test against EO, ^**d**^ Significant difference w.r.t. all other targets, ^**e**^ Significant difference w.r.t. target 0 to 3.44°.

In the target condition trials, six whole-body lean angles were prescribed (-1.15, 0, 1.15, 2.29, 3.44, and 5.73°) using real-time feedback of the center of pressure (CoP) position on a screen (Fig 1A, 1D). The energetic cost measured through indirect calorimetry was minimal at the 1.15° target (anterior) and increased in both lean directions (Fig 2A). For visualization, the offset due to the basal metabolic cost (i.e., the energy expended by non-motor processes to uphold bodily function) and model simplifications (i.e., muscles beyond the lower limbs) was removed by subtracting the cost of the preferred posture trial with eyes open per participant for both the measured and simulated data (Fig 2A; data without this subtraction are shown in the inset). At the minimum-cost target (1.15° anterior), the energy expenditure was not significantly different from the preferred posture trials with eyes open (Table 1, Fig 2A). Across all postures, the energy expenditure varied significantly with the prescribed target (*F*(5, 55) = 13.83, *p*_target_ < 0.001, Table 1, Fig 2A), increasing total energy expenditure by up to ~31% at the most anterior target. To assess how energetic cost increased with respect to the minimum-cost posture, we performed planned post-hoc comparisons of energy expenditure across targets to the minimum-cost target using one-sided *t*-tests. The difference with respect to the minimum-cost target was significant for all posterior postures and for most more anterior postures (Table 1, Fig 2A inset). This pattern of increasing energy expenditure at larger anterior and posterior lean angles was accompanied by a small increase in minute volume (total air circulated per minute) and breath frequency at both outer targets relative to the minimum-cost target (see S1 Appendix for statistics and values). For minute volume, there was no significant difference between the minimum-cost target and the preferred posture (*t*(11) = -0.15, *p* = 0.883), while breathing frequency was ~ 25% higher at the minimum-cost target (*t*(11) = -2.99, *p* = 0.012).

To test whether the increase in metabolic cost was symmetric around the minimum-cost target (1.15°), we calculated the average rate of change in energy expenditure across adjacent targets in both the anterior and posterior directions from this minimum. The average slope in the posterior direction was -0.21 ± 0.14 W/kg/°, whereas the slope in the anterior direction was 0.11 ± 0.05 W/kg/°. The absolute values of these slopes differed significantly (*t*(11) = 3.62, *p* = 0.004), indicating that the energetic cost of anterior-posterior lean was asymmetric, increasing at almost twice the rate in the posterior direction. Overall, these results show that energy expenditure during quiet standing is a function of whole-body lean angle, with a clear energetic minimum near a slight forward lean.

### Preferred postures are variable but centered around the minimum-cost whole-body lean

Although participants generally preferred postures near the energetic minimum, their whole-body lean angle also showed substantial variation both within and across individuals (see Fig 2B). To assess within-trial variability, we analyzed the CoP velocity in the forward-backward direction and the standard deviation of whole-body lean angle. We first compared responses from the preferred posture trials (eyes-open) with the minimum-cost posture (i.e., 1.15°), which was also the target nearest to the preferred posture (Fig 2A). The average magnitude of the CoP velocity, which is an indication of the magnitude of postural corrections, did not differ between the preferred posture and the minimum-cost target posture (*t*(12) = 0.16, *p* = 0.873; Table 1 and Fig 2B). The within-trial standard deviation of lean angle during preferred posture was roughly double that of the minimum-cost target (*t*(12) = 5.77, *p*_Holm_ < 0.001; Table 1 and Fig 2B), likely because the absence of a target allowed the participants’ posture to drift. This indicates that variability in whole-body lean (i.e., standard deviation of lean angle) was greater during preferred standing than at the minimum-cost target, but did not translate into increased postural corrections (CoP velocity) or higher energy expenditure. Overall, these findings suggest that minimizing whole-body variability, a common objective in postural control models, may not be a prerequisite for minimizing energy expenditure during quiet standing.

Next, we compared these same responses across the target trials. Average anteroposterior CoP velocity differed across the targets (*F*(2.62, 31.4) = 19.61, *p*_target_ < 0.001), with pairwise comparisons showing significant increases (~30%) observed only at the most posterior and anterior targets compared to all others (see Fig 2B and S1 Appendix for statistics). Similarly, the standard deviation of the whole-body angle was also significantly different across postures (*F*(2.07, 24.87) = 13.22, *p*_target_ < 0.001); however, pairwise comparisons revealed that only the most anterior target was significantly increased from all others (see Fig 2B and S1 Appendix for statistics). Finally, closing the eyes did not change the within-trial variability of whole-body lean (*t*(12) = -0.75, *p* = 0.466, Fig 2B), but increased the CoP velocity by around 18% (*t*(12) = -4.39, *p* = 0.001, Fig 2B) compared to preferred posture with eyes open.

To identify possible mechanical sources of across-participant variability, we assessed whether individual variability in ankle stiffness could account for differences in preferred posture. When standing is simplified as a single inverted pendulum, the actively generated moment is minimal when the passive moments generated by the tissues around the ankles counteract the gravitational moment acting on the body. We used a published model of ankle stiffness [46] to estimate a distribution of whole-body lean angles where the passive ankle moment is in equilibrium with the gravitational moment of a body with a height equivalent to our group average (see Materials and Methods). Using a Monte Carlo analysis, we found that the simulated equilibrium angles formed a right-skewed normal distribution with a median of 1.25° and an interquartile range of 0.51° [1.02, 1.53°] (data reported in non-parametric metrics to account for skewness of the distribution). For comparison, the preferred postures measured in Experiment 1 had a median of 1.68° and an IQR of 1.14° [0.93, 2.07°]. This suggests that individual ankle mechanics may partly explain between-participant differences in preferred posture though participants generally adopted more forward-leaning postures than predicted by the passive equilibrium angles alone.

### Musculoskeletal model simulations predict the trend of metabolic cost across postures

To better understand the physiological basis of postural energy expenditure, we used a musculoskeletal model in OpenSim [13,14] to estimate muscle-level energetic cost during standing (Fig 1B). Muscle energy expenditure was computed for 80 muscles using a physiological model [32] that accounted for the muscle’s activation, mass, fiber composition, and (tendon) stiffness. Similar to our experimental data, the energy expenditure in our simulations was lowest at 1.15° and varied significantly with the target lean angle (*F*(1.62, 19.4) = 15.63, *p*_target_ < 0.001), increasing at both posterior and anterior leans. Relative to the minimum-cost posture, the simulated cost increased significantly at all posterior postures and at the most anterior posture (Fig 2A, Table 1). Overall, simulations captured the trends in measured energy expenditure, though they underestimated the cost for anterior-leaning postures.

**Fig 3:**
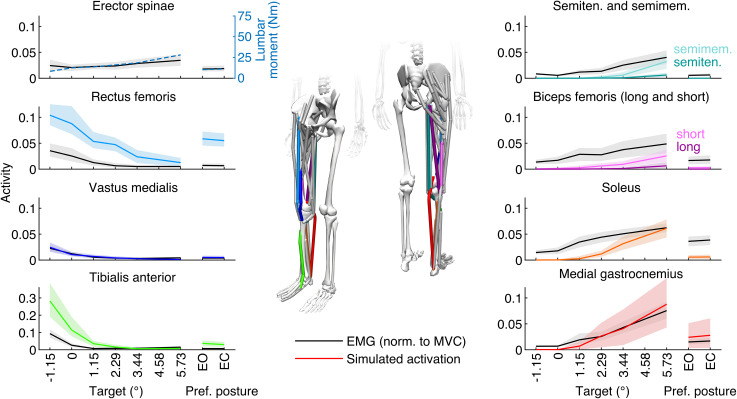
Measured (black) and simulated (colored) activity of major postural muscles on the right side of the body during Experiment 1. Solid lines indicate the mean activity across participants, and the shaded areas represent the bootstrapped 95% confidence interval of the mean. The EMG activity is shown for the rectus femoris, vastus medialis, tibialis anterior, semitendinosus, biceps femoris (long head), soleus, and medial gastrocnemius muscles. The EMG activity of the erector spinae was compared against the trend of the lumbar extension moment (dashed line), as the muscle was not present in the musculoskeletal model [13]. Activations for all muscles included in the model are provided in S1 Fig. The simulated activations of the semimembranosus and semitendinosus are shown in the same panel as they share a similar function. Electromyography data were normalized to the largest value observed during maximal voluntary contraction, and simulated muscle activations were inherently constrained between zero and one. The central model visualization was generated with the unscaled musculoskeletal model ([13], https://simtk.org/projects/full_body, available under MIT license) with the left leg muscles hidden for clarity and muscle colors matched to the activation traces shown in each panel.

To validate the simulated muscle activations and explore posture-dependent muscle recruitment, we compared experimental EMG recordings with simulated activations for eight major postural muscles (Fig 3). Overall, the simulations captured the qualitative trends in EMG across postures, although absolute magnitudes likely differed due to normalization [47]. Muscles responsible for dorsiflexion (tibialis anterior) and knee extension (rectus femoris, vastus medialis) were highly active in backward postures, with activation declining as the body leaned forward. In contrast, forward postures increased reliance on ankle plantarflexors (soleus, gastrocnemius) and hip extensors (semitendinosus, semimembranosus). The gluteal muscle activation in our simulation results also increased with lean angle, but to a lesser extent than the plantarflexors (see S1 Fig for activations of all simulated muscles). As the erector spinae was not included in our simulations, we compared the model’s lumbar extension moment to the experimentally measured muscle activity. Both signals followed a slight upward trend, suggesting that the contributions of trunk muscles gradually increase with greater anterior leaning.

To better understand the different physiological contributions to energy expenditure, we decomposed the total metabolic cost from the simulations into activation maintenance, fiber shortening rate, and mechanical work [32]. The activation maintenance reflects the metabolic losses of sustaining a constant, isometric muscle force, including processes such as calcium cycling and cross-bridge turnover [32]. The shortening rate accounts for additional losses occurring when the muscle fibers change length, while mechanical work represents the muscle’s idealized external work [32]. During preferred posture trials, nearly all energy expenditure was attributed to activation maintenance costs (99.4 ± 0.6%), with only a minimal contribution from the fiber shortening rate (0.6 ± 0.6%) and none from mechanical work. This confirms that static standing primarily expends energy through sustained low-level muscle activation, rather than dynamic contraction or external work.

Interestingly, the proportion of energy expended by muscles consisting mostly of more energy-intensive fast-twitch muscle fibers (type II) also varied with lean angle. At the most posterior target (-1.15°), 70.0 ± 9.4% of the simulated energy expenditure came from muscles with relatively high (> 52%) fast-twitch fibers, such as the rectus femoris and vastus lateralis [31]. In contrast, at the most anterior target (5.73°), only 42.6 ± 13.8% of expended energy came from such muscles, with the remainder of the energy being consumed by muscles consisting primarily of slow-twitch fibers (type I), such as the soleus [31]. This result suggests that posterior leaning may recruit less metabolically efficient muscles for prolonged activation maintenance, contributing to the higher energetic cost observed when leaning in that direction.

### Preferred quiet standing posture is not affected by the metabolic cost of gait initiation

So far, we observed that participants preferred whole-body angles close to the minimum-cost angle (Fig 2). However, the optimization of posture may reflect more than just minimizing the cost of static standing; it may also be shaped by the need to initiate movement quickly, efficiently, and frequently [41,43]. To explore this proposition, Experiment 2 investigated how the energetic cost and time required to initiate gait vary with posture, and whether participants adjust their static posture in anticipation of the movement direction. Participants (*N* = 20) stood on a force plate at a target or at their preferred posture and initiated gait when prompted to start walking in a forward or backward direction (Fig 1F-1G, see Materials and Methods).

**Fig 4:**
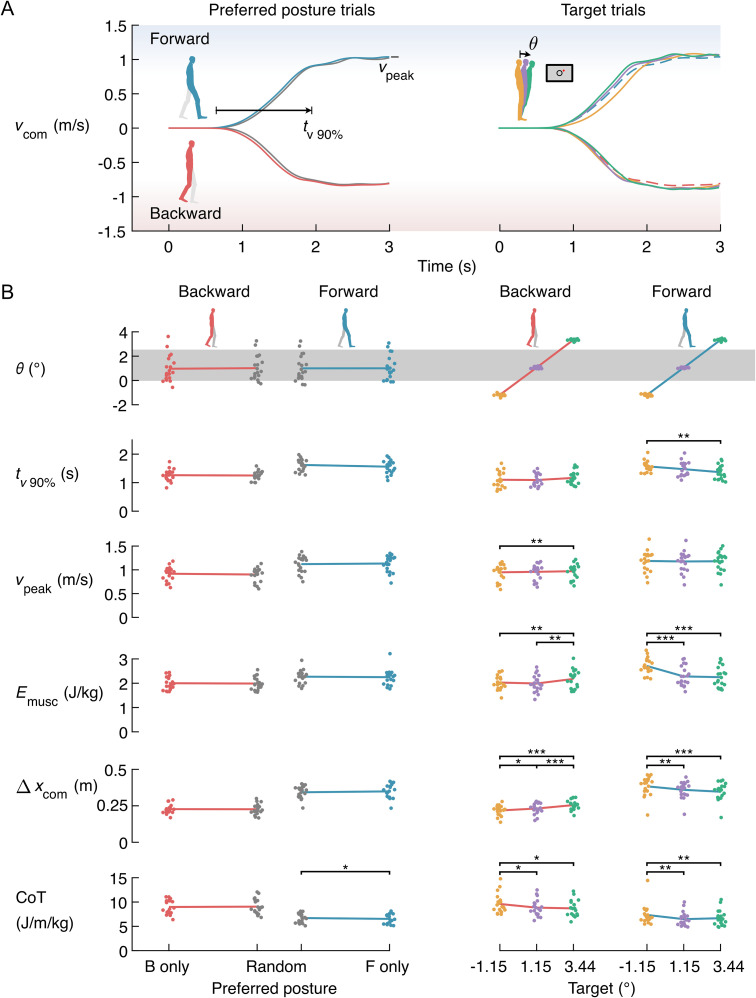
Gait initiation results for preferred posture and target trials of Experiment 2. In preferred posture trials, the participant could self-select a posture during the period before the cue. In target trials, participants were prescribed a lean angle using the CoP target. **(A)** Mean forward velocity of the center of mass (*v*_com_) for all participants (*N* = 20) grouped by trial. Blue and red lines correspond to the Forward- and Backward-only block, respectively, where participants were aware that they would only initiate gait in the corresponding direction (repeated as dashed lines in the right figure for comparison). The gray lines (lagging slightly behind the blue and red lines) show the initiation in the Random direction block, where the direction was unpredictable. The colored lines (yellow, purple, green) correspond to the three prescribed targets (-1.15, 1.15, 3.44°, respectively). **(B)** Results of gait initiation metrics. The gray band in the whole-body lean angle plot indicates the range of preferred postures for prolonged standing as observed in Experiment 1. Forward and backward-only initiation differ significantly for all metrics except *θ* (Table 2)*.* For reference, simulated activations of all muscles are provided in S2-S3 Figs. Variables shown are *θ:* whole-body lean angle, *t*_*v*90%_: time to reach 90% of steady-state velocity with respect to the start of movement, *v*_peak_: peak forward or backward velocity (absolute value), *E*_musc_: simulated muscle energy expenditure between prompt and foot strike, Δ*x*_com_: change in center of mass location over that period, CoT: cost of transport, equal to *E*_musc_/ Δ*x*_com_. *: *p* ≤ 0.05, **: *p* ≤ 0.01, ***: *p* ≤ 0.001.

First, we assessed whether participants adjusted their preferred standing posture in preparation for the upcoming gait initiation direction. Participants were explicitly informed as to which set of trials (Forward only, Backward only, Random) they would have to perform, and could therefore anticipate the probability of the walking direction but not the timing of the walking prompt. The lean angle during standing before gait initiation did not differ significantly between sets of randomized or known walking directions, and was also not different between walking forward or backward only (Fig 4B, Table 2). Similarly, the time required to reach steady-state walking (i.e., when the center of mass velocity reached 90% of their average maximum velocity for that direction) was not significantly different when the direction was randomized or known in either direction (Table 2). Between forward and backward walking with known directions, the time to reach steady state walking was around 19% shorter during backward as compared to forward walking initiation, likely because the maximal absolute velocity in backward walking was also roughly 19% lower (Fig 4B, Table 2).

**Table 2:** Results and statistics for Experiment 2.

	*θ*	*t*_*v* 90%_	*v*_peak_	*E*_musc_	Δ*x*_com_	CoT
	°	s	m/s	J/kg	m	J/m/kg
Gait initiation from preferred posture						
B only	0.96 ± 1.06	1.26 ± 0.20	0.92 ± 0.14	2.00 ± 0.27	0.23 ± 0.03	8.98 ± 1.44
Random: B	1.01 ± 1.01	1.25 ± 0.15	0.90 ± 0.15	1.99 ± 0.26	0.23 ± 0.04	9.05 ± 1.56
Random: F	1.00 ± 1.03	1.62 ± 0.21	1.12 ± 0.17	2.27 ± 0.27	0.34 ± 0.04	6.71 ± 0.94
F only	1.00 ± 1.01	1.56 ± 0.23	1.13 ± 0.17	2.25 ± 0.32	0.35 ± 0.05	6.53 ± 0.96
Random: B vs B only	*t*_(19)_ = 0.80 *p* = 0.432	*t*_(19)_ = −0.22 *p* = 0.830	*t*_(19)_ = −1.72 *p* = 0.101	*t*_(19)_ = −0.43 *p* = 0.669	*t*_(19)_ = −0.27 *p* = 0.787	*t*_(19)_ = 0.28 *p* = 0.780
Random: F vs F only	*t*_(19)_ = 0.07 *p* = 0.948	*t*_(19)_ = 1.52 *p* = 0.146	*t*_(19)_ = −1.26 *p* = 0.223	*t*_(19)_ = 0.86 *p* = 0.398	*t*_(19)_ = −1.78 *p* = 0.091	*t*_(19)_ = 2.23 ***p* =** **0.038**
B only vs F only	*t*_(19)_ = −0.62 *p* = 0.543	*t*_(19)_ = −6.02 ***p* <** **0.001**	*t*_(19)_ = −11.29 ***p* <** **0.001**	*t*_(19)_ = −5.21 ***p* <** **0.001**	*t*_(19)_ = −13.65 ***p* <** **0.001**	*t*_(19)_ = 10.77 ***p* <** **0.001**
Gait initiation from target						
B: -1.15°	−1.19 ± 0.09	1.11 ± 0.27	0.95 ± 0.17^**b**^	2.03 ± 0.29^**b**^	0.22 ± 0.04^**b**^	9.60 ± 1.97^**b**^
B: 1.15°	1.06 ± 0.07	1.09 ± 0.21	0.96 ± 0.15	1.99 ± 0.34^**b**^	0.23 ± 0.04^**ab**^	8.85 ± 1.66^**a**^
B: 3.44°	3.34 ± 0.07	1.16 ± 0.22	0.97 ± 0.15^**a**^	2.18 ± 0.37^**a**^	0.26 ± 0.03^**a**^	8.69 ± 1.63^**a**^
F: -1.15°	−1.19 ± 0.08	1.57 ± 0.18^**b**^	1.19 ± 0.22	2.70 ± 0.32^**b**^	0.38 ± 0.07^**b**^	7.34 ± 1.93^**b**^
F: 1.15°	1.05 ± 0.04	1.47 ± 0.25	1.18 ± 0.21	2.29 ± 0.41^**a**^	0.36 ± 0.06^**a**^	6.48 ± 1.41^**a**^
F: 3.44°	3.34 ± 0.07	1.37 ± 0.24^**a**^	1.18 ± 0.20	2.25 ± 0.42^**a**^	0.35 ± 0.06^**a**^	6.67 ± 1.57^**a**^
B: effect of target	−	*F*_(2,38)_ = 1.83 *p* = 0.174	*F*_(2,38)_ = 4.92 ***p* = 0.013**	*F*_(2,38)_ = 10.72 ***p* < 0.001**	*F*_(1.9,35.9)_ = 28.28 ***p* < 0.001**	*F*_(1.4,26.1)_ = 5.11 ***p* = 0.023**
F: effect of target	−	*F*_(2,38)_ = 7.71 ***p* = 0.002**	*F*_(1.4,27.0)_ = 0.88 *p* = 0.394	*F*_(1.3,24.9)_ = 41.66 ***p* < 0.001**	*F*_(1.5,27.7)_ = 16.66 ***p* < 0.001**	*F*_(1.2,23.3)_ = 8.07 ***p* = 0.006**

B: Backward, F: Forward. See Fig 4 for variable definitions. Post-hoc statistics for rmANOVA on targets are provided in S1 Appendix; significant post-hoc tests are also indicated in Fig 4B. ^**a**^ Significant difference w.r.t. -1.15°, ^**b**^ Significant difference w.r.t. 3.44°. Significant differences are highlighted in bold.

Next, we assessed the energetic cost of gait initiation. Our simulations of Experiment 1 showed that activation maintenance costs drive energy expenditure during quiet standing. However, many idealized representations of gait initiation only consider kinetic and potential energy [48–51], overlooking the cost of losses from muscle activation and likely underestimating total energy expenditure. To address this, we used the same musculoskeletal modeling approach as Experiment 1 (Fig 1B) to estimate energy expended by the lower-limb muscles during gait initiation from the prompt (i.e., during standing) to the first foot strike (heel or forefoot). The cost of gait initiation was quantified as the cost of transport (CoT; [11]), computed as the weight-normalized energy expended by the simulated muscles (*E*_musc_) divided by the change in center of mass location in the forward direction (Δ*x*_CoM_, Table 2, Fig 4B). During the sets with known walking direction (Forward and Backward only sets), the CoT of backward walking was ~ 38% higher than that of forward walking (Fig 4B, Table 2). When walking direction was random, the CoT of forward walking increased by ~3% compared to when it was known, whereas there was no difference for backward walking (Table 2). For each direction, there were no significant differences between known or random conditions for the simulated muscle energy expenditure or center of mass distance (Table 2). Together, these results indicate that participants did not adjust their standing posture in anticipation of the expected movement direction, even though we observed a marginal but significant improvement in the energetic cost of forward gait initiation when the direction was known.

### Static lean angle only minimally affects the time and energy expended for gait initiation

In the second block of Experiment 2, we used the target trials to test whether initiating gait from a more anterior or posterior leaning whole-body angle reduces the time required and/or the energetic cost of initiating gait. Participants stood at one of three prescribed whole-body angles (-1.15, 1.15, 3.44°; Fig 1D) and initiated walking in either the forward or backward direction five times each in random order following a prompt (Fig 1F).

During forward gait initiation, the time to reach steady-state velocity varied significantly with lean angle (Table 2), with post-hoc tests showing that participants reached steady-state walking around 0.2 seconds faster from a forward than backward lean angle (Fig 4B, S1 Appendix for statistics). However, comparisons of the outer two targets with the central target were not significantly different, suggesting that postural adjustments beyond the central posture did not yield significantly faster gait initiation times. In contrast, during backward gait initiations, lean angle did not significantly affect time to reach steady-state velocity (Table 2). We further observed that the peak center of mass velocity did not differ across targets for forward initiation, but was marginally (~2%) and significantly higher when initiating backward walking from a forward-leaning posture versus a backward-leaning posture (Table 2, Fig 4B). Overall, these results suggest that the time required to initiate forward gait can be improved slightly with small adjustments to whole-body lean during quiet standing.

Next, we assessed whether lean angle changes the energetic demands of gait initiation. The CoT differed with lean angle for both forward and backward gait initiations (Table 2, Fig 4B). Interestingly, post-hoc tests revealed that for both initiation directions, the CoT was ~ 10% higher when leaning at the posterior target as compared to the central target (Fig 4B), contrary to the hypothesis that posterior lean would facilitate more efficient backward initiation. Conversely, the CoT did not differ between the anterior and central target for both initiation directions (Fig 4B), suggesting that maintaining a posture close to 1.15° was sufficient to optimize the CoT of upcoming gait initiation regardless of direction.

To better understand what drives the difference in CoT between anterior and posterior leaning postures, we examined the total muscle energy expended (*E*_musc_) and the CoM displacement (Δ*x*_com_). The total simulated muscle energy expenditure between the cue to walk and the first foot strike decreased by < 2% when leaning in the direction of movement for both forward and backward initiations (Table 2, Fig 4B, see S2-S3 Figs for simulated muscle activity of all muscles). Differences between the central target and the target aligned with the direction of movement were not significant, whereas initiating gait from a lean angle opposite to the movement direction incurred a higher muscle energy expenditure than both other targets (Fig 4B, S1 Appendix for statistics). In addition, the CoM displacement between the starting position and the first foot strike decreased by around 3–4 cm when leaning further in the direction of movement for both forward and backward trials (Table 2, Fig 4B), likely because the prescribed lean already placed the CoM toward the desired direction. Because CoT is energy expenditure per distance traveled, these combined reductions in both energy and displacement canceled each other out, suggesting that leaning further in the direction of movement does not necessarily improve the energetic cost of gait initiation.

## Discussion

In this study, we examined (i) how metabolic cost varies with lean angle, (ii) how the cost of gait initiation depends on posture, and (iii) whether humans adopt postures that minimize cost during quiet standing and in preparation for walking. In Experiment 1, we found that energy expenditure in standing increased asymmetrically with lean angle, with a minimum at a slight forward lean, and that participants preferred lean angles close to this energetic minimum. In Experiment 2, participants continued to prefer a forward-leaning orientation and did not adjust their posture for gait initiation, even when the upcoming gait direction was known. Consistent with this, our musculoskeletal simulations showed that the energy cost of gait initiation was also near optimal at a slight forward lean, regardless of the direction of gait initiation. Taken together, our results support the idea that humans minimize energy expenditure during quiet standing. However, substantial within- and between-participant variability in preferred posture suggests that minimizing energy expenditure does not require tightly tracking a single energy-optimal reference posture [28,39]. These findings provide insight into how energy expenditure may underlie our everyday movement control and challenge the common modeling assumption that postural costs can be accurately described by assuming a symmetric error from a fixed reference posture (e.g., vertical or static equilibrium when incorporating passive ankle stiffness).

### Preferred human posture aligns with the energetic minimum

The first aim of our study was to determine how energy expenditure varies with standing posture. The alignment of preferred posture with the energetic minimum (Fig 2) suggests that participants naturally adopted an energetically efficient posture during quiet standing. We also found that removing vision did not significantly affect energy expenditure or lean angle, although CoP velocity increased slightly. This indicates that while visual feedback aids postural control (for review, see [27,52,53]), its absence does not substantially alter the selection or energetic cost of the preferred posture. Our results further revealed that the increase in energy expenditure with lean angle was not symmetric (Fig 2A), increasing at about twice the rate during posterior lean as compared to anterior lean. This directional asymmetry in energetic cost likely reflects underlying anatomical and physiological asymmetries in the human body, which may have evolved under selection pressures for efficient (forward) locomotion [54,55] and shape the energetic landscape of standing balance. For instance, the relatively short calcaneus, while advantageous for energy-efficient running [56], limits posterior stability because it is much shorter than the forefoot and therefore requires faster CoP corrections (Fig 2B) to prevent backward falls. Similarly, passive ankle stiffness increases with forward lean [46,57], reducing the proportion of active muscle contributions needed to generate stabilizing torques. Lastly, we found that backward-leaning postures may engage muscles dominated by fast-twitch fibers, which are less metabolically efficient, whereas forward lean more prominently engages muscles with slow-twitch fibers suited for economical tonic activation [32,55]. Together, these biomechanical and muscular properties make it less costly to allow greater excursions in the anterior direction than in the posterior, helping to explain why humans typically adopt a forward-leaning stance.

Additional non-metabolic factors may also drive our preference for a slight forward lean. Sinha and Maki [58] proposed that this orientation simplifies control by only relying on tonic plantarflexor activity, while also increasing the gain of short-latency reflexes through increased tonic activity [59]. A second possibility is that a forward lean positions the center of mass closer to the center of our asymmetric base of support, which may help optimize stability boundaries [60,61]. This preference may also prepare us to initiate movement [43], although our direct examination of this possibility in Experiment 2 showed that participants remained near their energy-optimal stance regardless of the expected walking direction. Other factors not assessed here may also contribute, including stabilizing the head [62], reducing the cognitive demands of control [63,64], or allowing exploratory variability [65]. Thus, while our findings suggest that humans preferentially adopt near-optimal postures from an energetic perspective, the control mechanisms underlying this selection remain unresolved. Possible explanations include optimal control theory [66], synergy- or repertoire-based learning [67,68], or even evolved or learned priors that bias behavior towards sufficiently optimized solutions [69,70].

### Costs of gait initiation do not drive quiet standing posture

The second and third aim of this study were to determine how quiet standing posture affects the cost of gait initiation and whether this cost influences our choice of standing posture. We found that leaning further in the intended direction of movement provided no significant energetic benefit or reduction in time spent initiating gait when compared to the minimum-cost static posture. For forward walking, leaning further forward as compared to a central posture only slightly reduced the time to reach steady-state velocity (Fig 4B) and increased the CoT of the first step by just 0.17 ± 0.49 J/m/kg, consistent with prior reports that posture has little impact on the characteristics of forward gait initiation [44]. For context, humans adjust their steady-state walking speed to minimize CoT differences in the order of ~0.42 J/m/kg (0.1 kcal/km/kg), and continuously expend around 3.8 J/m/kg [6]. Initiating forward gait from the least efficient posture tested (-1.15°), which increased energy expenditure by 0.42 ± 0.27 J/kg relative to initiation from the minimum-cost posture, would raise total expenditure over 10 m of walking by only ~1%. While significant, such marginal differences indicate that the energetic penalties of starting gait from a suboptimal stance may be too small to drive systematic adjustments, especially when compared against the substantial cost increase associated with lean angle (Fig 2A). Consistent with this finding, participants maintained their preferred static posture even when the direction of gait initiation was predictable (Fig 4A). Together, these findings suggest that while posture can modulate the cost and timing of gait initiation at larger lean angles, the influence is negligible relative to the continuous cost of walking and standing, and thus unlikely to shape postural choice under typical everyday conditions.

Instead, time and task constraints may have a stronger influence on postural selection for gait initiation [43]. For example, sprinters adopt a crouched start to maximize acceleration [42,43], while marathon runners primarily start upright. Clearly, urgency can drive changes in posture when the task demands it [43]. In our experiment, participants had no strong incentive to minimize the time spent initiating gait, as their initiation speed did not hinder their ability to perform the task effectively. We hypothesize that stricter time demands, such as reaching the end of the walkway as quickly as possible, would have led them to prioritize acceleration at the expense of energetic efficiency [48]. Future work could assess how humans optimize energy expenditure through postural adjustments during gait initiation tasks with greater time pressure.

### Variability and flexibility in postural control

In both experiments, participants naturally converged on an energetically efficient posture when no target was prescribed, yet also exhibited substantial variability both across participants and within trials (Figs 2 and 4). A Monte Carlo analysis of a passive ankle stiffness model showed that differences in participants’ stiffnesses could explain only part of this variability. The simulated equilibrium angles (where active contributions to the ankle moment are minimal) were slightly more posterior than the actual postures adopted in Experiment 1. This suggests that a participant’s preferred forward lean is not solely the result of minimizing active contributions to the ankle moment, consistent with earlier work showing that humans typically adopt a more forward-leaning stance than predicted by inverted pendulum models [23,71] and that ankle stiffness alone cannot stabilize the human body [72–74].

We further found that both metrics for within-trial postural variability (i.e., standard deviation of lean angle and CoP velocity, Fig 2B) did not differ across central targets (0 to 3.44**°**). This aligns with recent work showing that variability in postural control signals (i.e., ankle torque) remains relatively constant within this range, but rises beyond it, likely because higher force demands increase motor noise [75]. Furthermore, when standing at preferred postures, the within-trial standard deviation of lean angle was roughly twice that of the minimum-cost target (Fig 2B, Table 1), consistent with larger postural variability and low-frequency sway previously reported in unconstrained standing [76–79]. This greater dispersion of lean angles did not coincide with higher CoP velocity or metabolic cost (Fig 2), suggesting that the target successfully constrained the natural sway without substantially increasing the control demand. Strictly limiting the permitted CoM or CoP variability can increase energetic expenditure due to the higher incidence and magnitude of postural corrections [28,39], though energetic costs also rise when variability is artificially increased using more challenging experimental conditions [38]. Intermittent balance control models [80–82] formalize this tradeoff between effort and variability by permitting postural sway until a stability threshold is exceeded, thereby reducing energetic demands compared to continuous control [83]. Overall, minimizing variability does not appear to be a prerequisite for minimizing energy expenditure, suggesting that efficient postural control does not require constraining posture at a single energetic minimum. Furthermore, our findings suggest that interpretations of postural control without a fixed reference angle, such as intermittent control [80–82] or random walk (i.e., Brownian motion, [77–79]), are not at odds with energetically optimized standing as long as posture remains within the central region of measured costs (Fig 2). The implications of these observations on modeling methods are discussed further below.

### Towards improved models of cost in posture

Postural control is commonly simplified as a continuous minimization of deviations from a reference lean angle of an inverted pendulum [21–24], resulting in a symmetric linear or quadratic cost function with a single optimum in the center. However, our experimental data show that energy expenditure increases at almost twice the rate with backward lean than with forward lean, which is not captured by these cost functions. To improve the predictive and exploratory power of inverted pendulum models, we recommend penalizing posterior-leaning postures more strongly than anterior-leaning postures to account for the asymmetries observed in energy expenditure (Fig 2A) and to use caution when equating the idealized ankle moment to physiological effort. Future work could evaluate whether multi-objective control models (e.g., a linear quadratic regulator minimizing control action and kinematic state [21,24,28,30]) and intermittent control [80–82] can be improved by incorporating asymmetric weights to control actions in the anterior and posterior directions, which may better represent the cost of accelerating the body in these directions. We hypothesize that this adjustment may explain the human tendency to maintain an anterior-leaning posture during quiet standing as a precaution to avoid the steeper cost gradient associated with a posterior lean. Furthermore, our work suggests that energy expenditure in posture is sufficiently optimized by limiting sway within a central region of lower-cost lean angles, providing experimental support for balance interpretations that do not rely on a reference angle to optimize control (e.g., intermittent control [80–82] or Brownian motion [77–79]). More broadly, it is also important to consider whether a model’s mechanical representation may limit inferences about energy expenditure. A single-link inverted model cannot capture multi-segment coordination and may not accurately represent higher-frequency dynamics [30,84], possibly underestimating the control effort and highlighting the need to select appropriate model mechanics and cost functions for the problem at hand.

Alternatively, musculoskeletal simulations may offer an approach grounded in anatomy and muscle physiology to represent the energetic costs of posture. Such models have been applied to simulate quiet standing [25,26,85,86] and estimate the energetic cost of postural responses [87]. Our study extends this work by validating musculoskeletal inverse simulations as a computational approach for capturing trends in measured energetic cost of standing across lean angles, including the asymmetric increase for posterior lean. While the model estimated the shape of the metabolic cost landscape, it underestimated the increase in energetic cost for anterior lean angles (Fig 2A). One possibility is that contributions from muscles not included in the model, such as those of the back, arms, and neck, increase energy expenditure during anterior postures. The measured EMG activity of the erector spinae muscle (Fig 3) showed a small increase in activity with more anterior postures, suggesting that it could contribute to changes in energy expenditure together with other trunk muscles, though this trend was considerably smaller than that observed in the plantarflexor muscles. Another explanation is the model’s high passive ankle stiffness: for a participant with parameters close to the unscaled model (male, 1.77 m, 80 kg), the summed tendon force of the inactive soleus and gastrocnemius (medialis and lateralis) muscles generated 400 N at a plantarflexed ankle angle of 5 degrees, resulting in a moment of 17 Nm. By comparison, previous literature reported the moment contribution of passive ankle stiffness to be around 10 Nm at this angle [46,57], suggesting that the model’s larger passive contributions may reduce the required plantarflexor muscle activations for maintaining equilibrium. Future work could investigate how stabilization of the spine and upper body, individual (sex) differences in morphology and muscle strength [88,89], weight distribution, physiological and behavioral changes with ageing [61,90], and passive ankle stiffness [46,72,91] affect energy expenditure and optimal posture.

## Conclusion

By combining indirect calorimetry and musculoskeletal simulations, we quantified the energetic cost of quiet standing and gait initiation across a range of natural standing postures. Participants maintained their preferred posture close to the minimum-cost lean angle during static standing. The anticipation of gait initiation in either direction did not alter this preference, likely because small adjustments in lean angle only lead to changes in energetic cost and duration that are inconsequential compared to continuous walking. These findings provide insight into how energy expenditure drives our everyday balance control and challenge the common modeling assumption that postural costs can be accurately described by symmetric errors from a single reference posture. By integrating empirical and simulation-based approaches, our work offers a broader perspective on the cost functions that define optimal postural control and highlights opportunities to improve them.

## Materials and methods

### Ethics statement

All experiments were conducted in accordance with the Declaration of Helsinki and under the approval of the Erasmus Medical Center Medical Ethics Review Committee (MEC-2023-0205). Participants received the information letter of the experiment at least 24 hours prior to participating. Before the experiment was conducted, participants provided written informed consent for their participation, and all participants provided the optional permission to publish the coded data collected during the experiment under a Creative Commons Attribution (CC BY) license.

### Participants

A total of 33 healthy adult participants aged 18–50 with no history of balance, neurological or musculoskeletal disorders were recruited for this study (Experiment 1: 8 male, 5 female, age = 24.2 ± 2.1 years, height = 175.3 ± 7.3 cm, weight = 72.4 ± 9.7 kg; Experiment 2: 9 male, 11 female, age = 24.1 ± 3.3 years, height = 178.1 ± 11.1 cm, weight = 70.0 ± 10.5 kg, one participant completed both experiments).

### Methods and protocol

This study aimed to determine the energy expenditure required for maintaining a static posture and initiating gait. We performed two experiments: (1) a metabolic assessment of static standing at six prescribed target positions and at preferred postures with eyes open and closed, and (2) a walking initiation experiment where participants stood ready and started walking when presented with a cue.

#### Experiment 1: Static standing protocol.

The aim of Experiment 1 was to measure the metabolic cost of anterior and posterior postures and to determine whether this cost could be predicted from motion data using a musculoskeletal model of human standing. A motion capture system (Qualisys AB, Göteborg, Sweden), consisting of 12 Oqus 700 cameras, was used to track the locations of 43 passive motion capture markers placed on the participant at 100 Hz. Marker placement followed the IOR gait marker set (used by [92]) with the addition of four markers on the head, two on each arm, and the omission of the T7 marker due to obstruction by the back-worn metabolic analyzer (See S1 Appendix for list of markers). Ground reaction forces on both feet were measured at 500 Hz using two force plates (9260AA6, Kistler Group, Winterthur, Switzerland; CoP error < 2.0 mm), which were zeroed before each set of measurements (four sets per participant) to account for potential signal drift.

Energy expenditure was measured throughout the experiment using a breath-by-breath indirect calorimetry metabolic analyzer (K5 9260AA6, COSMED, Italy). This type of indirect calorimetry system has been validated during resting conditions against Douglas bag measurements and metabolic simulators [93,94]. Before each session, the device was calibrated following the manufacturer’s instructions using a certified gas mixture (16% O_2_, 5% CO_2_), a CO_2_ scrubber (C04408-01-07, COSMED, Italy), and a 3 L calibration syringe (C00600-01-11, Hans Rudolph, United States). The analyzer recorded minute ventilation (VE) as well as the O_2_ and CO_2_ concentrations in both inhaled and exhaled air, and reported energy expenditure using the Brockway equation [95]. Caloric and caffeine intake are known to affect metabolic assessments [96], so participants were asked not to consume caffeine or food for at least 3 hours prior to the experiment’s start time. In addition to this measurement, the energy expenditure was also computed using musculoskeletal simulations (see Analysis). Indirect calorimetry data were excluded for one participant (S09) because their ventilatory equivalent (VE/VO2) was highly elevated compared to that of the other participants (26.0 ± 4.0 for the remaining included participants, around previously reported rest ranges of 25–30 [97]).

Electromyography (EMG) data were collected from 8 muscles on the right side of the body, including the medial gastrocnemius, soleus, tibialis anterior, vastus medialis, rectus femoris, biceps femoris, semitendinosus, and erector spinae. The ground electrode was connected to the right ankle on the lateral malleolus, and the SENIAM 8 guidelines [98] were used for electrode placement. Signals were amplified with a gain of 20 and recorded at 2000 Hz using an ExG amplifier (Porti7-8b8at, TMSi, Netherlands). The amplifier was connected to a laptop computer (Windows 10, USB 3.0, i7-1195G7, 16 GB RAM, MSI, Taiwan) using a USB connector to start, stop, monitor, and store the EMG data using a modified version of the TMSi MATLAB interface. The measurements were normalized to the maximal observed activation during exercises described by Uhlrich et al. [45,99] to evoke a maximal voluntary contraction. These exercises consisted of prone knee flexion, supine ankle dorsiflexion, hip flexion kicks, and maximum height jumps with knees initially at ~90°. A prone lumbar extension exercise without arm support was added to maximize activation of the erector spinae. A synchronization signal was sent from the “sync out” port on the first Oqus camera to the trigger port of the EMG amplifier at the start of each measurement, allowing for the synchronization of the two measurements. The indirect calorimetry measurement was synchronized by manually reading the device measurement time at the start of each measurement.

At the start of the experiment, participants were shown the equipment and explained the protocol before signing the informed consent. A short survey was used to collect basic participant information, including age (24.2 ± 2.1 years), sex (8 male, 5 female), dominant hand (12/13 right), and dominant leg (12/13 right, based on which leg they would use to kick a ball). General physical activity was assessed using the short International Physical Activity Questionnaire (Aug 2002 English version) to determine a three-level scale [92,100]. Almost all participants (11/13) were rated at the high activity level, while the remaining participants were rated at the middle level. This distribution was insufficient for making informative comparisons between scores. Next, basic anthropometric measurements were collected, including body length (175.3 ± 7.3 cm), ankle height at the lateral malleolus (6.8 ± 0.6 cm), and hip width at the greater trochanter (34.2 ± 2.1 cm). Body weight (72.4 ± 9.7 kg) was calculated by summing the vertical forces on the force plates and subtracting the weight of the metabolic analyzer (900 g). The foot placement on the force plate was standardized by positioning the centers of the ankle joints at hip width, estimated as 0.532 times the measured distance between the greater trochanters following Bennett et al. [101]. The center of rotation of the ankle joint was assumed to lie at the anterior edge of the medial malleolus [102]. During quiet standing, whole-body lean was computed through a static inverted pendulum approximation, where the CoM was assumed to be directly above the CoP. The length of the pendulum was approximated as the center of mass height with respect to the ankle joint (*h*_CoM_), estimated by multiplying the body height by 0.56 for female and 0.57 for male participants [103], and subtracting the ankle height. The lean angle *θ* was then estimated in radians by dividing the distance of the CoP with respect to the ankle joint [102] by the center of mass height.

Experiment 1 consisted of two trial types: preferred posture trials and target trials (Fig 1C-1E). In preferred posture trials, participants stood quietly facing forward with their arms at their sides, maintaining a comfortable posture with their arms by their side (but not in their pockets) with either their eyes open (EO) or closed (EC). Each trial lasted five minutes. In target trials, participants were instructed to match their CoP to a visual target displayed on a screen 3.20 m in front of them, at a height of 1.67 m. This target was necessary to limit natural drifts in quiet standing posture [76–79], which would invalidate the indirect calorimetry assessment of a specific lean angle. A black circle represented the target, and participants controlled a red cursor corresponding to their CoP location. The cursor turned green when it was within the target area, providing real-time feedback. While the target circle was always drawn in the center of the screen, participants had to maintain a lean angle *θ*_target_ of -2, 0, 2, 4, 6, or 10 ⋅ 10^-2^ rad (i.e., -1.15, 0, 1.15, 2.29, 3.44, 5.73°) to get the cursor in the center. These lean angles correspond to a subset of the targets used by Mensink et al. [75], where the variability of ankle torques was investigated. The corresponding CoP target positions were calculated as:


$$\begin{array}{c} \begin{array}{c} x_{\text{target}}=h_{\text{CoM}}\cdot \theta_{\text{target}}. \end{array} \end{array}$$ (1)

The radius *r*_target_ of the target circle was normalized to body length so that a similar whole-body angle excursion would result in a similar deviation from the target for all participants. The target radius is shown in Fig 2B to allow direct comparison with the natural within-trial variability of lean angle, indicating whether participants successfully restricted their spontaneous sway to the target range. The radius of the target was computed as


$$\begin{array}{c} \begin{array}{c} r_{\text{target}}=h_{\text{CoM}}\cdot \frac{\pi }{\text{1}\text{8}\text{0}\circ }(\text{0}.\text{3}\circ ). \end{array} \end{array}$$ (2)

The circle was green when the participant was within the radius of the target, computed as


$$\begin{array}{c} \begin{array}{c} \sqrt{{\left(x_{CoP}-x_{\text{target}}\right)}^{\text{2}}+\overset{\text{2}}{\underset{CoP}{y}}}<r_{\text{target}}. \end{array} \end{array}$$ (3)

A 5-minute break was scheduled midway through the experiment. To minimize order effects, the order of the blocks (Preferred or Target) was reversed before and after the break. A total of 3 out of 208 trials were excluded due to technical issues or experimenter error (EO and targets 0 and 1.15°, all from different participants). In these cases, participant mean data were computed using the remaining valid trial of that condition.

#### Experiment 2: Walking initiation protocol.

The aim of Experiment 2 was to determine the energetic cost of gait initiation across natural postures and to evaluate whether humans select postures that minimize this cost based on the expectation of walking direction. Participants (9 male, 11 female, Age = 24.1 ± 3.3 years, Height = 178.1 ± 11.1 cm, Weight = 70.0 ± 10.5 kg) stood on a force plate and initiated gait when a prompt on a screen asked them to start walking in the forwards or backwards direction (Fig 1F-1G). Because the initiation of walking takes far less time than the time constant of indirect calorimetry (~42 seconds [104]), the metabolic cost of gait initiation could not be measured. Instead, we estimated metabolic cost using musculoskeletal simulations on the kinetic and kinematic data measured through the force plate and motion capture system (see *Computation of metabolic cost* below).

The motion capture system consisted of 8 cameras running at 100 Hz (4 Miqus M3 and 4 Miqus M5, Qualisys AB, Göteborg, Sweden), an analog measurement board (Qualisys Analog Interface 16 Channels, 230597, Qualisys AB, Göteborg, Sweden), a sync box (Qualisys Sync Box, 410850, Qualisys AB, Göteborg, Sweden), and a single force plate running at 1000 Hz (Plate: BMS 400600HF-1K, Amplifier: Optima OPT-SC, AMTI, MA, US; CoP error < 0.5 mm) embedded in a wooden walkway (740 cm long, 80 cm wide, > 2mm clearance gap with the force plate and wooden walkway). The foot placement and motion capture marker locations were identical to those used in Experiment 1, with the addition of a marker on the T5 vertebra, which could now be placed since the back-worn metabolic analyzer was no longer used (see S1 Appendix for list of markers). A TV screen was placed at eye level at the anterior-facing end of the walkway (3.2 m away) to show the CoP target in some trials and to prompt the participant to start walking at the start of the trial. The data was stored in 12-second trials, with the prompt appearing at the 6-second mark.

Experiment 2 consisted of two sets of trials: preferred and target posture trials. The preferred posture set was always performed first to avoid influencing the participants’ preferred posture at gait initiation. In the preferred trials, the participant was instructed to stand calmly with their arms resting at their sides while looking straight ahead. During these trials, there were three distinct blocks to evaluate whether the known probability of gait initiation influenced posture: Forward-only (20 trials), Random (15 trials forward, 15 trials backward), and Backward-only (20 trials). In all three blocks, the participant was explicitly informed of the probabilities associated with the prompts, but not as to when they would be delivered. In the Random block, a random permutation determined the order of the prompts, and neither the participant nor the experimenter (to prevent experimenter bias) was aware of the direction presented for each trial.

In the set with target trials, participants received the same visual feedback on CoP position as used in Experiment 1 (Fig 1D, Eq 1–3), but only at target locations of -1.15, 1.15, and 3.44°. For each target, participants were prompted to walk forward or backward 5 times each, resulting in a total of 30 trials. The trials were ordered using a random permutation, ensuring that the target order and initiation direction were unpredictable. Trials that contained an error, such as initiating gait in the wrong direction or with the incorrect leg, were repeated and shuffled among the last five trials so that participants could not predict which direction would be repeated.

Prompts to initiate walking were provided using the TV screen. Forward trials were indicated by a blue screen displaying “Forward”, an upward-facing arrow, and a sound tone of 532 Hz; backward trials were indicated with an orange screen displaying “Backward”, a down-facing arrow, and a 440 Hz sound tone. After 3.1 seconds, a stop tone of 329 Hz was played to signal to participants that the trial had been completed. The period was selected to approximate the time by which participants should have made their fourth foot strike. Participants were asked to step with the same leg consistently and could choose a different leg for forward or backward walking. The time between a participant being ready and the delivery of a prompt was standardized to be a random integer in the range of 5–10 seconds, so that the timing of the prompt would not be predictable. The participant was considered ready when the absolute time derivative of the CoP remained below a threshold for 1000 consecutive samples (1000 ms). This threshold ${\dot{c}}_{\text{threshold}}$ was determined at the start of the experiment using a baseline trial of 2 minutes and computed as:


$$\begin{array}{c} \begin{array}{c} {\dot{c}}_{\text{threshold}}=\text{3}\cdot \text{std}\left(\dot{c}\right), \end{array} \end{array}$$ (4)

where $\dot{c}$ is the vector containing the CoP time derivative of the entire baseline trial. A MATLAB script was used to detect when the participant was ready and start a timer, indicating to the experimenter that the prompt could be sent.

### Quantification and statistical analysis

#### Data analysis.

The average lean angle was extracted from CoP data using Eq 1. Further kinematic data were derived from musculoskeletal simulations (see below).

Raw EMG recordings were high-pass filtered (4th order zero-phase Butterworth, cutoff frequency 10 Hz), full-wave rectified, and then low-pass filtered (4th order zero-phase Butterworth, cutoff frequency 100 Hz). The erector spinae signal was high-pass filtered at 60 Hz instead to minimize electrocardiographic interference [105]. The EMG signal for each muscle was normalized by dividing the signal by the maximum observed EMG signal during that participant’s maximum voluntary contraction exercises, which were additionally low-pass filtered at 6 Hz before computing the maximum [45].

Indirect calorimetry data were only used beyond the first 60 seconds of each recording, given that the time constant for this type of measurement is approximately 40 seconds [104] and that previous work shows that steady-state energy expenditure is achieved in standing during the first minute [106]; see S1 Appendix for validation. All metabolic measurements were normalized to body mass and reported in W/kg. The basal metabolic cost (i.e., the energy expended by non-motor processes to uphold bodily function) is generally assumed to be constant [107] and requires a prolonged period of rest and longer fasting to be measured accurately [34]. Its contribution is commonly negated by subtracting the cost of a standing baseline trial from other activities, although this may underestimate the cost of those activities, as standing balance requires energy [107]. In Experiment 1, the metabolic cost in the eyes-open preferred posture trial was subtracted from the other trials for both the measured and simulated energy expenditure separately for visualization purposes only. Doing so eliminated the offset between measured and simulated energy expenditure caused by the basal rate and muscle activity in the baseline trial, allowing for comparison of relative cost differences across different postures. Breath-by-breath samples lasting shorter than one second or having a respiratory quotient larger than 2 or smaller than 0.4 were omitted from the mean energy expenditure (0.69% of samples). The 95% confidence interval of the mean energy expenditure was computed using bootstrapping by resampling the participant means 10,000 times and taking the 2.5th and 97.5th percentiles of the resulting distribution. Time-dependent measures of breathing (e.g., energy expenditure per second) were averaged using the breath duration for a weighted average, as a sample-based average of breath-by-breath data would overvalue the contribution of short breaths. Finally, we estimated the slope of energetic cost with lean angle using the mean finite-difference over the range of consecutive lean angles from the minimum-cost target to the largest measured posterior or anterior lean angles.

In Experiment 2, the steady-state velocity of walking following gait initiation was determined as the maximum absolute velocity of the center of mass before the end of the trial. The center of mass location was approximated using the inverse kinematics of the musculoskeletal model (see below). The time to reach steady state was determined as the time required to reach 90% of that participant’s average steady-state velocity. The time was reported in relation to the onset of movement to account for possible variations in reaction time. The onset of movement was determined as the first point in time where the magnitude of the CoP velocity in the lateral direction (due to the shifting of weight to one foot) exceeded the mean plus four times the standard deviation, which was calculated from the three seconds preceding the presentation of the prompt.

#### Musculoskeletal analysis of motion capture data.

Motion capture trajectories were processed using Qualisys Track Manager (version 2024.2, Qualisys AB, Göteborg, Sweden). A prediction error of 20 mm and a maximal residual of 5 mm were accepted in the 3D tracking phase. Gaps in motion capture markers that can occur due to temporary occlusion were gap-filled using a polynomial interpolation if the occlusion lasted for at most 10 samples (100 ms). Markers that were occluded for longer than 100 ms were not gap-filled; in that segment of the measurement, only other valid markers were used for the subsequent inverse kinematics step (see below). The fill percentage was 99.9 ± 0.3% in Experiment 1 and 99.9 ± 0.5% during the simulated part of gait initiation in Experiment 2. The standard deviation of the wand length during calibration was < 1.1 mm for all measurements.

Musculoskeletal analyses were performed using OpenSim [14] (version 4.3) with the Rajagopal lower limb musculoskeletal model [13]. This model was selected because it provides improved representations of lower-limb muscle-tendon geometry and fiber length, particularly of the plantarflexors, and has been widely used to simulate human gait (e.g., [45,108]). First, the model was scaled to the participant’s anthropometrics using the OpenSim scale function, and an inverse kinematics analysis was performed to solve for the joint angles of the participants during standing and walking. Marker trajectories were low-pass filtered at 6 Hz. The weights used for the markers in the musculoskeletal scaling and inverse kinematics steps were empirically set, using the default settings [14,109] as a starting point (values provided in S1 Appendix). To ensure that the foot was level with the ground for all participants after scaling, an ankle angle was prescribed per participant in the scaling step (5.24 ± 2.13°, weight 10) such that the foot of the resulting scaled model had a pitch angle close to 0° during standing. The headband markers were not used for inverse kinematics, as the head and torso were assumed to be a single rigid segment. For some female participants (Exp 1: 3/5, Exp 2: 5/11), the placement of the xiphoid process marker was hindered by anatomy and clothing, making it invisible to the motion capture cameras. In these cases, the marker was placed midway between the navel and the xiphoid process. This marker was assigned a low weight in all participants during scaling to accommodate the varied placement.

Inverse dynamics was then used to estimate the ground reaction forces. This estimation can be subtracted from the measured ground reaction forces to calculate the residual forces, serving as a metric for the simulation’s quality. Hicks et al. [47] recommend that the residual forces are less than 5% of the maximally measured net external force during that trial, and the residual moments less than 1% of the center of mass height times the maximally measured net external force. In Experiment 1, the median normalized residual, defined as the residual force or moment divided by the thresholds described above, was 0.101 (no unit) for residual forces and 0.363 for residual moments. The 95^th^ percentile of the normalized residual was 0.183 and 0.699, respectively, indicating that the simulations satisfy the recommended residual accuracy [47]. In Experiment 2, the median normalized force and moment residuals were 0.182 and 0.431, with the 95^th^ percentile at 0.564 and 1.709. Here, the ground reaction forces were estimated using one force plate, which likely contributed to the normalized residual moments exceeding the threshold in some samples. Validation for the accuracy of the force splitting estimation is presented in the S1 Appendix.

Static muscle optimization was then performed at 20 Hz to estimate muscle activations by minimizing the squared activation [110–114]. This approach estimates a set of muscle activations that can generate the required joint torques at each time point. Minimizing activation squared shares the load between muscles, but also assumes the ability to independently control single muscles without any neural constraints (for review, see Ting et al. [115]). The default implementation of the static optimization algorithm in OpenSim assumes (i) that the tendon is infinitely stiff, and (ii) that the parallel element of the Hill-type muscle does not contribute to the tensile force. However, passive stiffness around the ankle joint affects muscular effort during standing [116]. Therefore, the static optimization implementation by Uhlrich, Jackson [45], which includes the stiffnesses of the parallel element and the tendon, was used instead. This calculation uses the tendon stiffness provided in the musculoskeletal model [13] and optimizes the cost function in MATLAB.

To verify simulated muscle activity, it was compared to the EMG data of 7 major muscles contributing to postural control in Experiment 1. The erector spinae was measured but not simulated, so it was compared against the simulated lumbar extension moment between the hip and torso instead. Hicks et al. [47] caution that EMG data should primarily serve to estimate the onset time of muscle activity and/or the trends between conditions, as data is hard to normalize and sensitive to measurement errors. Here, we compared the normalized EMG to the muscle activations to assess whether the increase in simulated muscle activity over lean angles matched the measured activation.

#### Computation of metabolic cost.

The metabolic cost was estimated using the computed muscle activation from the OpenSim simulations. We used the metabolic energy model by Umberger et al. [32], which accounts for losses due to activation and maintenance, shortening, and mechanical work for each muscle independently. The activation and maintenance expenditure depend on the current fiber length of the muscle, with passive contributions reducing energy expenditure when the fiber length exceeds the optimal length [32]. Fiber lengths were extracted from the OpenSim simulations, and optimal fiber lengths were derived from the model [13]. Because fast-twitch (type II) fibers are metabolically less efficient than slow-twitch (type I) fibers [32,55], each muscle’s energy cost was weighted by its ratio of slow-twitch muscle fibers. The muscle-specific fiber-type distributions were based on simulation code by Uchida et al. [117], who used data by Johnson, Polgar [31]. Muscle mass was set using the values reported by Klein-Horsman et al. [33] and scaled proportionally to each participant’s body weight.

The Umberger model predicts a decrease in energy expenditure when muscle activation exceeds excitation. Unlike activation, excitation is not constrained by activation dynamics and is not computed in static muscle optimization methods as they assume idealized muscle activations without activation dynamics [111,118,119]. Prior work shows that static methods produce similar results to dynamic methods (i.e., methods that compute excitation signals iteratively [120]) even during walking and running [111], while incurring far higher computational costs [121]. The assumption that activation and excitation did not affect simulation results was verified by computing the maximum possible error between activation and excitation (see S1 Appendix).

The shortening heat rate was determined by identifying concentric versus eccentric contraction phases based on fiber length changes. Mechanical work was calculated as the product of muscle force and shortening velocity. The MATLAB analysis code, computation of energy expenditure, scaled OpenSim models, raw and analyzed data, and setup files for all OpenSim analyses performed were made publicly available (see Data availability statement).

#### Estimation of ground reaction forces from single force plate data.

In Experiment 2, a single force plate was embedded in a long walkway extending in the forward and backward direction to allow gait initiation in either direction. Because only one plate was available, individual foot ground reaction forces were reconstructed using a kinematics-assisted force-splitting algorithm, which was validated against dual-force plate data (see S1 Appendix). To estimate the reaction forces under each foot during standing, we combined kinematic data from foot markers with the measured global CoP. The CoP for each foot was assumed to lie along a line connecting the calcaneus (CAL) and second metatarsal (MT2) markers, projected onto the ground plane. The individual foot CoPs were estimated by identifying the shortest segment connecting this line to the measured global CoP, using constrained optimization. The normal force was proportionally distributed between the feet such that the moment in the ground plane was zero. This method was validated using an independent dataset with dual force plate measurements of 160 gait initiation trials (see S1 Appendix for validation and detailed methods). Because reliable force estimates were only available before the first foot strike, static muscle optimization was performed only during this initial phase of gait initiation.

#### Monte-Carlo analysis of ankle stiffness and equilibrium lean angle.

To investigate whether individual differences in passive ankle stiffness could account for variations in preferred lean angle (Experiment 1), we estimated how this variability affects the equilibrium whole-body lean angle. This analysis helps infer whether individual differences in the mechanical properties of the ankle could lead to different energetically optimal postural lean angles. We performed a Monte Carlo simulation based on the parameter distributions for ankle stiffness reported by Moseley et al. [46], as this model includes the progressive increase of ankle stiffness with ankle angle [46,57] and provides the uncertainty ranges of the fit parameters, allowing the stochastic generation of ankle stiffness curves. The equilibrium lean angle was defined as the whole-body angle at which the moment produced by passive ankle stiffness balanced the gravitational moment acting on the body. The mass-normalized ankle stiffness of one ankle was modeled as


$$\begin{array}{c} \begin{array}{c} M_{norm}=\frac{e^{k\theta +b}}{m}, \end{array} \end{array}$$ (5)

Where *θ* is lean angle in degrees, *k* = 56.09 · 10^-3^ ± 8.36 · 10^-3^, *b* = 1.871 ± 0.282, and *m* = 68.5 kg. These values were obtained from the study [46] and normalized by dividing the ankle moment by the average mass. We generated 50,000 samples from the distributions of *k* and *b*, assuming a normal distribution for both. Next, we computed the equilibrium angle *θ* for each combination by finding the intersection of the mass-normalized ankle moment (*M*_norm_(*θ*)) and gravitational moment (*gLθ*) using MATLAB for an assumed body with the mean parameters of the participants in Experiment 1 (center of mass height = 0.954 m, weight not required). Because the resulting distribution of equilibrium angles was skewed, we reported the median and interquartile range of the estimated equilibrium angles.

#### Statistical tests.

Statistical analyses were performed in JASP [122] (repeated-measures analysis of variance; rmANOVA) and MATLAB (*t*-tests), using a significance level of 0.05. Variables are expressed using mean ± standard deviation unless mentioned otherwise. Because all conditions were measured within participants, statistical tests were performed using within-subject comparisons. Factors with more than two levels were analyzed using rmANOVA, and specific hypotheses were evaluated using paired-samples *t*-tests. Degrees of freedom are reported between brackets for *t*-tests and rmANOVA outcomes. When sphericity was violated, Greenhouse-Geisser corrections were applied. A Holm correction was applied to all planned post hoc comparisons, with corrected *p*-values indicated by a subscript where applicable. This method does not require independence among hypotheses and is therefore suitable for repeated-measures designs.

In Experiment 1, we assessed how lean angle affected standing by analyzing how energy expenditure (measured and simulated), CoP velocity, and lean angle SD varied across the six target lean angles (Table 1). If the main effect was significant, univariate post-hoc comparisons between targets were conducted. For energy expenditure (measured and simulated), post hoc tests were limited to using one-sided *t*-tests comparing each target to the target with the minimum energetic cost (1.15°). Next, we compared the eyes-open preferred posture trial against the preferred posture with eyes closed and the minimum-cost target to assess the influence of vision and the target, respectively. Lastly, we compared the slopes of energetic cost change over lean angle between the anterior and posterior directions to assess how bodily asymmetry affects the rate of energetic cost increase.

In Experiment 2, we analyzed the lean angle prior to gait initiation along with various additional gait initiation metrics (time to reach steady-state, peak velocity, energy expended, center of mass displacement, cost of transport). To test how prior knowledge of walking direction influenced behavior, we compared the Random block to the Forward-only and Backward-only blocks. Additionally, we compared the Forward-only and Backward-only blocks to assess differences between forward and backward gait. To examine the effect of the three prescribed lean angles on forward and backward gait initiation during Target trials, we analyzed the target trials using separate one-way rmANOVAs. Forward and backward initiations were analyzed separately because the functional meaning of a given lean angle depends on walking direction (e.g., a backward lean is congruent for backward but incongruent for forward walking), leading to crossover interactions that would complicate interpreting a two-way ANOVA.

## Supporting information

S1 Fig Experiment 1 simulated muscle activations during standing. Figure showing the simulated muscle activations of all 80 muscles during the target and preferred posture trials of Experiment 1, compared against EMG data where available. Labels correspond to muscle name abbreviations used in the Rajagopal, Dembia [13] musculoskeletal model.
(PDF)

S2 Fig Experiment 2 simulated muscle activations during forward gait initiation. Figure showing the simulated muscle activations of all 80 muscles during forward gait initiation, split between the stance and swing leg, and for each starting lean angle.
(PDF)

S3 Fig Experiment 2 simulated muscle activations during backward gait initiation. Figure showing the simulated muscle activations of all 80 muscles during backward gait initiation, split between the stance and swing leg, and for each starting lean angle.
(PDF)

S1 Appendix Supplemental methods and validation of assumptions. Contains validation of the force plate splitting algorithm, validation of the indirect calorimetry trial length, validation of the assumption that activation and excitation are similar in slow movements, a list of used motion capture markers and their weights for scaling and inverse kinematics simulations, and post-hoc statistics following significant rmANOVA main effects.
(PDF)
