# Supplementary material for: The energetic cost of human standing balance and gait initiation over a range of natural postures
Source: PLoS Comput Biol. 2026 Jul 7;22(7):e1013522. doi: 10.1371/journal.pcbi.1013522 (PMC13340847; doi:10.1371/journal.pcbi.1013522)
Supplement: S1 Appendix — Contains validation of the force plate splitting algorithm, validation of the indirect calorimetry trial length, validation of the assumption that activation and excitation are similar in slow movements, a list of used motion capture markers and their weights for scaling and inverse kinematics simulations, and post-hoc statistics following significant rmANOVA main effects. (PDF) [file pcbi.1013522.s004.pdf]

S1 Appendix. Supplemental Methods and Validation of Assumptions.

Article: The energetic cost of human standing balance and gait initiation over a range of natural postures

Matto Leeuwis[1], Nikki van Aerts[1], Ajay Seth[2], Patrick A. Forbes[1,*]

[1]Department of Neuroscience, Erasmus MC, University Medical Center Rotterdam, Rotterdam, The Netherlands
[2]Department of Biomechanical Engineering, Delft University of Technology, Delft, The Netherlands
***** p.forbes@erasmusmc.nl

This file contains validation of the force plate splitting algorithm, validation of the indirect calorimetry trial length, validation of the assumption that excitation and activation are sufficiently similar in slow movements for energetic cost estimation, and a list of used motion capture markers and their weights for scaling and inverse kinematics simulations.

Appendix A: Algorithm for Splitting Ground Reaction Forces from a Single Force Plate 2
Appendix B: Validation of trial length of indirect calorimetry measurements in Experiment 1 6
Appendix C: Validation of the assumption that excitation and activation are similar in slow movements 8
Appendix D: List of motion capture markers 10
Appendix E: Experiment 1 statistics tables 11
Appendix F: Experiment 2 statistics tables 14
References 17

Appendix A: Algorithm for Splitting Ground Reaction Forces from a Single Force Plate

Overview

This appendix outlines the algorithm used to estimate the individual ground reaction forces (GRFs) for each foot under experimental conditions where only one force plate was available (i.e., Experiment 2). Under these conditions, we could not directly measure the contributions from each foot. To estimate the ground reaction forces from each foot separately, we developed a simple ground reaction force decomposition approximation that utilizes motion capture and force plate data.

Problem definition

The force plate measures the total force exerted by the body on the ground, as well as the torque (or moment) around the center of the plate. From these measurements, we can calculate the center of pressure (CoP). To estimate how the pressure is distributed under each foot, we define the foot's position as a straight line between two markers: one on the heel (CAL) and one on the ball of the foot (second metatarsal, MT2). This line roughly follows the inner edge of the area where the foot applies pressure; i.e., the functional base of support (1). For simplicity, we assume that the CoP under each foot lies somewhere along this line.

We then describe the position of each foot's CoP using a number between 0 and 1, representing a fraction of the distance from the heel to the toes. The combined CoP for both feet (i.e., the global CoP) is calculated by averaging the individual foot CoPs, weighted by how much force each foot applies. As a result, the global CoP must lie somewhere along the line connecting the two foot-specific CoPs.

However, because we allow the CoPs of each foot to vary independently along their respective lines, we need a constraint to make sure their combination matches the actual global CoP measured by the force plate. We define this constraint using a geometric condition: if the three points (left foot CoP, right foot CoP, and global CoP) lie in a straight line, the cross product of the vectors between them is zero. This constraint ensures that the estimated foot CoPs are physically consistent with the measured force data.

Optimization-based center of pressure definition

This problem involves estimating two unknowns: the CoP positions along the midline of both feet. The two CoPs must be collinear with the global CoP, and the left and right CoPs are selected such that the distance between them is the shortest. A schematic representation of the problem is shown in Figure A1.

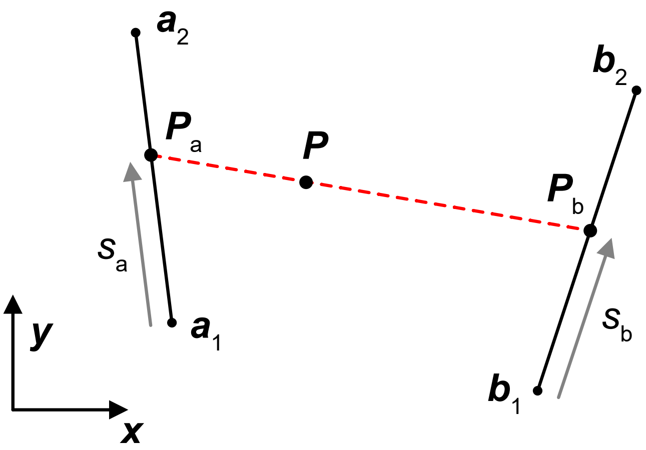

Figure A1: Schematic representation of the optimization problem. *Bold characters represent vectors. The left (a) and right (b) foot are depicted as straight black lines. The center of pressure of each foot (P_a and P_b) must be chosen so that they are collinear with the global center of pressure (P).*

To solve this problem, we defined a cost function that minimized the distance between the CoPs of each foot. This cost function was selected because it results in similar CoP locations for both feet in a symmetric posture and is independent of the coordinate system definition.

minimize $\quad L(s_a, s_b) =$

$$||(\mathbf{a}_1 + s_a * (\mathbf{a}_2 - \mathbf{a}_1)) - (\mathbf{b}_1 + s_b * (\mathbf{b}_2 - \mathbf{b}_1))||$$

s.t. $\quad \text{cross}(\mathbf{P}_a, \mathbf{P}_b) = 0$ (CoP locations collinear)

s_a in range [0, 1]

s_b in range [0, 1]

The optimization problem was solved in MATLAB using the command “fmincon” in each time step. The resulting center of pressure locations could then be used to compute the normal forces under each foot.

When the global CoP entirely crossed the midline of one foot (e.g., during gait initiation), the CoP of that foot was set to the global CoP, and the other foot’s CoP was discarded. This allowed for representative estimations even when the weight was fully shifted to one side, but results in CoP locations that do exceed the midline of the foot.

Force and moment distribution

The estimated forces acting on the feet must (i) equal the total measured force and (ii) produce zero in-plane moment around the global center of pressure (2, 3). The force for each foot was estimated by multiplying the total force by a factor, ensuring that the factors for both the left and right foot summed to one. The magnitude was inversely proportional to the ratio of the moment arms of the normal forces, which results in a net zero moment around the global center of pressure.

Following the definition of the center of pressure, only a vertical moment can exist at the global center of pressure. This vertical moment was distributed proportionally between both feet, using the same ratio as the ground reaction force. However, due to its minimal contribution, this moment was excluded from the musculoskeletal simulations. This assumption will be further validated in the section below.

Validation

The algorithm was validated using a dataset measuring the ground reaction forces in each foot with two force plates (data unpublished). Two participants performed a gait initiation task from a standing posture, walking forward over a distance of 4 m and repeating the task for 80 trials. During these trials, participants stood on two force plates for 10 seconds before beginning to walk in the forward direction. Motion capture was recorded on the heel and second metatarsal to find the midline of the foot.

Force data was down-sampled to match the motion capture recording frequency (100 Hz) and further used to compute the global center of pressure and reaction force. The global ground reaction force was calculated by summing forces from both force plates. The moments were first transformed from the force plate origin to the lab coordinate system's origin (at the top left corner of the left force plate) using the parallel axis theorem, and then summed. The global moments and normal force were used to compute the global center of pressure *P*, which is equal to the center of pressure that would be measured if only one force plate were used. The center of pressure was considered invalid in samples where the normal force was smaller than 50 N to avoid near-singular solutions.

$$\begin{bmatrix} P_x \\ P_y \\ P_z \end{bmatrix} = \begin{bmatrix} -M_y \\ M_x \\ 0 \end{bmatrix} / F_z$$

Next, the ground reaction force splitting algorithm was applied, returning new forces, moments, and centers of pressure for both feet. The error between the estimated and the measured center of pressure from each foot was quantified as the norm of their difference, as shown in Figure A2. Samples in which any marker was obstructed were omitted, usually due to a fixed seat placed behind the participant that blocked the left heel marker. Trials where any marker was obstructed for more than 30% during the first 10 seconds were entirely excluded (9 out of 160 trials).

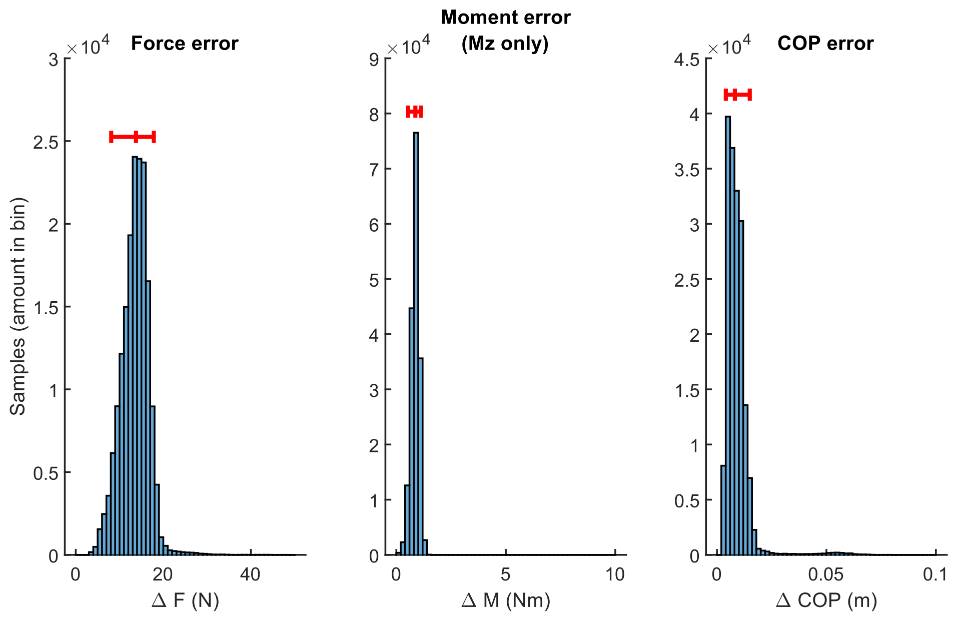

Figure A2: Histogram of force, moment, and CoP error. *Red line indicates 5-95% percentile and median.*

Overall, the median magnitude of the error between measured and estimated force was 13.7 N per foot, which is approximately 4% of the total ground reaction force per leg (336 N). This error was less than the

recommended guideline by Hicks, Uchida (4) of <5% of the force. The median error for the measured moment per foot was 0.87 Nm in the vertical direction and zero in the other directions due to the CoP being defined as the point where these moments are zero (causing in-plane errors to be zero by definition). This error was considered negligible. The estimated CoP had a median distance of 0.82 cm relative to the measurement. This error was mainly due to the CoP not being aligned with the midline of the foot, but rather being slightly more lateral in the measured CoP. The errors originating from this method fall within the desired accuracy for musculoskeletal simulations, indicating that this method could be used to estimate contributions of both legs to enable simulations.

Appendix B: Validation of trial length of indirect calorimetry measurements in Experiment 1

This appendix provides a summary of key indirect calorimetry results and evaluates whether 2-minute trials were sufficient to capture the transient response between trial conditions. Table B1 and Figure B1 report the normalized energy expenditure (as shown in Figure 2 in the manuscript), minute volume (VE), breathing frequency (Rf), and respiratory exchange ratio (RQ). The target had a significant effect on energy expenditure, VE, and Rf, but not on RQ (Table B1). This suggests that minute volume and breathing frequency varied with the effort it took to maintain a certain lean angle.

Next, we verified whether the trial length we used to evaluate cost was sufficient. The duration of preferred posture trials was 5 minutes, compared with 2 minutes in the Target condition. To verify that 2 minutes in the target trials were sufficient to reach steady-state, we ran a rmANOVA on the preferred postural trials segmented into three periods: the first minute, the second minute, and the remaining three minutes (Figure B1 and Table B1). There was no significant differences across these three time periods for any of the metrics (Table B1). We also performed the same test in the Eyes-Closed condition, and similarly found no significant effect of trial time on energy expenditure ($F_{(2,22)} = 0.493$, $p = 0.618$). Participants remained standing between trials and measurements were only started when the participant was already standing at the target, which likely contributed to a further reduction of time required to reach steady-state energy expenditure. To remain conservative in our estimates, we omitted the first minute of indirect calorimetry data to ensure that steady-state was reached. Under this approach, the results suggest that the second minute of two-minute trials was sufficient to capture the energy expenditure differences between different targets.

Table B1: Indirect calorimetry metrics from Experiment 1 across targets and of the EO condition split by time

	EE Measured<br>W/kg	*VE*<br>L/s	*Rf*<br>1/min	*RQ*<br>-
Target				
-1.15°	2.04 ± 0.68	12.34 ± 4.45	19.7 ± 4.0	0.75 ± 0.06
0°	1.70 ± 0.46	10.69 ± 3.44	17.8 ± 4.6	0.77 ± 0.06
1.15°	1.56 ± 0.46	9.87 ± 3.06	18.3 ± 5.1	0.76 ± 0.05
2.29°	1.61 ± 0.54	10.35 ± 3.31	18.1 ± 4.5	0.77 ± 0.06
3.44°	1.76 ± 0.52	10.84 ± 3.31	18.1 ± 4.0	0.76 ± 0.05
5.73°	2.06 ± 0.53	12.42 ± 3.81	20.5 ± 4.1	0.75 ± 0.07
EO	1.53 ± 0.48	9.89 ± 3.32	14.4 ± 3.7	0.80 ± 0.06
EC	1.60 ± 0.49	9.89 ± 3.43	13.8 ± 4.2	0.79 ± 0.05
Time-separated EO				
0-60s	1.55 ± 0.59	9.86 ± 3.99	14.8 ± 2.8	0.81 ± 0.08
60-120s	1.48 ± 0.48	9.56 ± 3.27	14.3 ± 3.6	0.79 ± 0.07
120-300s	1.54 ± 0.47	10.01 ± 3.33	14.4 ± 3.8	0.80 ± 0.07
rmANOVA statistics				
Target	$F_{(5,55)} = 13.83$<br>$p <$ **0.001**	$F_{(5,55)} = 6.84$<br>$p <$ **0.001**	$F_{(5,55)} = 7.59$<br>$p <$ **0.001**	$F_{(2.5,27.5)} = 0.79$<br>$p = 0.490$
Time-separated EO	$F_{(2,22)} = 1.26$<br>$p = 0.304$	$F_{(2,22)} = 0.84$<br>$p = 0.443$	$F_{(2,22)} = 0.35$<br>$p = 0.708$	$F_{(2,22)} = 0.78$<br>$p = 0.473$

Legend: EE: Energy Expenditure, VE: Minute volume, Rf: Breathing frequency, RQ: Respiratory quotient. Post hoc tests for Target rmANOVA are provided in Appendix E.

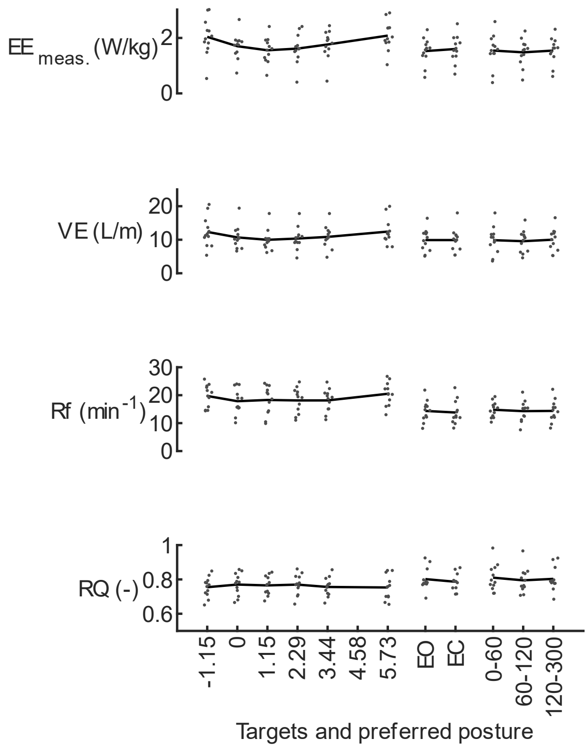

Figure B1: Supplementary indirect calorimetry data for Experiment 1. *$EE_{measured}$ is the weight-normalized energy expenditure, VE is the minute volume, Rf is the breathing frequency, and RQ is the respiratory exchange ratio. The left column depicts the participant averages and the grand average of the target and preferred posture trials. The right colum shows the data of the preferred posture (EO) trial, but separated by the first minute (0-60 s, omitted from all metabolic analyses), the second minute (60-120 s), and the remaining three minutes (120-300 s).*

Appendix C: Validation of the assumption that excitation and activation are similar in slow movements

The muscle model uses a simple first-order differential equation to describe the activation dynamics of the muscle. Excitation is the idealized neural control signal sent to a muscle, whereas activation is the normalized force generation of the muscle, which is subject to temporal activation dynamics (5, 6). The relation between the excitation and the activation of the muscle is computed as: (5, 6)

$$\dot{a}(t) = \frac{u(t) - a(t)}{\tau} \ ,$$

where $u(t)$ is the muscle excitation, $a(t)$ the muscle activation, and τ the time constant. The time constant is different for increasing or decreasing muscle activation; if $u(t)$ is larger than $a(t)$, then

$$\tau = \tau_A\big(0.5 + 1.5a(t)\big) \ ,$$

and otherwise

$$\tau = \frac{\tau_D}{0.5 + 1.5a(t)} \ ,$$

where τ_D is 40 ms and τ_A is 10 ms (5, 6). In this work, a static muscle optimizer was utilized. In static muscle optimization, the activations of all muscles are computed independently for each time step. As a result, this method does not predict muscle excitation, in contrast to dynamic methods like Musculoskeletal Optimal Control (7) or Computed Muscle Control (8).

However, the energy expenditure model by Umberger et al. (9) relies on excitation for a part of the computation. Specifically, the computation uses a factor A, which is equal to a when $u > a$, and equal to the average of u and a otherwise. Since movements in these experiments were slow, we tested whether it was reasonable always to assume that $A = a$, given that only activation was available from our static method.

Static optimization was performed at 20 Hz on movement that was low-pass filtered at 6 Hz. We assume that the activation changes linearly between samples. To determine the excitation required for a continuous rate of change in activation, the differential equation can be rewritten as

$$u(t) - a(t) = \tau \, \dot{a}(t) \, .$$

This equation indicates that the rate of change of activation is directly proportional to the error made when assuming that activation and excitation are equal for energy expenditure calculations. To validate that this error was sufficiently small, we computed the right-hand term for all muscles in all trials of Experiment 1. The time constants τ_D and τ_A were selected based on whether activation was decreasing or increasing rather than whether excitation was larger or smaller.

In all muscles and trials, the average absolute error between excitation and activation was 0.001, and the average 99th percentile error for each muscle was 0.013. This error was considered marginal for the computation of energy expenditure, suggesting that the simplification of excitation being equal to activation was acceptable. Therefore, the term $u > a$ in the Umberger model was replaced with $\dot{a} > 0$, and A was assumed equal to a, so that excitation could be omitted without ignoring the effects of

ascending/descending muscle activation in the energetic computation. This allows the use of the energy expenditure model by Umberger et al. (9) without using a dynamic method that computes muscle excitation.

Appendix D: List of motion capture markers

An *R* or *L* precedes names of markers that do not lie on the sagittal plane to indicate the left or right side of the body. The marker names follow the naming convention used by Rajagopal, Dembia (10) where possible. The weights for kinematic scaling are included in square brackets. Weights for the following inverse kinematics step were equal for all markers [50], except the XYPH marker [10] and the head markers [0, ignored].

ACR [100]: Left/Right acromion

IJ [50]: Deepest point of incisura jugularis

XYPH [1]: Xiphoid process, i.e., the most caudal point of the sternum. For some female participants (Exp 1: 3/5, Exp 2: 5/11), the intended placement of the marker was hindered by anatomy and clothing, making it invisible to the motion capture cameras. In these cases, the marker was placed midway between the navel and the xiphoid process. This marker was assigned low weight in all participants during scaling to account for the varied placement.

C7 [250]: Spinous process of the seventh cervical vertebra

T2 [25]: Second thoracic vertebrae

T7 [1]: Midpoint between the inferior angles of the most caudal points of the two scapulae. This marker was omitted in Experiment 1 due to obstruction by the back-worn metabolic analyzer.

L1, L3, L5 [25]: First, third, and fifth lumbar vertebrae

ASI [100]: Anterior superior iliac spine

PSI [50]: Posterior superior iliac spine

GTRO [25]: Most lateral prominence of the greater trochanter

LFC [50]: Most lateral prominence of the lateral femoral epicondyle

MFC [50]: Most medial prominence of the medial femoral epicondyle

FAX [50]: Proximal tip of the head of the fibula

TTC [50]: Most anterior border of the tibial tuberosity

LMAL [50]: Lateral prominence of the lateral malleolus

MMAL [50]: Most medial prominence of the medial malleolus

CAL [25]: Aspect of the Achilles tendon insertion on the calcaneus

TOE [25]: Dorsal margin of the first metatarsal head

MT2, MT5 [25]: Dorsal aspect of the second and fifth metatarsal head

LEL [50]: Humeral lateral epicondyle at the elbow.

FAulna [50]: The Most dorsal prominence of the ulnar head at the wrist.

HF, HB [1]: Markers on headband on the front and back side of the head; not used for analysis

Appendix E: Experiment 1 statistics tables

Main effect and post-hoc tests of energy expenditure (measured and simulated) were listed in Table 1. The main effects of lean angle SD and CoP velocity were reported in the main text, with post-hoc tests provided below. For metabolic analyzer metrics, the main effect was reported in Table A1 and post-hoc tests following a significant main effect are provided below.

Within-trial std of whole-body lean

Post Hoc Comparisons - Task

		Mean Difference	SE	t	p_{holm}	
-2	0	-0.00004	0.00020	-0.20010	1.00000	
	2	0.00028	0.00020	1.42258	0.80018	
	4	0.00047	0.00020	2.35769	0.19501	
	6	0.00041	0.00020	2.04443	0.31715	
	10	-0.00092	0.00020	-4.62813	0.00024	***
0	2	0.00032	0.00020	1.62268	0.65941	
	4	0.00051	0.00020	2.55779	0.13078	
	6	0.00045	0.00020	2.24453	0.22799	
	10	-0.00088	0.00020	-4.42803	0.00045	***
2	4	0.00019	0.00020	0.93511	1.00000	
	6	0.00012	0.00020	0.62185	1.00000	
	10	-0.00120	0.00020	-6.05071	1.31404e-6	***
4	6	-0.00006	0.00020	-0.31326	1.00000	
	10	-0.00139	0.00020	-6.98582	3.96718e-8	***
6	10	-0.00133	0.00020	-6.67256	1.26382e-7	***

*** $p < .001$

Note. P-value adjusted for comparing a family of 15

Center of pressure velocity

Post Hoc Comparisons - Task

		Mean Difference	SE	t	p_{holm}	
-2	0	0.00612	0.00116	5.28644	0.00002	***
	2	0.00601	0.00116	5.18982	0.00003	***
	4	0.00583	0.00116	5.03478	0.00004	***
	6	0.00553	0.00116	4.77986	0.00009	***
	10	-0.00205	0.00116	-1.76896	0.57388	
0	2	-0.00011	0.00116	-0.09662	1.00000	
	4	-0.00029	0.00116	-0.25167	1.00000	
	6	-0.00059	0.00116	-0.50658	1.00000	
	10	-0.00816	0.00116	-7.05540	3.01859e-8	***
2	4	-0.00018	0.00116	-0.15505	1.00000	
	6	-0.00047	0.00116	-0.40996	1.00000	
	10	-0.00805	0.00116	-6.95878	4.11733e-8	***
4	6	-0.00029	0.00116	-0.25491	1.00000	
	10	-0.00787	0.00116	-6.80373	7.02273e-8	***
6	10	-0.00758	0.00116	-6.54882	1.75651e-7	***

*** $p < .001$

Note. P-value adjusted for comparing a family of 15

Breathing frequency

Post Hoc Comparisons - Task

		Mean Difference	SE	t	p_{holm}	
-2	0	1.87757	0.55517	3.38197	0.01464	*
	2	1.43806	0.55517	2.59031	0.09801	
	4	1.60452	0.55517	2.89015	0.05501	
	6	1.59853	0.55517	2.87936	0.05501	
	10	-0.78226	0.55517	-1.40905	1.00000	
0	2	-0.43950	0.55517	-0.79166	1.00000	
	4	-0.27305	0.55517	-0.49182	1.00000	
	6	-0.27903	0.55517	-0.50261	1.00000	
	10	-2.65983	0.55517	-4.79102	0.00019	***
2	4	0.16646	0.55517	0.29983	1.00000	
	6	0.16047	0.55517	0.28905	1.00000	
	10	-2.22032	0.55517	-3.99936	0.00229	**
4	6	-0.00599	0.55517	-0.01079	1.00000	
	10	-2.38678	0.55517	-4.29919	0.00099	***
6	10	-2.38079	0.55517	-4.28841	0.00099	***

* $p < .05$, ** $p < .01$, *** $p < .001$

Note. P-value adjusted for comparing a family of 15

Minute volume

Post Hoc Comparisons - Task

		Mean Difference	SE	t	p_{holm}	
-2	0	1.64819	0.55963	2.94516	0.04724	*
	2	2.36779	0.55963	4.23101	0.00124	**
	4	1.99138	0.55963	3.55841	0.00933	**
	6	1.50158	0.55963	2.68317	0.07691	
	10	-0.08156	0.55963	-0.14574	1.00000	
0	2	0.71960	0.55963	1.28585	1.00000	
	4	0.34319	0.55963	0.61325	1.00000	
	6	-0.14661	0.55963	-0.26199	1.00000	
	10	-1.72976	0.55963	-3.09090	0.03442	*
2	4	-0.37641	0.55963	-0.67261	1.00000	
	6	-0.86621	0.55963	-1.54784	0.89177	
	10	-2.44936	0.55963	-4.37676	0.00081	***
4	6	-0.48981	0.55963	-0.87523	1.00000	
	10	-2.07295	0.55963	-3.70415	0.00641	**
6	10	-1.58314	0.55963	-2.82892	0.05855	

* $p < .05$, ** $p < .01$, *** $p < .001$

Note. P-value adjusted for comparing a family of 15

Appendix F: Experiment 2 statistics tables

Main effects for rmANOVA were reported in Table 2. Post-hoc tests following a significant main effect are provided below.

Time to steady-state velocity (Forward)

Post Hoc Comparisons - Target

		Mean Difference	SE	t	p_{bonf}		p_{holm}	
B	M	0.09749	0.05219	1.86806	0.20844		0.09326	
	F	0.20481	0.05219	3.92475	0.00106	**	0.00106	**
M	F	0.10733	0.05219	2.05669	0.13989		0.09326	

** p < .01

Note. P-value adjusted for comparing a family of 3

Time to steady-state velocity (Backward)

Main effect not significant.

Peak CoM velocity (Forward)

Main effect not significant.

Peak CoM velocity (Backward)

Post Hoc Comparisons - Target

		Mean Difference	SE	t	p_{bonf}		p_{holm}	
B	M	-0.01084	0.00749	-1.44682	0.46844		0.19934	
	F	-0.02349	0.00749	-3.13449	0.00994	**	0.00994	**
M	F	-0.01265	0.00749	-1.68767	0.29900		0.19934	

** p < .01

Note. P-value adjusted for comparing a family of 3

Cost of transport (Forward)

Post Hoc Comparisons - Target

		Mean Difference	SE	t	p_{bonf}		p_{holm}	
B	M	0.86176	0.22539	3.82332	0.00143	**	0.00143	**
	F	0.67179	0.22539	2.98049	0.01499	*	0.00999	**
M	F	-0.18997	0.22539	-0.84283	1.00000		0.40460	

* p < .05, ** p < .01

Note. P-value adjusted for comparing a family of 3

Cost of transport (Backward)

Post Hoc Comparisons - Target

		Mean Difference	SE	t	p_{bonf}		p_{holm}	
B	M	0.75257	0.30350	2.47966	0.05310		0.03540	*
	F	0.90647	0.30350	2.98675	0.01475	*	0.01475	*
M	F	0.15390	0.30350	0.50709	1.00000		0.61502	

* p < .05

Note. P-value adjusted for comparing a family of 3

Center of mass displacement (Forward)

Post Hoc Comparisons - Target

		Mean Difference	SE	t	p_{bonf}		p_{holm}	
B	M	0.02133	0.00596	3.57719	0.00290	**	0.00194	**
	F	0.03416	0.00596	5.72895	4.02105e-6	***	4.02105e-6	***
M	F	0.01283	0.00596	2.15176	0.11350		0.03783	*

* p < .05, ** p < .01, *** p < .001

Note. P-value adjusted for comparing a family of 3

Center of mass displacement (Backward)

Post Hoc Comparisons - Target

		Mean Difference	SE	t	p_{bonf}		p_{holm}	
B	M	-0.01425	0.00507	-2.81060	0.02332	*	0.00777	**
	F	-0.03780	0.00507	-7.45332	1.81226e-8	***	1.81226e-8	***
M	F	-0.02355	0.00507	-4.64272	0.00012	***	0.00008	***

* p < .05, ** p < .01, *** p < .001

Note. P-value adjusted for comparing a family of 3

Energy expended by muscles (Forward)

Post Hoc Comparisons - Target

		Mean Difference	SE	t	p_{bonf}		p_{holm}	
B	M	0.42454	0.05570	7.62160	1.08111e-8	***	7.20739e-9	***
	F	0.45462	0.05570	8.16166	2.09947e-9	***	2.09947e-9	***
M	F	0.03008	0.05570	0.54007	1.00000		0.59230	

Post Hoc Comparisons - Target

		Mean Difference	SE	t	p_{bonf}		p_{holm}	

*** p < .001

Note. P-value adjusted for comparing a family of 3

Energy expended by muscles (Backward)

Post Hoc Comparisons - Target

		Mean Difference	SE	t	p_{bonf}		p_{holm}	
B	M	0.03729	0.04281	0.87098	1.00000		0.38923	
	F	-0.14997	0.04281	-3.50292	0.00359	**	0.00239	**
M	F	-0.18726	0.04281	-4.37391	0.00028	***	0.00028	***

** p < .01, *** p < .001

Note. P-value adjusted for comparing a family of 3

References

1. Sloot LH, Millard M, Werner C, Mombaur K. Slow but Steady: Similar Sit-to-Stand Balance at Seat-Off in Older vs. Younger Adults. Front Sports Act Living. 2020;2:548174.
2. Meurisse GM, Dierick F, Schepens B, Bastien GJ. Determination of the vertical ground reaction forces acting upon individual limbs during healthy and clinical gait. Gait Posture. 2016;43:245-50.
3. Davis BL, Cavanagh PR. Decomposition of superimposed ground reaction forces into left and right force profiles. J Biomech. 1993;26(4-5):593-7.
4. Hicks JL, Uchida TK, Seth A, Rajagopal A, Delp SL. Is my model good enough? Best practices for verification and validation of musculoskeletal models and simulations of movement. J Biomech Eng. 2015;137(2):020905.
5. Uchida TK, Delp SL, Delp D. Biomechanics of Movement: The Science of Sports, Robotics, and Rehabilitation: MIT Press; 2021.
6. Suzuki Y, Geyer H. A Neuro-Musculo-Skeletal Model of Human Standing Combining Muscle-Reflex Control and Virtual Model Control. Annu Int Conf IEEE Eng Med Biol Soc. 2018;2018:5590-3.
7. Dembia CL, Bianco NA, Falisse A, Hicks JL, Delp SL. OpenSim Moco: Musculoskeletal optimal control. PLoS Comput Biol. 2020;16(12):e1008493.
8. Thelen DG, Anderson FC, Delp SL. Generating dynamic simulations of movement using computed muscle control. J Biomech. 2003;36(3):321-8.
9. Umberger BR, Gerritsen KGM, Martin PE. A Model of Human Muscle Energy Expenditure. Computer Methods in Biomechanics and Biomedical Engineering. 2003;6(2):99-111.
10. Rajagopal A, Dembia CL, DeMers MS, Delp DD, Hicks JL, Delp SL. Full-Body Musculoskeletal Model for Muscle-Driven Simulation of Human Gait. IEEE Trans Biomed Eng. 2016;63(10):2068-79.
11. JASP Team. JASP (Version 0.18.1) [Computer software]. 2023.
