# Supplementary material for: The energetic cost of human standing balance and gait initiation over a range of natural postures
Source: PLoS Comput Biol. 2026 Jul 7;22(7):e1013522. doi: 10.1371/journal.pcbi.1013522 (PMC13340847; doi:10.1371/journal.pcbi.1013522)

Experiment 1 simulated muscle activation for all conditions and all muscles

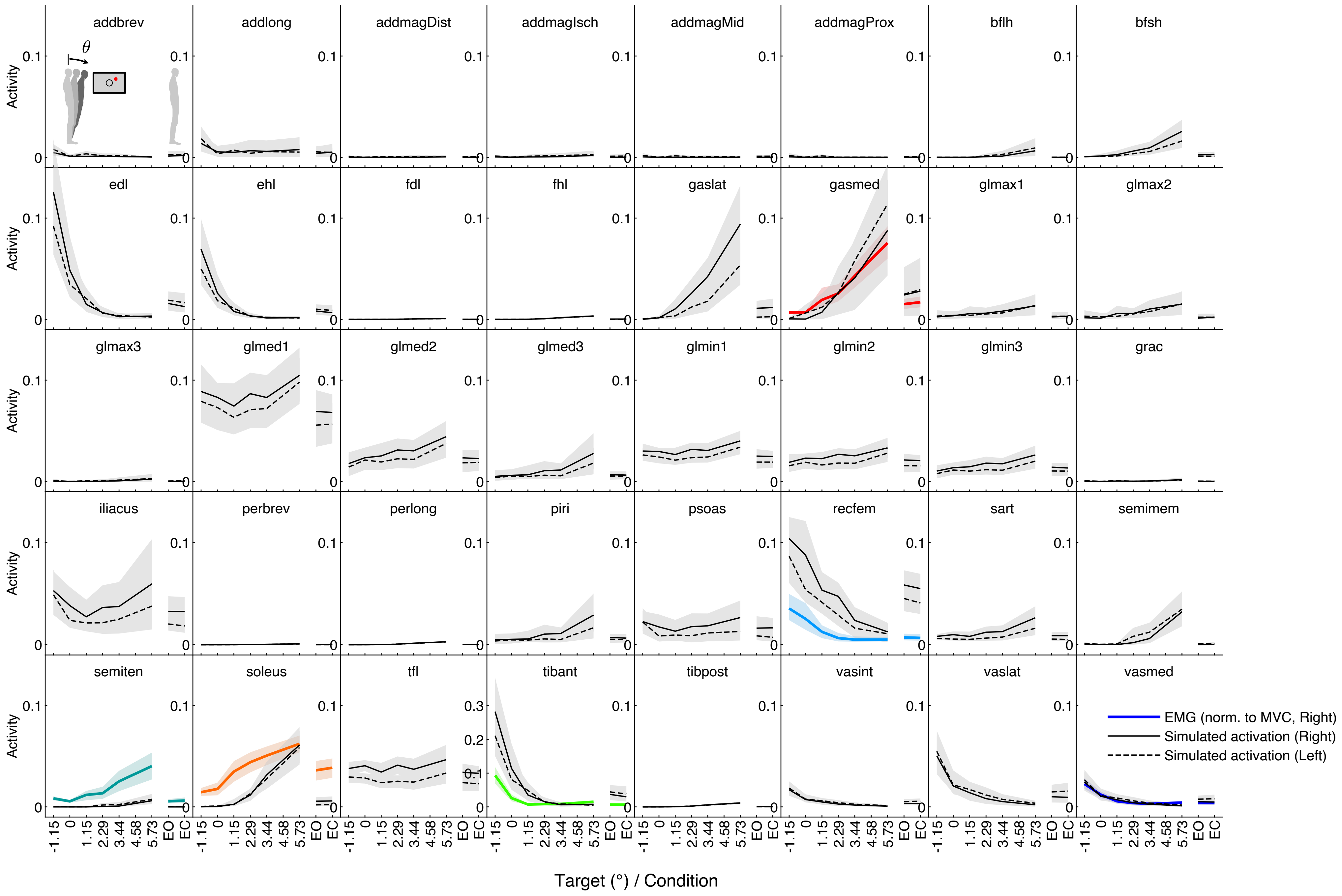

Supplement: S1 Fig — Figure showing the simulated muscle activations of all 80 muscles during the target and preferred posture trials of Experiment 1, compared against EMG data where available. Labels correspond to muscle name abbreviations used in the Rajagopal, Dembia [13] musculoskeletal model. (PDF) [file pcbi.1013522.s001.pdf]
