# Supplementary material for: The energetic cost of human standing balance and gait initiation over a range of natural postures
Source: PLoS Comput Biol. 2026 Jul 7;22(7):e1013522. doi: 10.1371/journal.pcbi.1013522 (PMC13340847; doi:10.1371/journal.pcbi.1013522)

Experiment 2 simulated muscle activation for all muscles during **Backward** gait initiation from prescribed targets

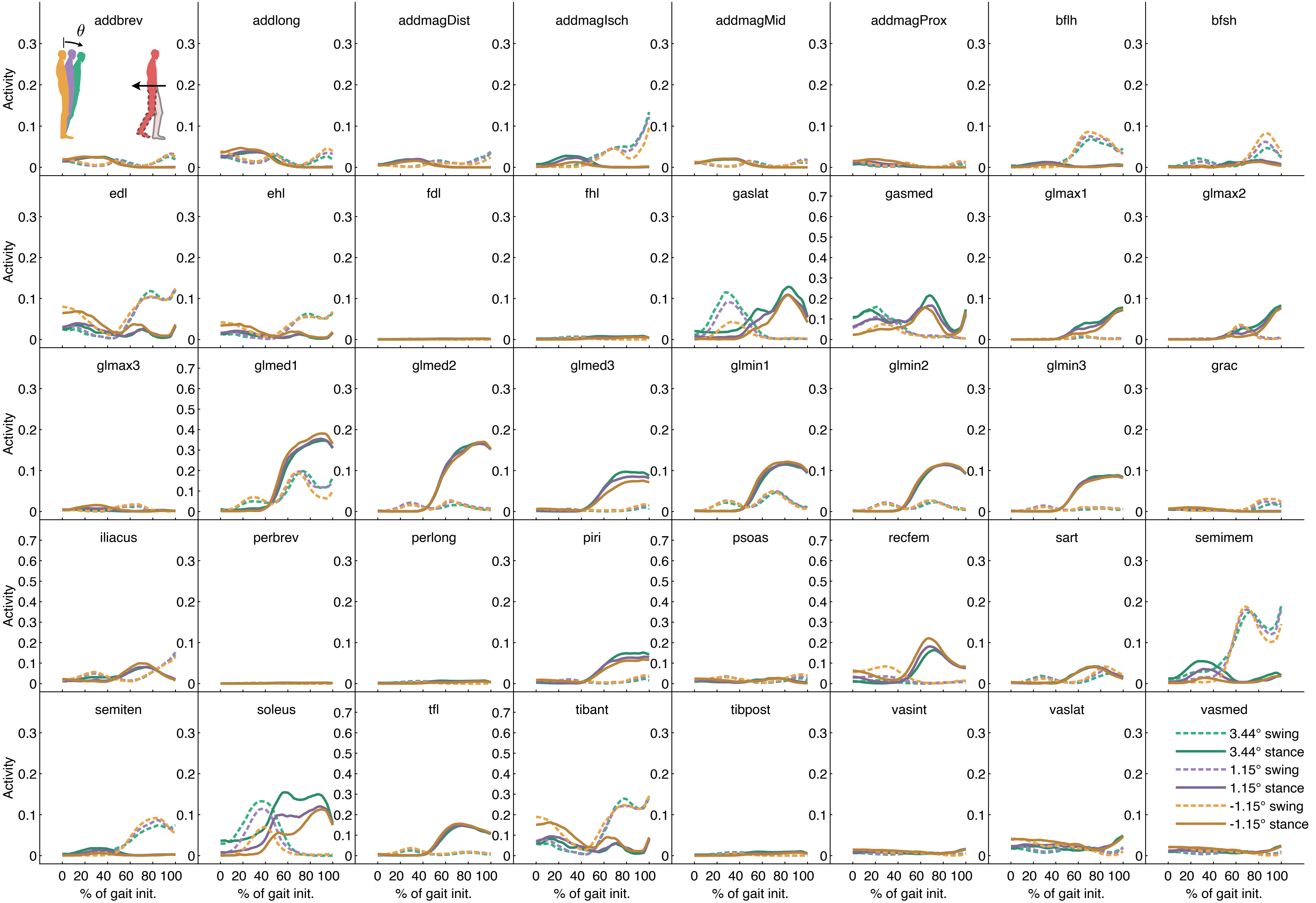

Supplement: S3 Fig — Figure showing the simulated muscle activations of all 80 muscles during backward gait initiation, split between the stance and swing leg, and for each starting lean angle. (PDF) [file pcbi.1013522.s003.pdf]
