# Supplementary material for: The energetic cost of human standing balance and gait initiation over a range of natural postures
Source: PLoS Comput Biol. 2026 Jul 7;22(7):e1013522. doi: 10.1371/journal.pcbi.1013522 (PMC13340847; doi:10.1371/journal.pcbi.1013522)
Supplement: Attachment — Submitted filename: postural_control_2026.pdf [file pcbi.1013522.s006.pdf]

This manuscript addresses (i) how metabolic cost varies with standing posture, (ii) how the cost of gait initiation depends on posture, and (iii) whether humans adopt postures that minimize cost during quiet standing and in preparation for walking. These are interesting and relevant research questions, and the work has the potential to make a valuable contribution to the field. The study appears to be based on a substantial amount of experimental work, and the general approach is methodologically sound.

However, in its current form the manuscript requires major revisions before it can be considered for publication. It would benefit from substantial restructuring to improve readability and transparency. Overall, the manuscript is very dense and contains many sentences that repeat similar information. I would strongly recommend major rewording and editing to reduce the length and improve the clarity of the text.

My detailed comments and suggestions are provided below and are intended to help the authors improve the quality and clarity of the manuscript.

1 – Results

I have a major suggestion regarding the presentation of the results. Because the study includes two experiments and multiple outcome metrics, the current presentation is difficult to follow.

First, I recommend choosing a single unit for angular measures (either radians or degrees). Reporting both units throughout the results makes the manuscript unnecessarily confusing.

Second, many of the numerical results could be presented more effectively in tables rather than repeatedly embedded in the text. This would substantially improve the readability of what is currently a very dense Results section.

In addition, the reporting of p-values is inconsistent. Sometimes values are reported as "p < …" and sometimes as "p = …", even for significant results. I suggest adopting a uniform reporting style throughout the manuscript (for example, using "p = …" and reserving "p < .001" only when appropriate). Consistency would improve the clarity and professionalism of the presentation.

2 – Methods and statistical analysis

The methods and statistical analyses are generally understandable, but this section is also quite dense and repetitive. It could be streamlined substantially.

Several sentences list many outcome variables in long chains that could be simplified.

Some methodological details are explained multiple times (e.g., use of Holm correction, paired-samples t-tests).

The rationale for separating forward and backward analyses is reasonable, but the explanation is somewhat wordy and could be condensed.

3 – Discussion

In several places, the Discussion includes detailed numerical results. I find that repeating exact numerical values in the Discussion tends to overwhelm the reader and is unnecessary. I would recommend removing most of these numbers and focusing on interpretation instead.

When discussing control strategies and the simulation results, it would be valuable to acknowledge related work in this area. In particular, the authors may wish to cite studies by K. Shiozawa, J. Lee, M. Russo, D. Sternad, and N. Hogan (e.g., Journal of NeuroEngineering and Rehabilitation, 2021; Journal of Neurophysiology, 2024), which address similar issues and would help place the current findings in a broader context.

Minor comments

Lines 77–86: I would avoid foreshadowing the results and the significance of the paper in the Introduction. This section should focus on background and rationale rather than anticipated outcomes.

Lines 100–101: Although the protocol is relatively simple, I suggest providing a more complete description here so that the Methods section is fully self-contained.

Line 200: I recommend toning down this statement. As the authors themselves note, and as is visible in Figure 3, the model only partially captures the EMG activity. The claim should therefore be expressed more cautiously.
