# Supplementary material for: The energetic cost of human standing balance and gait initiation over a range of natural postures
Source: PLoS Comput Biol. 2026 Jul 7;22(7):e1013522. doi: 10.1371/journal.pcbi.1013522 (PMC13340847; doi:10.1371/journal.pcbi.1013522)
Supplement: Attachment — Submitted filename: R1 - Rebuttal.pdf [file pcbi.1013522.s008.pdf]

Dr. Patrick A. Forbes
Department of Neuroscience
Erasmus MC, University Medical Center Rotterdam
Rotterdam, The Netherlands
email: p.forbes@erasmusmc.nl

Dear PLOS Computational Biology Editorial Board,

Please find enclosed our revised manuscript entitled "*The energetic cost of human standing balance and gait initiation over a range of natural postures*" for your consideration.

We thank the reviewers for their highly valuable and thorough evaluations of our work. Below, we address all comments and highlight the changes made to the manuscript. To briefly summarize, the following major changes were made:

1. Restructuring of the manuscript to improve clarity, reduce redundancy, and better separate the different sections,
2. Rewrite of the Results to improve readability by moving statistics and numeric values into new Tables 1 and 2, and including significance bars in figures,
3. Rewrite of the Statistics section to improve clarity,
4. Rewrite of the Author summary to be more distinct from the Abstract,
5. Create Supplementary Figures S1-S3 to provide a complete overview of all simulated muscles.

We believe that these revisions have strengthened the manuscript considerably and have addressed the concerns raised during review. We appreciate the consideration of our manuscript for publication in *PLOS Computational Biology* and look forward to your response.

Sincerely,

Dr. Patrick A. Forbes

On behalf of all authors

Response to reviewers and editor

Below we list the original reviewer and editor comments (black) and our responses (blue). Reference numbers are tracked separately in the rebuttal letter and manuscript. We have included all of Reviewer 1's comments from the PDF and identified the corresponding lines and sentences in the original submission.

Additional Editor Comments:

This study is of high quality and relevant to biomechanical field. However, some issues must be addressed before further consideration of the manuscript, particularly with respect to the completeness of the literature review, manuscript structure, and the consistency of the musculoskeletal model. Additionally, the authors are required to update the provided data/code DOI, which is currently not accessible.
We encourage the authors to carefully address all the comments from reviewers and to provide a detailed, point-by-point response.

We thank the editor for their feedback on the manuscript. We have now created a DOI on DataverseNL (Leeuwis et al., 2026) with link https://doi.org/10.34894/RAWEHM, which is now included in the data availability statement:

> *Data availability statement*
>
> *All data and code supporting the findings of this study are publicly available in "Data and code for 'The energetic cost of human standing balance and gait initiation over a range of natural postures'",* ***https://doi.org/10.34894/RAWEHM, DataverseNL (123)****.*

Journal Requirements:

If the reviewer comments include a recommendation to cite specific previously published works, please review and evaluate these publications to determine whether they are relevant and should be cited. There is no requirement to cite these works unless the editor has indicated otherwise.

1) Please ensure that the CRediT author contributions listed for every co-author are completed accurately and in full.
At this stage, the following Authors/Authors require contributions: Matto Leeuwis. Please ensure that the full contributions of each author are acknowledged in the "Add/Edit/Remove Authors" section of our submission form.
The list of CRediT author contributions may be found here: https://journals.plos.org/ploscompbiol/s/authorship#loc-author-contributions

We apologize for the missing author contributions and have corrected them in the editorial manager.

2) We ask that a manuscript source file is provided at Revision. Please upload your manuscript file as a .doc, .docx, .rtf or .tex. If you are providing a .tex file, please upload it under the item type u2018LaTeX Source Fileu2019 and leave your .pdf version as the item type u2018Manuscriptu2019.

The manuscript source file has been provided as a .docx file.

3) Please upload all main figures as separate Figure files in .tif or .eps format. For more information about how to convert and format your figure files please see our guidelines: https://journals.plos.org/ploscompbiol/s/figures

All figures have been provided in .eps format and uploaded to the editorial manager.

4) Some material included in your submission may be copyrighted. According to PLOSu2019s copyright policy, authors who use figures or other material (e.g., graphics, clipart, maps) from another author or copyright holder must demonstrate or obtain permission to publish this material under the Creative Commons Attribution 4.0 International (CC BY 4.0) License used by PLOS journals. Please closely review the details of PLOSu2019s copyright requirements here: PLOS Licenses and Copyright. If you need to request permissions from a copyright holder, you may use PLOS's Copyright Content Permission form.
Please respond directly to this email and provide any known details concerning your material's license terms and permissions required for reuse, even if you have not yet obtained copyright permissions or are unsure of your material's copyright compatibility. Once you have responded and addressed all other outstanding technical requirements, you may resubmit your manuscript within Editorial Manager.
Potential Copyright Issues:
i) Please confirm (a) that you are the photographer of 1A, or (b) provide written permission from the photographer to publish the photo(s) under our CC BY 4.0 license.

The authors confirm that we are the photographers of the photo used in Figure 1A.

ii) Figures 1, 3, and 4A. Please confirm whether you drew the images / clip-art within the figure panels by hand. If you did not draw the images, please provide (a) a link to the source of the images or icons and their license / terms of use; or (b) written permission from the copyright holder to publish the images or icons under our CC BY 4.0 license. Alternatively, you may replace the images with open source

alternatives. See these open source resources you may use to replace images / clip-art:
- https://commons.wikimedia.org
- https://openclipart.org/.

The authors confirm that we created all clip-art in all figures for the purpose of this study. The depictions of the musculoskeletal model were generated and modified by the authors. The original musculoskeletal model Rajagopal et al. (2016) and the software (OpenSim, Seth et al. (2018)) are available under a CC BY license and are accredited in the figure caption.

5) Please note that your Data Availability Statement is currently missing the DOI/accession number of each dataset OR a direct link to access each dataset. If your manuscript is accepted for publication, you will be asked to provide these details on a very short timeline. We therefore suggest that you provide this information now, though we will not hold up the peer review process if you are unable.

We have created a DOI on Dataverse with link https://doi.org/10.34894/RAWEHM for our dataset (Leeuwis et al., 2026), which is included in the data availability statement (see comment above).

Reviewers' comments:

Reviewer's Responses to Questions.

Reviewer #1:

Dear Authors,

Your work presents a study on the energy consumption mechanisms during unperturbed quite stance with respect to the whole-body orientation.

The work is well written, novel, and of high interest to the biomechanics and human neuromotor control community.

I honestly appreciated the rigor and quality with which you wrote your manuscript.

You will be able to find all my comments, questions, and recommendations in the attached PDF file in the form of highlighted portions of text with annexed comments.

As general comments, I think your work would benefit from 2 major improvements:
- Literature Review: I provided multiple references that I believe are highly relevant to the presented work and would enrich the introduction and discussion sections of your manuscript.
- Figure presentation: In the version I was given access, most figures had a very low resolution which reduced the overall quality of the figure and possibility to appreciate the details of the presented data. I recommend switching from images to vector graphics, if possible.

Please address all of my comments here and in the attached PDF.

I very much look forward to reading the final version of this manuscript.

Kind regards,

The Reviewer

We thank the reviewer for their thoughtful assessment and detailed comments in the PDF, which greatly helped us improve the quality of our manuscript. Please find our comment-by-comment responses below.

We have paraphrased the original manuscript (including original line numbers) to which the comments were attached, and have grouped together related comments for clarity.

Reviewer #1 PDF comments:

Line 1: "*Humans typically select movements that minimize energetic cost,*"

MAJOR: While I agree that this statement is true in several human movements, I would argue against opening your abstract with it - since it is very easy to list movements in which energetic cost is not minimized. For example, in athletic movements the objective function might not be energetic efficiency, but maybe accuracy (throwing a dart), or output power (a boxer hook), or exerted force (javelin throw).

I suggest slightly revising the sentence, to emphasize that energetic optimization/minimization has been found in several human movements.

"Energetic cost miminization has been found in several human movements, with particular evidence during locomotion".

We agree with the reviewer that the original comment may too strongly suggest that energy, or even optimality, is the dominant factor in all movements. We have revised our claims of optimality to reflect that energy is not the only cost function shaping this movement. This is particularly relevant for the interpretation of Experiment 2, where we hypothesized that energy and time could form a tradeoff that leads to our preferred posture. In relation to the sentence highlighted by the reviewer, we have updated the abstract as follows.

> *Abstract (line 1)*
>
> ***Human movement control is shaped by competing objectives, among which minimizing energy expenditure plays a central role, particularly in determining preferred walking patterns.***

Line 21: "*Humans are thought to move in ways that save energy*."

Same here, I agree, but I recommend to soften the claim by stating that this is true for selected types of movements or tasks.

We have rewritten parts of the author summary to make it more distinct from the abstract. In this rewriting, the opening statement on moving in ways that save energy was no longer needed.

> *Author summary (line 21)*
>
> ***In this study, we investigated whether energy expenditure helps explain how humans select their standing posture. We quantified the energetic cost of standing at various lean angles using indirect calorimetry and musculoskeletal simulations, and found that participants stood close to the lowest-cost angle.***

Line 33: "Human movement control is shaped by the optimization of competing cost functions that account for the efficiency, accuracy, and energy expenditure required to achieve a desired movement goal."

I warmly invite authors to revise also alternative hypotheses to the 'optimal' control one.

Humans can (and do sometimes) move according to optimal criteria. However, generalizing this to any human movement might be wrong (and a dangerous assumption).

2 Good reads on the topic might be:

- Loeb GE. Optimal isn't good enough. Biol Cybern. 2012 Dec;106(11-12):757-65. doi: 10.1007/s00422-012-0514-6. Epub 2012 Aug 16. PMID: 22895830.

- F. Tessari,A.M. West, & N. Hogan, Explaining human motor coordination via the synergy expansion hypothesis, Proc. Natl. Acad. Sci. U.S.A. 122 (13) e2501705122, https://doi.org/10.1073/pnas.2501705122 (2025).

We appreciate the alternative viewpoints of Loeb (2012) and Tessari et al. (2025), and agree that (postural) movement control may not necessarily be implemented through an optimal control mechanism. We realize that the statement highlighted by the reviewer may steer some readers to the idea that this is the direction taken in the current study. In the Introduction, we have revised our opening statement so as not to commit to optimal control as the main mechanism for movement control.

> *Introduction (line 33)*
>
> *Human movement control* ***must balance a variety of competing objectives, such as speed, accuracy, and energy expenditure. During walking, for example, humans tend to use gait patterns (e.g., cadence and speed) that minimize energetic cost per distance traveled (1-11), but trade efficiency for other objectives when the goal demands it (e.g., speed during competition).***

In the discussion, we have explicitly provided alternatives for mechanisms that could lead humans to select optimized postures. Both suggested references proved useful here.

> *Discussion - Preferred human posture aligns with the energetic minimum (line 348)*
>
> ***Thus, while our findings suggest that humans preferentially adopt near-optimal postures from an energetic perspective, the control mechanisms underlying this selection remain unresolved. Possible explanations include optimal control theory (66), synergy- or repertoire-based learning (67, 68), or even evolved or learned priors that bias behavior towards sufficiently optimized solutions (69, 70).***

Line 39: "…, it is compelling to propose that humans also minimize energetic cost while standing."

MINOR: Word choice. I would rather say: "it is interesting to evaluate whether humans also minimize energetic cost while standing."

We have adopted a modified version of the wording suggested by the reviewer.

> *Introduction (line 38)*
>
> *Given the evidence of energy optimization in locomotion, it* ***is important to investigate whether*** *humans also minimize energetic cost while standing.*

Line 41: "single-link inverted pendulum actuated by a single moment around the ankle."

True, but consider that newer studies have brought evidence that the single-inverted pendulum model cannot capture some aspect of quiet stance.

For reference, see:

Shiozawa, K., Lee, J., Russo, M., Sternad, D., &; Hogan, N. (2021). Frequency-dependent force direction elucidates neural control of balance. Journal of NeuroEngineering and Rehabilitation

Line 429: "(e.g., a linear quadratic regulator minimizing control action and kinematic state)"

See previously recommended works by Shiozawa et al.

We thank the reviewer, as well as Reviewer 3, for sharing this insightful work by Shiozawa et al. (2021). In the section of our original manuscript highlighted by the reviewer, the inverted pendulum comparison is used to illustrate that previous studies assume kinetic inputs (joint moments) directly translate into a control metric representing effort. While this argument is clearest in single-link inverted pendulum models, the assumption applies to multi-link models as well. Therefore, we have expanded the statement about the inverted pendulum to apply to multi-link models as well.

> *Introduction (line 39)*
>
> *Models of quiet standing often represent postural control as a* ***single- or multi-link*** *inverted pendulum actuated by joint moments (**17-30**). In these simplified representations, the moments required to maintain a static orientation scale linearly with the ankle angle and are minimal when standing straight upright. Accordingly, models of postural control are commonly formulated as linear controllers (e.g., proportional-derivative control or linear quadratic regulator) that use the effort (**i.e., joint moment; 21, 24, 28, 30**) and/or deviations from a single reference angle or posture (21-27, **30**) as cost functions.*

Addressing the reviewer's comment, we agree that the work by Shiozawa et al. (2021) shows that inverted pendulum models may not fully capture all characteristics of standing. We have added a statement in the discussion to highlight this point. We have also used the citations highlighted by the reviewer to strengthen arguments elsewhere in our Discussion.

> *Discussion – Towards improved models of cost in posture (lines 416 & 409)*
>
> ***More broadly, it is also important to consider whether a model's mechanical representation may limit inferences about energy expenditure. A single-link inverted model cannot capture multi-segment coordination and may not accurately represent higher-frequency dynamics (30, 84), possibly underestimating the control effort and highlighting the need to select appropriate model mechanics and cost functions for the problem at hand.***
>
> *…*
>
> *Future work could evaluate whether multi-objective control models (e.g., a linear quadratic regulator minimizing control action and kinematic state (21, 24, 28, **30**)) and intermittent control (80-82) can be improved by incorporating asymmetric weights to control actions in the anterior and posterior directions, which may better represent the cost of accelerating the body in these directions.*

Line 63: "Optimal control in quiet standing could represent a compromise between the cost of static standing and the posture-dependent cost of gait initiation, acting under the constraint of time."

This is an intriguing idea, however - playing devil's advocate - this might be through also for standing on 4 limbs as it happens in most quadruped mammals (cats, dogs, cattles, horses, etc.).

Have you looked at the evolutionary origin of humans' bipedal stance? There might be something interesting there - though I honestly don't know much about it.

We thank the reviewer for this intriguing idea, which has been the topic of discussion in some of our lab meetings. Below we first offer some exploratory thoughts on quadrupedal standing, and then discuss how we revised the manuscript in response to this comment.

One proposed advantage of human bipedalism is a reduction in the energetic cost of transport (Rodman & McHenry, 1980). At the same time, bipedal standing involves a relatively small base of support, which is impactful not only for balance (because the margins for error are small), but also for gait initiation (because it limits the moment arm available to accelerate the body forward). Quadrupedal animals' center of pressure location is far less constrained by the size of their base of support, which may allow them to achieve higher acceleration from a resting posture without having to make simultaneous postural

adjustments. This suggests that the trade-off between energetic efficiency of static posture and movement readiness may be stronger in bipeds than in quadrupeds. Although speculative, this framing is consistent with our broader premise that standing posture may reflect a compromise between energetic efficiency and movement readiness, and it suggests an interesting direction for future comparative work between mammalian species.

Returning to the manuscript, we have clarified that the tradeoff we propose is specifically intended to reflect bipedal standing, and feel that a fuller discussion of quadrupedal standing, while interesting, may be beyond the intended scope of the current work.

> *Introduction (line 63)*
>
> *Optimal control in quiet **bipedal** standing could represent a compromise between the cost of static standing and the cost of gait initiation **from that posture**, under the constraint of movement onset time.*

Line 91: “of 8 muscles”

MINOR: Maybe list also the muscles in the caption, it might save reader’s time.

The measured muscles have been included in the caption of Figure 1.

> *Figure 1 Caption (line 116)*
>
> *The EMG amplifier recorded EMG signals of 8 muscles on the right leg **(medial gastrocnemius, soleus, tibialis anterior, vastus medialis, rectus femoris, biceps femoris, semitendinosus, and erector spinae)**.*

Line 99: “Target trials; whole-body lean angles were prescribed by asking participants to maintain the CoP cursor in a target, which was estimated as the projection of the CoM on the ground plane at the given angle.”

I'm a little concerned by this approach. Humans CoP drifts/roams during quiet stance, and it drifts following an almost pure Brownian process behavior i.e., a random walk.

If you were forcing subjects to maintain their CoP in a certain target position - depending on the size of the target - this might have disrupted the natural quiet stance behavior.

Please refer to these work, for a general overview of human quiet stance postural sway:

- Collins JJ, De Luca CJ. Open-loop and closed-loop control of posture: a random-walk analysis of center-of-pressure trajectories. Exp Brain Res. 1993;95(2):308-18. doi: 10.1007/BF00229788. PMID: 8224055.

- Tessari, F., Hermus, J., Sugimoto-Dimitrova, R. et al. Brownian processes in human motor control support descending neural velocity commands. Sci Rep 14, 8341 (2024). https://doi.org/10.1038/s41598-024-58380-5

Can you please comment on this aspect?

Line 172: “The within-trial standard deviation of lean angle was roughly double that of the minimum-cost target (EO: 0.92 ± 0.32 [illegible] 10-2 rad (0.49 ± 0.18°), Target 2: 0.38 ± 0.08 [illegible] 10-2 rad (0.22 ± 0.05°), t = 5.774, pHolm < 0.001, Figure 2B), likely because the task to remain within the target constrained the allowed orientations.”

These results support one of my previous comments i.e., subjects were not 'free' to 'drift around' as they would normally do in quiet stance. You can immediately see that in Figure 2B second panel.

The lean angle standard deviation was much smaller in the targeted postures than in the preferred configuration with EO.

Can we then talk of quiet unconstrained stance in your experiment? What are the motor control consequences of this constraint?

Please revise the literature I proposed before, and integrate it in your discussion.

Line 528: "The cursor turned green when it was within the target area,"

How big was the target area compared to the physiological postural sway are?

By looking at your results, I suspect that the target area was smaller than the typical area of postural sway.

If so, that makes your target position more constraining that physiological upright posture.

While this does not invalidate your results, I would clearly explain it in the Discussion and here, so that readers are aware of the constraint.

The reviewer raises an important point in the three related comments above, and we have reviewed the work by Collins and De Luca (1993) and Tessari et al. (2024) to address these concerns. It is clear that natural standing has substantial sway, including low-frequency drifts, and that imposing a target alters this behavior. In designing the study, we had to balance two competing aims: preserving natural postural sway patterns and maintaining experimental control over lean angle to quantify energetic costs across a defined range of postures.

Indirect calorimetry required participants to remain at a certain lean angle, necessitating a restriction on allowed postural orientations to get consistent results. From previous work, such as the suggested literature, we knew that not constraining posture would lead participants to drift away from their starting location, invalidating the energy expenditure estimate. We therefore used a target (radius = 0.3°, indicated by horizontal line in lean angle SD panel in Figure 2B) that ensured participants remained near the prescribed lean angle throughout the trial. This target reduced drift in lean angle as intended (Figure 2B). We have explicitly included the reasoning behind this decision in the Methods:

> *Materials and methods - Experiment 1: Static standing protocol (lines 515 & 525)*
>
> *In target trials, participants were instructed to match their CoP to a visual target displayed on a screen 3.20 m in front of them, at a height of 1.67 m.* ***This target was necessary to limit natural drifts in quiet standing posture (76-79), which would invalidate the indirect calorimetry assessment of a specific lean angle.***
>
> *...*
>
> *The radius r_{target} of the target circle was normalized to body length so that a similar whole-body angle excursion would result in a similar deviation from the target for all participants.* ***The target radius is shown in Figure 2B to allow direct comparison with the natural within-trial variability of lean angle, indicating whether participants successfully restricted their spontaneous sway to the target range.***

As for the implications, previous work by Houdijk et al. (2015) indicated that there are increased energetic costs to strictly controlling CoP movement, though we caution that participants in their study stood on foam to increase the difficulty of maintaining balance. In our study, the target significantly reduced lean angle SD, as intended, but did not increase CoP velocity or energy expenditure at the minimum-cost target compared with the preferred posture (Figure 2). This suggests that although the target constrained the natural drift of natural standing by design, the overall control was not substantially altered. We have included this point in the discussion and incorporated the suggested work by Collins and De Luca (1993) and Tessari et al. (2024).

> *Discussion - Variability and flexibility in postural control (line 389)*

> *Furthermore, when standing at preferred postures, the within-trial standard deviation of lean angle* ***was roughly twice that of*** *the minimum-cost target (Figure 2B*, ***Table 1****), consistent with larger postural variability and low-frequency sway previously reported in unconstrained standing (76-79). This greater dispersion of lean angles did not coincide with higher CoP velocity or metabolic cost* ***(Figure 2), suggesting that the target successfully constrained the natural sway without substantially increasing the control demand. Strictly limiting the permitted CoM or CoP variability can increase energetic expenditure due to the higher incidence and magnitude of postural corrections (28, 39), though energetic costs also rise when variability is artificially increased using more challenging experimental conditions (38).*** *Intermittent balance control models (80-82) formalize this tradeoff between effort and variability by permitting postural sway until a stability threshold is exceeded, thereby reducing energetic demands compared to continuous control (83). Overall, minimizing variability does not appear to be a prerequisite for minimizing energy expenditure, suggesting that efficient postural control does not require constraining posture at a single energetic minimum.* ***Furthermore, our findings suggest that interpretations of postural control without a fixed reference angle, such as intermittent control (80-82) or random walk (i.e., Brownian motion, (77-79)), are not at odds with energetically optimized standing as long as posture remains within the central region of measured costs (Figure 2).*** *The implications of these observations on modeling methods are discussed further below.*

Lastly, we included a statement in our discussion on the relevance of balance control models without a fixed reference angle to simulating energetically efficient standing.

> *Discussion - Towards improved models of cost in posture (line 414)*
>
> ***Furthermore, our work suggests that energy expenditure in posture is sufficiently optimized by limiting sway within a central region of lower-cost lean angles, providing experimental support for balance interpretations that do not rely on a reference angle to optimize control (e.g., intermittent control (80-82) or Brownian motion (77-79)).***

Line 154: "Figure 2"

MINOR: The figure is well organized and understandable. However, the image resolution is very poor. Could you increase it or change the image to vector graphic?

Line 278: "Figure 4"

Again, very clear and well explained figure, but the quality of the image is particularly low. Can you fix this?

We apologize for the image quality in the merged PDF; the figures were embedded as SVGs in the submitted PDF and may have been compressed during merging. We have uploaded the vector image files in .TIF format to ensure optimal image quality.

Line 184: "This suggests that minimizing whole-body variability, a common objective in postural control models, may not be a prerequisite for minimizing energy expenditure."

I agree with this statement, and there is actually some literature supporting your claim.

Going back to the point and using more colloquial terms, it seems that humans like (or don't care much) about their natural postural sway (as you can clearly see from Figure 2B second panel during preferred posture EO and EC) - and this doesn't affect performance that much.

Consider commenting on this in the discussion, and integrate with existing literature.

We agree with the reviewer and included a brief interpretation of Brownian motion/postural drift in the context of energy expenditure. The changes can be found in our reply to the comments on original **Lines 99, 172, 528** above.

Line 225: "This confirms that static standing primarily expends energy through sustained low-level muscle activation, rather than dynamic contraction or external work."

I agree, and find this intuitively reasonable. I think your results provide quantitative support to an often neglected aspect of quiet stance.

We thank the reviewer for their kind words and kept this statement as-is.

Line 232: "This result suggests that posterior leaning may recruit less metabolically efficient muscles for prolonged activation maintenance, contributing to the higher energetic cost observed when leaning in that direction."

Could this also partially explain while older adults tend to lean forward with age? Is that a more stable / less tiring configuration?

The reviewer makes an interesting observation. Given that healthy young adults already select preferred lean angles near the energetic minimum (shown both here and in previous studies), it is not clear that leaning forward would provide an energetic benefit as humans age. We note though that it may be possible that the shape of the energetic cost curve may differ with age, which could contribute to specific behaviors that change with aging. We remain cautious with this interpretation because our study is unable to provide insight into the effects of age. Therefore, we have included a recommendation to assess changes in the distribution of energetic costs with age for future work, with a reference to Horak (2006) and Manini (2010).

> *Discussion - Towards improved models of cost in posture (line 436)*
>
> *Future work could investigate how stabilization of the spine and upper body, individual (sex) differences in morphology and muscle strength (88, 89), weight distribution,* ***physiological and behavioral changes with ageing (61, 90)****, and passive ankle stiffness (46, 72, 91) affect energy expenditure and optimal posture.*

Line 248: "Participants (N=20)"

Is this a new complete set of participants or some of the subjects are shared between the 2 experiments?

One participant completed both experiments; the others are unique to each experiment. This information was added to the Methods.

> *Materials and Methods – Participants (line 454)*
>
> *A total of 33 healthy adult participants aged 18-50 with no history of balance, neurological or musculoskeletal disorders were recruited for this study (Experiment 1: 8 male, 5 female, age = 24.2 ± 2.1 years, height = 175.3 ± 7.3 cm, weight = 72.4 ± 9.7 kg; Experiment 2: 9 male, 11 female, age = 24.1 ± 3.3 years, height = 178.1 ± 11.1 cm, weight = 70.0 ± 10.5 kg,* ***one participant completed both experiments****).*

Line 258: "Nevertheless, the time to reach steady state walking was shorter during backward as compared to forward walking initiation (t(19) = -6.051, p < 0.001, Figure 4B), while the maximal absolute

velocity in backward walking was significantly lower (Forward: 1.13 ± 0.17 m/s vs. Backward: 0.92 ± 0.14 m/s; t(19) = -11.3, p < 0.001, Figure 4B).”

Interesting. Any idea/speculation of why?

The backward walking speed was slower. Would that be a sufficient explanation?

We indeed hypothesize that the lower final speed of backward walking is responsible for this difference. We have included this interpretation.

> *Results - Preferred quiet standing posture is not affected by the metabolic cost of gait initiation (line 247)*
>
> *Between forward and backward walking with known directions, the time to reach steady state walking was **around 19% shorter** during backward as compared to forward walking initiation, likely because the maximal absolute velocity in backward walking was **also roughly 19% lower** (Figure 4B, **Table 2**).*

Line 275: “marginal”

For clarity and full disclosure, I would write "marginal but significant"

We agree that the reviewer's suggestion is clearer and have implemented a variant of it.

> *Discussion – Costs of gait initiation do not drive quiet standing posture (line 363)*
>
> ***While significant,** such marginal differences indicate that the energetic penalties of starting gait from a suboptimal stance may be too small to drive systematic adjustments, **especially when compared against the significant cost increase associated with lean angle (Figure 2A).***

Line 346: However, substantial within- and between-participant variability in preferred posture suggests that the minimization of energy expenditure does not require strictly matching a single energy-optimal reference posture.

I couldn't agree more.

It would be particularly beneficial to compare your conclusions with the existing literature on the topic.

Consider for example this seminal work by Kiemel et al.:

T. Kiemel, Y. Zhang, and J. J. Jeka, “Identification of Neural Feedback for Upright Stance in Humans: Stabilization rather than Sway Minimization,” Journal of Neuroscience, vol. 31, no. 42, pp. 15144–15153, Oct. 2011, doi: 10.1523/JNEUROSCI.1013-11.2011

We are content to hear that the reviewer agrees with our proposition, and we agree that the work by Kiemel et al. (2011) is highly relevant. The work was already cited in the section *Variability and flexibility in postural control*, but not here. For clarity, we have also included a citation at this location and also included a reference to experimental work by Houdijk et al. (2015) on how restricting sway can increase energy expenditure.

> *Discussion (line 321)*
>
> *However, substantial within- and between-participant variability in preferred posture suggests that **minimizing** energy expenditure does not require **tightly tracking** a single energy-optimal reference posture (**28, 39**).*

This same combination of references was reused later when discussing how the target may influence energy expenditure.

Discussion – Variability and flexibility in postural control (line 393)

*Strictly limiting **the permitted CoM or CoP** variability can increase energetic **expenditure due to the higher incidence and magnitude of postural corrections (28, 39)**, though energetic costs **also rise** when variability is **artificially** increased using more challenging experimental conditions (38).*

Line 355: "We also found that removing visual feedback did not significantly affect energy expenditure or lean angle"

Do you think the lack of a significant effect could also be a consequence of task duration?

Energy is not an instantaneous quantity but an integral one, so time matters.

The reviewer raises an interesting point about how the trial duration may affect energetic cost. Our unconstrained (i.e., preferred posture with eyes open and closed) trials lasted 5 minutes, and in S1 Appendix B, we assessed whether energetic cost varied over this window. We found no significant difference in the energetic cost between minute 2 and 3-5 during the eyes-open trial. For completion, we have also computed this difference for the eyes-closed trial, and found that there was also no significant effect of trial time on energy expenditure. We have added this observation to S1 Appendix.

S1 Appendix – Appendix B: Validation of trial length of indirect calorimetry measurements in Experiment 1

*To verify that 2 minutes in the target trials were sufficient to reach steady-state, we ran a rmANOVA on the preferred postural trials segmented into three periods: the first minute, the second minute, and the remaining three minutes (Figure A3 and Table A1). There was no significant differences across these three time periods for any of the metrics (Table A1). **We also performed the same test in the Eyes-Closed condition, and similarly found no significant effect of trial time on energy expenditure ($F_{(2,22)}$ = 0.493, p = 0.618).***

We also used this opportunity to improve the readability of the S1 Appendix B by placing the breathing metrics and statistics in Table A1 and rewriting the related text.

S1 Appendix – Table A1 (screenshot)

	EE Measured W/kg	*VE* L/s	*Rf* 1/min	*RQ* -
Target				
-1.15°	2.04±0.68	12.34 ± 4.45	19.7 ± 4.0	0.75 ± 0.06
0°	1.70±0.46	10.69 ± 3.44	17.8 ± 4.6	0.77 ± 0.06
1.15°	1.56±0.46	9.87 ± 3.06	18.3 ± 5.1	0.76 ± 0.05
2.29°	1.61±0.54	10.35 ± 3.31	18.1 ± 4.5	0.77 ± 0.06
3.44°	1.76±0.52	10.84 ± 3.31	18.1 ± 4.0	0.76 ± 0.05
5.73°	2.01±0.53	12.42 ± 3.81	20.5 ± 4.1	0.75 ± 0.07
EO	1.53±0.48	9.89 ± 3.32	14.4 ± 3.7	0.80 ± 0.06
EC	1.60±0.49	9.89 ± 3.43	13.8 ± 4.2	0.79 ± 0.05
Time-separated EO				
0-60s	1.55 ± 0.59	9.86 ± 3.99	14.8 ± 2.8	0.81 ± 0.08
60-120s	1.48 ± 0.48	9.56 ±3.27	14.3 ± 3.6	0.79 ± 0.07
120-300s	1.54 ± 0.47	10.01 ± 3.33	14.4 ±3.8	0.80 ± 0.07
rmANOVA statistics				
Target	$F_{(5,55)}$ = 13.831<br>p < **0.001**	$F_{(5,55)}$ = 6.84<br>p < **0.001**	$F_{(5,55)}$ = 7.59<br>p < **0.001**	$F_{(2.5,27.5)}$ = 0.789<br>p = 0.490
Time-separated EO	$F_{(2,22)}$ = 1.26<br>p = 0.304	$F_{(2,22)}$ = 0.844<br>p = 0.443	$F_{(2,22)}$ = 0.35<br>p = 0.708	$F_{(2,22)}$ = 0.775<br>p = 0.473

Legend: EE: Energy Expenditure, VE: Minute volume, Rf: Breathing frequency, RQ: Respiratory quotient. Post hoc tests for Target rmANOVA are provided in Appendix E.

We further note that during the creation of this supplementary table we discovered that one participant (S09) showed an abnormally elevated ventilatory equivalent (VE/VO2) across the standing trials. Both

ventilation (VE) and oxygen uptake (VO2) fell within normal ranges for quiet standing, which is why the issue was not apparent when these variables were examined separately. However, their ratio (all >80) was consistently much higher than in the other participants (26.02 ± 3.95 over all trials, closely matching previously reported rest values of around 25-30, (Sun et al., 2002)). This indicates that the VO2 was underestimated in this participant. A possible explanation could be that the sampling tube flow was being restricted (e.g., due to a kinked cable or excessive condensation) or not being fully secured to the mask or analyzer. Because this discrepancy could not be corrected, we excluded the metabolic data of this participant from the analyses. No other participants showed this pattern. This exclusion was added to the Methods and has resulted in only minor changes in the energetic results and statistics, and no change in the conclusions drawn from the study.

> *Materials and Methods (line 480)*
>
> ***Indirect calorimetry data were excluded for one participant (S09) because their ventilatory equivalent (VE/VO2) was highly elevated compared to that of the other participants (26.0 ± 4.0 for the remaining included participants, around previously reported rest ranges of 25-30 (97)).***

Line 386: "Even initiating gait from the least efficient posture tested (-2 [illegible] 10-2 rad), which increased energy expenditure by 0.42 ± 0.27 J/kg relative to the minimum-cost posture, would raise total expenditure over 10 m of walking by only ~1%. Such marginal differences indicate that the energetic penalties of starting from a suboptimal stance are too small to drive systematic adjustments."

Very interesting!

We thank the reviewer for their kind words.

Line 406: "This suggests that a participant's preferred posture is not solely the result of minimizing active contributions to the ankle moment, consistent with earlier work showing that humans typically adopt a more forward-leaning stance than predicted by inverted pendulum models (23, 62)."

Not only that, there is substantial evidence that ankle stiffness is not enough to sustain upright posture:

Casadio M, Morasso PG, Sanguineti V. Direct measurement of ankle stiffness during quiet standing: implications for control modelling and clinical application. Gait Posture. 2005 Jun;21(4):410-24. doi: 10.1016/j.gaitpost.2004.05.005. PMID: 15886131.

Morasso PG, Schieppati M. Can muscle stiffness alone stabilize upright standing? J Neurophysiol. 1999 Sep;82(3):1622-6. doi: 10.1152/jn.1999.82.3.1622. PMID: 10482776.

Consider including these references and comment on their role with respect to your results.

We agree that the observation that ankle stiffness alone cannot stabilize the human body, and that this observation is relevant to discussing how variability in ankle stiffness could potentially drive postural changes. We have included the two recommended references (Casadio et al., 2005; Morasso & Schieppati, 1999), as well as work by Morasso and Sanguineti (2002) on the topic.

> *Discussion – Variability and flexibility in postural control (line 382)*
>
> *This suggests that a participant's preferred forward lean is not solely the result of minimizing active contributions to the ankle moment, consistent with earlier work showing that humans typically adopt a more forward-leaning stance than predicted by inverted pendulum models (23, 71)* ***and that ankle stiffness alone cannot stabilize the human body (72-74).***

Given the role of ankle stiffness in energy expenditure during standing, we have also included reference to Casadio's work when suggesting future work on this topic (line 434, see above).

Line 418: "Intermittent balance control models (66-68) formalize this tradeoff of effort and variability by permitting postural sway until a stability threshold is exceeded, thereby reducing energetic demands compared to continuous control."

I agree, and want to add that this need for intermittency might be connected to the hypothesis that humans control posture in terms of neural descending velocity commands.

Please refer to this work that combines the idea of intermittency and velocity control by means of postural sway data:

Tessari, F., Hermus, J., Sugimoto-Dimitrova, R. et al. Brownian processes in human motor control support descending neural velocity commands. Sci Rep 14, 8341 (2024). https://doi.org/10.1038/s41598-024-58380-5

We thank the reviewer for sharing this interesting work by Tessari et al. (2024), and have incorporated it in a brief interpretation of how Brownian motion/postural drift plays a role in energy expenditure. As our current study does not address the mechanisms leading to Brownian motion, we did not further discuss the role of velocity commands in evoking this behavior. Please find the changes in the manuscript using the work by Tessari et al. (2024) in our reply to the comments on original **Lines 99, 172, 528** above.

Line 442: "Moseley, Crosbie (42) reported a passive ankle moment of only ~10 Nm at this angle."

See previously recommended works by Morasso and Casadio.

Line 696: "We performed a Monte Carlo simulation based on the parameter distributions for ankle stiffness reported by Moseley, Crosbie (42)."

There are other works providing ankle stiffness values. Was there a specific reason to choose Moseley's work?

We thank the reviewer for their two comments on ankle moment and recommending the proposed literature (Casadio et al., 2005; Morasso & Schieppati, 1999). The model by Moseley et al. (2001) was used because it provided ankle stiffness as a function of ankle angle (in contrast with many studies that report a singular value) and included uncertainty ranges for the fit parameters. This allowed us to simulate the variability in equilibrium lean angle as a function of ankle stiffness by stochastically generating ankle stiffness curves. This reasoning has been included in the Materials and Methods.

> *Materials and methods – Monte-Carlo analysis of ankle stiffness and equilibrium angle (line 697)*
>
> *We performed a Monte Carlo simulation based on the parameter distributions for ankle stiffness reported by Moseley, Crosbie (42),* ***as this model includes the progressive increase of ankle stiffness with ankle angle (42, 54) and provides the uncertainty ranges of the fit parameters, allowing the stochastic generation of ankle stiffness curves****.*

Nevertheless, we agree that the listed stiffness could be contextualized with other work. We have added the work by Le Sant et al. (2017), which also predicts an ankle stiffness of ~10 Nm at this angle, and the work by Casadio et al. (2005) and Vlutters et al. (2015) in recommendations for future research (line 436, see above).

Line 648: "In Experiment 2, only one force plate was available for our experiments."

MINOR: Why? Did you perform the 2 experiments in different facilities? Was the 2nd platform unnecessary?

Experiments 1 and 2 were performed at different facilities due to practical constraints. Specifically, when Experiment 2 was conducted, the first facility housing two force plates was no longer available. As a result, the second experiment used a single force plate, and a force-splitting algorithm was applied to estimate the contribution of each foot. As shown in S1 Appendix A, this method reliably reconstructed ground reaction forces during gait initiation in healthy participants.

> *Materials and Methods - Estimation of ground reaction forces from single force plate data (line 684)*
>
> *In Experiment 2,* ***a single*** *force plate was* ***embedded in a long walkway extending in the forward and backward direction to allow gait initiation in either direction. Because only one plate was available, individual foot ground reaction forces were reconstructed using a kinematics-assisted force-splitting algorithm, which was validated against dual-force plate data (see S1 Appendix).***

Line 719 and Line 735: "Holm correction"

Why Holm and not Sidak or Bonferroni? Please clarify.

We chose the Holm correction because our post-hoc comparisons were repeated-measures tests across related postural conditions. The Holm correction does not require hypotheses to be independent, which is an assumption for some other corrections (e.g., Sidak). The Bonferroni correction also does not require independence, but is rather conservative and could unnecessarily increase false negatives.

We have consolidated our reasoning for using the Holm correction into one sentence and added a justification for its use:

> *Materials and Methods - Statistical tests (line 718)*
>
> ***A Holm correction was applied to all planned post hoc comparisons, with corrected p-values indicated by a subscript where applicable. This method does not require independence among hypotheses and is therefore suitable for repeated-measures designs.***

Reviewer #2:

The present manuscript investigates the energetic cost as a function of body inclination angle and in relation to gait initiation, combining experimental measurements (incl. calorimetry) with musculoskeletal simulations. The study aims to critically evaluate the commonly applied assumption of symmetric energetic components in anterior and posterior lean directions.

The topic is highly relevant and well aligned with the scope of PLOS Computational Biology. The chosen methodological approach is appropriate for addressing the research question, and both the rationale and the overall methodology are clearly and logically presented.

While the manuscript is generally well written and readable, its structure is unfortunately highly redundant in several parts. For example, lines 73–86 as well as Figure 1 do not belong into the Introduction. Moreover, the boundaries between the Introduction, Materials & Methods, and Results sections are often blurred, which affects clarity. A more streamlined organization following conventional manuscript structure is strongly recommended.

We agree with the reviewer that the manuscript had redundant passages and that the structure could be tightened. Below, we highlight the major changes we made to address this.

In the introduction, we shortened the last paragraph, which summarized the study. This paragraph is now only a basic description of the aims of this study and of what we did to achieve them (see below).

> *Introduction (line 71)*

> *In this study, we examined (i) how metabolic cost varies with standing posture, (ii) how posture affects the energetic cost and time required to complete gait initiation, and (iii) whether humans optimize posture to minimize these costs during quiet standing and in preparation for walking. In Experiment 1 (N = 13), we measured kinematics, metabolic cost, and muscle activity as participants stood at a preferred posture and six prescribed sagittal-plane postures, and simulated energy expenditure using a musculoskeletal model. In Experiment 2 (N = 20), we used our validated musculoskeletal model to estimate the metabolic cost of initiating gait from three prescribed postures or each participant's preferred posture in both forward and backward directions. Our results suggest that human postural control approximates optimal energy expenditure of balance and gait initiation by operating near, but not strictly at, a slight anterior minimum-cost whole-body lean angle.*

We note that in our original submission, Figure 1 was intended to be part of the Results section but was incorrectly placed in the Introduction. The figure is now correctly displayed after the first Results subsection, where it is first referenced.

In the Results, we have removed the summarizing paragraph that opened the section, as this section contained more methodological detail than was necessary to understand the presented results. A highly condensed description of each condition is now provided in the paragraph where that condition is first presented, while the rest of the methodological detail is left to the Materials and methods section.

> *Results - Energetic cost landscape of human standing balance (lines 81 & 89)*
>
> *In our first experiment (N = 13), we measured participants' energy expenditure at preferred and prescribed postures using indirect calorimetry to determine how energy expenditure varies over natural postures.* ***During preferred posture trials, participants self-selected a posture throughout the trial (eyes open and eyes closed, 2 x 5 min each, Figure 1A, C, E).***
>
> *...*
>
> ***In the target condition trials, six whole-body lean angles were prescribed (-1.15, 0, 1.15, 2.29, 3.44, and 5.73°) using real-time feedback of the center of pressure (CoP) position on a screen (Figure 1A, D).*** *The energetic cost measured through indirect calorimetry was minimal at the 1.15° target (anterior) and increased in both lean directions (Figure 2A).* ***For visualization, the offset due to the basal metabolic cost (i.e., the energy expended by non-motor processes to uphold bodily function) and model simplifications (i.e., muscles beyond the lower limbs) was removed by subtracting the cost of the preferred posture trial with eyes open per participant for both the measured and simulated data (Figure 2A; data without this subtraction are shown in the inset).***

In the discussion, we have removed most numerical results unless they were relevant for contextualizing with other work. We shortened the opening paragraph of the discussion to provide a brief general discussion before moving on to the specific subsections (line 314).

> *Discussion (line 314)*
>
> *In this study, we examined (i) how metabolic cost varies with* ***lean angle****, (ii) how the cost of gait initiation depends on posture, and (iii) whether humans adopt postures that minimize cost during quiet standing and in preparation for walking. In Experiment 1,* ***we found that energy expenditure in standing increased*** *asymmetrically with lean angle****, with a minimum*** *at a slight forward lean****, and that participants preferred lean angles*** *close to this energetic minimum. In Experiment 2, participants continued to prefer a forward-leaning orientation and did not adjust their posture for gait initiation, even when the upcoming gait direction was known. Consistent with this****, our musculoskeletal*** *simulations showed that the energy cost of gait initiation was also near optimal at a slight forward lean, regardless of the direction of gait initiation. Taken together, our results support the idea that humans minimize energy expenditure during quiet standing. However, substantial within- and between-participant variability in preferred posture suggests that*

> ***minimizing** energy expenditure does not require **tightly tracking** a single energy-optimal reference posture (28, 39). These findings provide insight into how energy expenditure may underlie our everyday movement control and challenge the common modeling assumption that postural costs can be accurately **described by assuming** a symmetric error from a **fixed** reference posture (e.g., vertical or static equilibrium **when incorporating passive** ankle stiffness).*

We have shortened and rewritten the Conclusion to avoid redundancy with the opening of the discussion and the abstract.

> *Conclusion (line 440)*
>
> ***By combining indirect calorimetry and musculoskeletal simulations, we quantified the energetic cost of quiet standing and gait initiation across a range of natural standing postures. Participants maintained their preferred posture close to the minimum-cost lean angle during static standing. The anticipation of gait initiation in either direction did not alter this preference, likely because small adjustments in lean angle only lead to changes in energetic cost and duration that are inconsequential compared to continuous walking. These findings provide insight into how energy expenditure drives our everyday balance control and challenge the common modeling assumption that postural costs can be accurately described by symmetric errors from a single reference posture. By integrating empirical and simulation-based approaches, our work offers a broader perspective on the cost functions that define optimal postural control and highlights opportunities to improve them.***

Lastly, we kept the Materials and Methods as the last section, as this is the structure in the PLOS Computational Biology guidelines and benefits the readability of the manuscript.

With respect to measurement techniques, simulations, and data analysis, the study has been conducted according to current standards and best-practice recommendations.
A major point of concern relates to the employed musculoskeletal model. Notably, the model does not include trunk musculature (e.g., m. erector spinae) or muscles of the upper extremities. This omission raises questions regarding the completeness of the energetic analysis.

We agree that the manuscript would benefit from clarifying the implications of this modeling choice. Our aim was to quantify how energetic cost changes with lean angle and gait initiation, and we acknowledge that trunk muscles, including the erector spinae, contribute to upright posture and could influence these costs. This omission was not intended to imply that trunk musculature is unimportant, but rather reflects a tradeoff between physiological completeness and the specific requirements of our energetic analysis.

Our pilot studies and current data suggest that omitting trunk musculature may have had a limited effect on the main conclusions. Specifically, measured erector spinae EMG activity did not increase substantially at more forward-leaning postures (Figure 3) and was the lowest activated muscle group measured, suggesting that the contribution of back muscles to the increase in energy expenditure in these conditions may be modest. To validate that this trend was consistent with the moment required to stabilize the trunk, we have plotted the model-simulated lumbar extension moment in Figure 3 along with the change in EMG. The lumbar extension moment follows the trend of the erector spinae muscle, suggesting a small increase in EMG activity with lean angle. We added a description of this observation to the Results section.

> *Figure 3 – Trunk extension moment (screenshot)*

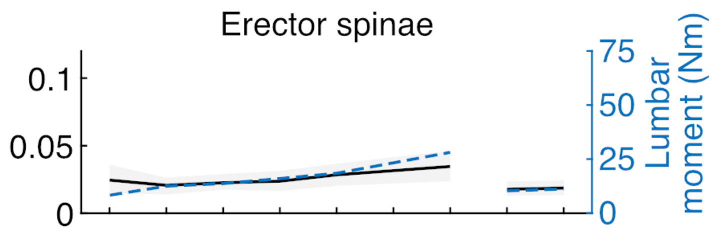

> *Results - Musculoskeletal model simulations predict the trend of metabolic cost in posture (line 204)*
>
> ***Although the erector spinae was not included in our simulations, we compared the model's lumbar extension moment to the experimentally measured muscle activity. Both signals followed a slight upward trend, suggesting that the contributions of trunk muscles gradually increase with greater anterior leaning.***

Nevertheless, we believe it would be useful to more clearly state that trunk muscle contributions were not characterized in the current study and address any limitations that may arise from this choice. In the manuscript, we have expanded details regarding this choice in the Discussion.

> *Discussion: Towards improved models of cost in posture (line 426)*
>
> ***While the model estimated the shape of the metabolic cost landscap****e, it underestimated the increase in energetic cost for anterior lean angles (Figure 2A).* ***One possibility is that contributions from muscles not included in the model, such as those of the back, arms, and neck, increase energy expenditure during anterior postures. The measured EMG activity of the erector spinae muscle (Figure 3) showed a small increase in activity with more anterior postures, suggesting that it could contribute to changes in energy expenditure together with other trunk muscles, though this trend was considerably smaller than that observed in the plantarflexor muscles.***
>
> *…*
>
> *Future work could investigate how* ***stabilization of the spine and upper body****, individual (sex) differences in morphology and muscle strength (88, 89), weight distribution, physiological and behavioral changes with ageing (61, 90), and passive ankle stiffness (46, 72, 91) affect energy expenditure and optimal posture.*

Lastly, we clarified the selection of the current model and moment comparison in the Methods section:

> *Methods - Musculoskeletal analysis of motion capture data (lines 624 & 657)*
>
> *Musculoskeletal analyses were performed using OpenSim (14) (version 4.3) with the Rajagopal lower limb musculoskeletal model (13).* ***This model was selected because it provides improved representations of lower-limb muscle-tendon geometry and fiber length, particularly of the plantarflexors, and has been widely used to simulate human gait (e.g., 45, 108).***
>
> *…*
>
> *To verify simulated muscle activity, it was compared to the EMG data of 7 major muscles contributing to postural control in Experiment 1.* ***The erector spinae was measured but not simulated, so it was compared against the simulated lumbar extension moment between the hip and torso instead.***

Furthermore, there are inconsistencies between measured and simulated muscle activities. The m. erector spinae is not simulated at all, while gluteal muscles are included in the simulation but neither shown in Figure 3 nor assessed via EMG measurements. Given the known relevance of these muscle

groups for posture and locomotion, it is likely that they substantially contribute to overall energy expenditure.

Despite these limitations, the physiological interpretation of the results and the derived conclusions are well reasoned and plausible.

Although we are unable to include additional measured muscle (e.g., gluteal muscles), we have now provided detailed activity profiles for all muscles included in the simulation for Experiments 1 and 2 (see Figure S1-S3 and below). With this figure, readers can assess how the activation of any muscle of interest changes across simulated postures and during gait initiation. Please see separately the uploaded Supplementary information file for full resolution.

> *Supplementary Figure S1*
>
> ***S1 Figure. Experiment 1 Simulated Muscle Activations during Standing.*** *Figure showing the simulated muscle activations of all 80 muscles during the target and preferred posture trials of Experiment 1, compared against EMG data where available. Labels correspond to muscle name abbreviations used in the Rajagopal et al. (2016) musculoskeletal model.*

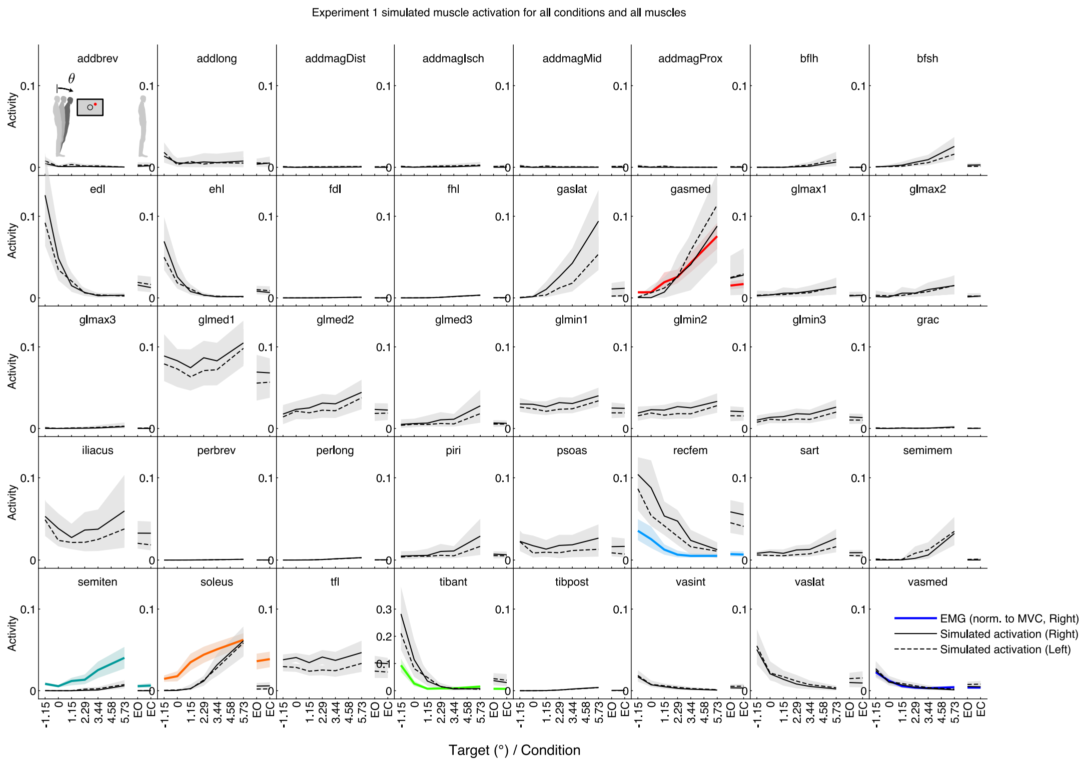

> *Supplementary Figure S2*
>
> ***S2 Figure. Experiment 2 Simulated Muscle Activations during Forward Gait Initiation.*** *Figure showing the simulated muscle activations of all 80 muscles during forward gait initiation, split between the stance and swing leg, and for each starting lean angle.*

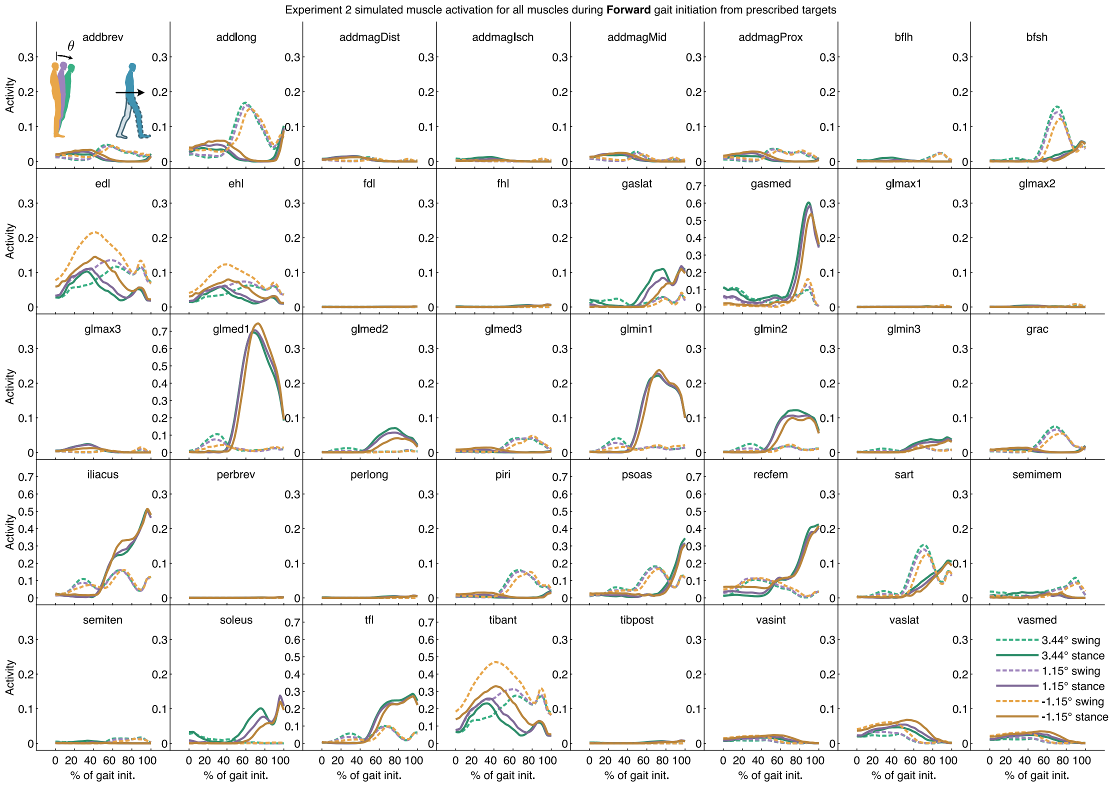

Supplementary Figure S3

S3 Figure. Experiment 2 Simulated Muscle Activations during Backward Gait Initiation.
Figure showing the simulated muscle activations of all 80 muscles during backward gait initiation, split between the stance and swing leg, and for each starting lean angle.

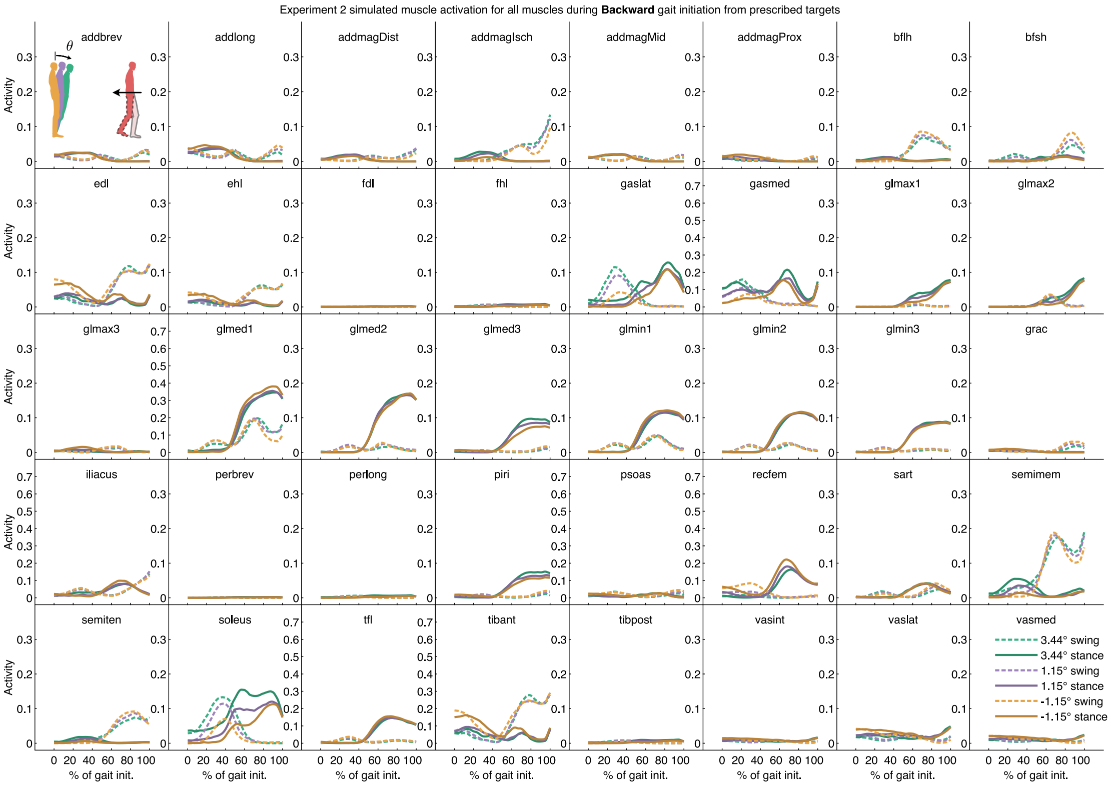

From this analysis, we observed that the gluteal muscles contribute to standing and its energy expenditure as they are active over all postures (glmax, glmed, and glmin in the Figure S1 above). The figure shows that these contributions scale modestly with greater forward lean, but the trend is weaker than in the plantarflexor muscles. A brief statement about the gluteal muscles and references to the Supplemental Figures were added to the Results.

> *Results - Musculoskeletal model simulations predict the trend of metabolic cost in posture (line 200)*
>
> *Muscles responsible for dorsiflexion (tibialis anterior) and knee extension (rectus femoris, vastus medialis) were highly active in backward postures, with activation declining as the body leaned forward. In contrast, forward postures increased reliance on ankle plantarflexors (soleus, gastrocnemius) and hip extensors (semitendinosus, semimembranosus).* ***The gluteal muscle activation in our simulation results also increased with lean angle, but to a lesser extent than the plantarflexors (see S1 Figure for activations of all simulated muscles).***

Another point requiring clarification is that the accuracies of the experimental measurement systems are not reported, whereas simulation accuracies are described. Including this information would strengthen the methodological transparency.

The reviewer is correct that the accuracy of the measurement systems was not reported. We have included the measurement system accuracies in the methods section.

Metabolic analyzer

The manufacturer does not report specific accuracy ranges, but a number of validation studies include rest conditions (Crouter et al., 2019; Guidetti et al., 2018), which we have now incorporated into the methods. The bootstrapped confidence intervals in Figure 2A (and subsequent statistical tests) demonstrate that the observed increase in energetic cost with lean angle exceeds the measurement uncertainty of indirect calorimetry between participants.

> *Materials and Methods - Experiment 1: Static standing protocol (line 472)*
>
> *Energy expenditure was measured throughout the experiment using a breath-by-breath indirect calorimetry metabolic analyzer (K5 9260AA6, COSMED, Italy).* ***This type of indirect calorimetry system has been validated during resting conditions against Douglas bag measurements and metabolic simulators (93, 94).***

Force plates

We retrieved the following accuracies from manufacturer specifications and have included them in the Materials and Methods.

> *Materials and Methods – Experiment 1 (line 470)*
>
> *(9260AA6, Kistler Group, Winterthur, Switzerland;* ***CoP error < 2.0 mm****)*

> *Materials and Methods – Experiment 2 (line 546)*
>
> *(Plate: BMS 400600HF-1K, Amplifier: Optima OPT-SC, AMTI, MA, US;* ***CoP error < 0.5 mm****)*

Motion capture:

To document motion capture measurement accuracy, we have included the SD of the calibration wand length from the calibration files and the marker fill percentages (i.e., the percentage of the trial during which a marker is not obstructed) for both experiments.

> *Materials and Methods - Musculoskeletal analysis of motion capture data (line 621)*
>
> ***The fill percentage was 99.9 ± 0.3% in Experiment 1 and 99.9 ± 0.5 % during the simulated part of gait initiation in Experiment 2. The standard deviation of the wand length during calibration was <1.1 mm for all measurements.***

The data sharing plan is exemplary, clearly described, and fully in line with open science principles. In summary, while the manuscript requires revision, particularly regarding model completeness, structural clarity, and consistency between experimental and simulation data, the study shows strong potential. Provided that the issues outlined above are adequately addressed, I believe the manuscript would be suitable for publication in PLOS Computational Biology.

We thank the reviewer for their valuable comments on improving the manuscript and believe these revisions have improved its quality and rigor.

Reviewer #3:

This manuscript addresses (i) how metabolic cost varies with standing posture, (ii) how the cost of gait initiation depends on posture, and (iii) whether humans adopt postures that minimize cost during quiet standing and in preparation for walking. These are interesting and relevant research questions, and the work has the potential to make a valuable contribution to the field. The study appears to be based on a substantial amount of experimental work, and the general approach is methodologically sound.

However, in its current form the manuscript requires major revisions before it can be considered for publication. It would benefit from substantial restructuring to improve readability and transparency. Overall, the manuscript is very dense and contains many sentences that repeat similar information. I would strongly recommend major rewording and editing to reduce the length and improve the clarity of the text.

My detailed comments and suggestions are provided below and are intended to help the authors improve the quality and clarity of the manuscript.

1 – Results

I have a major suggestion regarding the presentation of the results. Because the study includes two experiments and multiple outcome metrics, the current presentation is difficult to follow.

First, I recommend choosing a single unit for angular measures (either radians or degrees). Reporting both units throughout the results makes the manuscript unnecessarily confusing.

We agree with the reviewer that the mixed presentation of units is not helpful for the clarity of the manuscript. We decided to convert all angular units in the manuscript to degrees, as this reporting is more intuitive to readers.

The selection of the targets was originally based on linear radian increments, so this explanation was included in the methods section.

> *Materials and methods - Experiment 1: Static standing protocol*
>
> *While the target circle was always drawn in the center of the screen, participants had to maintain a lean angle ⍰target of -2, 0, 2, 4, 6, or 10 · 10^{-2} rad* ***(i.e., -1.15, 0, 1.15, 2.29, 3.44, 5.73°)*** *to get the cursor in the center. These lean angles correspond to a subset of the targets used by Mensink, Nasrabadi (75), where the variability of ankle torques was investigated.*

Second, many of the numerical results could be presented more effectively in tables rather than repeatedly embedded in the text. This would substantially improve the readability of what is currently a very dense Results section.

We agree that excessive inline results harmed the section's readability. We have created Tables 1 and 2 to avoid repetitive reporting.

> *Table 1 - (screenshot)*
>
> **Table 1: Energy expenditure (measured and simulated) and balance characteristics from Experiment 1.**

	EE Measured			EE Simulated			θ	θ_{SD}	CoP v_{AP}
	W/kg	t(11)	p	W/kg	t(12)	p	°	°	mm/s
Target									
-1.15°	2.04±0.68	5.180	**<0.001**	1.09±0.31	5.034	**<0.001**	-1.01 ± 0.08	0.23±0.04	26.6±7.0[b]
0°	1.70±0.46	2.987	**0.012**	0.81±0.19	3.009	**0.016**	0.07 ± 0.07	0.24±0.05	20.5±4.3
1.15°	1.56±0.46	-		0.69±0.13	-		1.17 ± 0.07	0.22±0.05	20.6±5.5
2.29°	1.61±0.54	0.650	0.265	0.70±0.13	0.677	0.318	2.29 ± 0.08	0.21±0.04	20.8±6.8
3.44°	1.76±0.52	3.539	**0.007**	0.72±0.10	1.043	0.318	3.41 ± 0.08	0.21±0.04	21.1±5.1
5.73°	2.01±0.53	11.504	**<0.001**	0.96±0.17	5.453	**<0.001**	5.66 ± 0.09	0.29±0.06[a]	28.7±7.6[b]
Preferred posture									
EO	1.53±0.48	-0.358[c]	0.727	0.67±0.11	-0.640[c]	0.535	1.50 ± 0.73	0.49±0.18	20.9±6.9
EC	1.60±0.49	2.104[d]	0.059	0.68±0.12	0.838[d]	0.418	1.68 ± 0.75	0.53±0.22	24.6±8.5

Legend: EO: Eyes Open, EC: Eyes Closed, EE: Energy Expenditure. Post-hoc statistics for rmANOVA are provided in S1 Appendix. [a]Significant difference w.r.t. all other targets, [b]Significant difference w.r.t. target 0 to 3.44°, [c]: t-test against target 1.15°, [d]: t-test against EO.

> *Table 2 - (screenshot)*

> **Table 2: Results and statistics for Experiment 2.**
>
> | | θ | $t_{v\,90\%}$ | v_{peak} | E_{musc} | Δx_{com} | CoT |
> |---|---|---|---|---|---|---|
> | | ° | s | m/s | J/kg | m | J/m/kg |
> | Gait initiation from preferred posture | | | | | | |
> | B only | 0.96 ± 1.06 | 1.26 ± 0.20 | 0.92 ± 0.14 | 2.00 ± 0.27 | 0.23 ± 0.03 | 8.98 ± 1.44 |
> | Random : B | 1.01 ± 1.01 | 1.25 ± 0.15 | 0.90 ± 0.15 | 1.99 ± 0.26 | 0.23 ± 0.04 | 9.05 ± 1.56 |
> | Random : F | 1.00 ± 1.03 | 1.62 ± 0.21 | 1.12 ± 0.17 | 2.27 ± 0.27 | 0.34 ± 0.04 | 6.71 ± 0.94 |
> | F only | 1.00 ± 1.01 | 1.56 ± 0.23 | 1.13 ± 0.17 | 2.25 ± 0.32 | 0.35 ± 0.05 | 6.53 ± 0.96 |
> | Random vs only (B) | $t_{(19)} = 0.80$
$p = 0.432$ | $t_{(19)} = -0.22$
$p = 0.830$ | $t_{(19)} = -1.72$
$p = 0.101$ | $t_{(19)} = -0.43$
$p = 0.669$ | $t_{(19)} = -0.27$
$p = 0.787$ | $t_{(19)} = 0.28$
$p = 0.780$ |
> | Random vs only (F) | $t_{(19)} = 0.07$
$p = 0.948$ | $t_{(19)} = 1.52$
$p = 0.146$ | $t_{(19)} = -1.26$
$p = 0.223$ | $t_{(19)} = 0.86$
$p = 0.398$ | $t_{(19)} = -1.78$
$p = 0.091$ | $t_{(19)} = 2.23$
$p =$ **0.038** |
> | B only vs F only | $t_{(19)} = -0.62$
$p = 0.543$ | $t_{(19)} = -6.02$
$p <$ **0.001** | $t_{(19)} = -11.29$
$p <$ **0.001** | $t_{(19)} = -5.21$
$p <$ **0.001** | $t_{(19)} = -13.65$
$p <$ **0.001** | $t_{(19)} = 10.77$
$p <$ **0.001** |
> | Gait initiation from target | | | | | | |
> | B : -1.15° | -1.19 ± 0.09 | 1.11 ± 0.27 | 0.95 ± 0.17[b] | 2.03 ± 0.29[b] | 0.22 ± 0.04[b] | 9.60 ± 1.97[b] |
> | B : 1.15° | 1.06 ± 0.07 | 1.09 ± 0.21 | 0.96 ± 0.15 | 1.99 ± 0.34[b] | 0.23 ± 0.04[ab] | 8.85 ± 1.66[a] |
> | B : 3.44° | 3.34 ± 0.07 | 1.16 ± 0.22 | 0.97 ± 0.15[a] | 2.18 ± 0.37[a] | 0.26 ± 0.03[a] | 8.69 ± 1.63[a] |
> | F : -1.15° | -1.19 ± 0.08 | 1.57 ± 0.18[b] | 1.19 ± 0.22 | 2.70 ± 0.32[b] | 0.38 ± 0.07[b] | 7.34 ± 1.93[b] |
> | F : 1.15° | 1.05 ± 0.04 | 1.47 ± 0.25 | 1.18 ± 0.21 | 2.29 ± 0.41[a] | 0.36 ± 0.06[a] | 6.48 ± 1.41[a] |
> | F : 3.44° | 3.34 ± 0.07 | 1.37 ± 0.24[a] | 1.18 ± 0.20 | 2.25 ± 0.42[a] | 0.35 ± 0.06[a] | 6.67 ± 1.57[a] |
> | B (all targets) | - | $F_{(2,38)} = 1.83$
$p = 0.174$ | $F_{(2,38)} = 4.92$
$\mathbf{p = 0.013}$ | $F_{(2,38)} = 10.72$
$\mathbf{p < 0.001}$ | $F_{(1.9,35.9)} = 28.28$
$\mathbf{p < 0.001}$ | $F_{(1.4,26.1)} = 5.11$
$\mathbf{p = 0.023}$ |
> | F (all targets) | - | $F_{(2,38)} = 7.71$
$\mathbf{p = 0.002}$ | $F_{(1.4,27.0)} = 0.88$
$p = 0.394$ | $F_{(1.3,24.9)} = 41.66$
$\mathbf{p < 0.001}$ | $F_{(1.5,27.7)} = 16.66$
$\mathbf{p < 0.001}$ | $F_{(1.2,23.3)} = 8.07$
$\mathbf{p = 0.006}$ |
>
> Legend: B: Backward, F: Forward. See Figure 4 for variable definitions. Post-hoc statistics for rmANOVA are provided in S1 Appendix. [a]Significant difference w.r.t. -1.15°, [b]Significant difference w.r.t. 3.44°.

In the Results, we have included qualitative statements about important changes in the data with reference to these tables. For example:

> *Results - Preferred quiet standing posture is not affected by the metabolic cost of gait initiation (line 257)*
>
> *During the sets with known walking direction (Forward and Backward only sets), **the CoT of backward walking was ~38% higher than that of forward walking (Figure 4B, Table 2)**.*

With the inclusion of these tables, the Supplementary Tables S1 and S2 are no longer required and have been removed. The Results section text now contains far fewer numerical and statistical data, making it more readable.

In addition, the reporting of p-values is inconsistent. Sometimes values are reported as "p < ..." and sometimes as "p = ...", even for significant results. I suggest adopting a uniform reporting style throughout the manuscript (for example, using "p = ..." and reserving "p < .001" only when appropriate). Consistency would improve the clarity and professionalism of the presentation.

We identified 4 instances where we wrote "*p* = <0.001", and have rectified those to "*p* < 0.001".

In one of the tests, the *p*-value was rounded down to 0.001; here, the value is still written as *p* = 0.001.

To improve statistical clarity, we have included significance bars in Figures 2 and 4.

> *Figure 4 - Example significance bars (screenshot)*
>
> 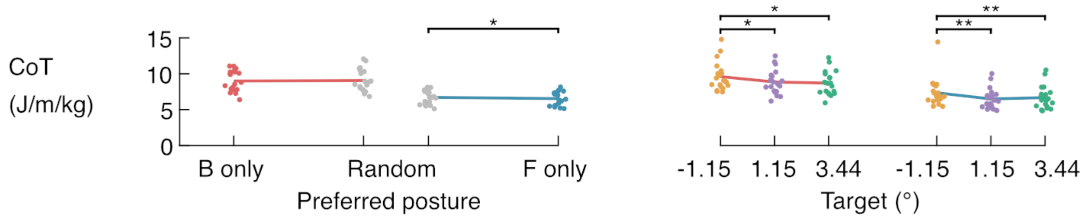
>

2 – Methods and statistical analysis

The methods and statistical analyses are generally understandable, but this section is also quite dense and repetitive. It could be streamlined substantially.

We thank the reviewer for their feedback on the statistical tests section. We have rewritten it to address concerns from all reviewers. The new section avoids repeating information about statistical tests and is generally more readable.

> *Statistical tests (line 712)*
>
> *Statistical analyses were performed in JASP (120)* ***(repeated-measures analysis of variance; rmANOVA)*** *and MATLAB (t-tests), using a significance level of 0.05. Variables are expressed using mean ± standard deviation unless mentioned otherwise.* ***Because all conditions were measured within participants, statistical tests were performed using within-subject comparisons. Factors with more than two levels were analyzed using rmANOVA, and specific hypotheses were evaluated using paired-samples t-tests. Degrees of freedom are reported between brackets for t-tests and rmANOVA outcomes.*** *When sphericity was violated, Greenhouse-Geisser corrections were applied.* ***A Holm correction was applied to all planned post hoc comparisons, with corrected p-values indicated by a subscript where applicable. This method does not require independence among hypotheses and is therefore suitable for repeated-measures designs.***
>
> *In Experiment 1,* ***we assessed how lean angle affected standing by analyzing how energy expenditure (measured and simulated), CoP velocity, and lean angle SD varied across the six target lean angles (Table 1). If the main effect was significant, univariate post-hoc comparisons between targets were conducted. For energy expenditure (measured and simulated), post hoc tests were limited to using one-sided t-tests comparing each target to the target with the minimum energetic cost (1.15°). Next, we compared the eyes-open preferred posture trial against the preferred posture with eyes closed and the minimum-cost target to assess the influence of vision and the target, respectively. Lastly, we compared the slopes of energetic cost change over lean angle between the anterior and posterior directions to assess how bodily asymmetry affects the rate of energetic cost increase.***
>
> *In Experiment 2, we analyzed the lean angle prior to gait initiation along with various additional gait initiation metrics* ***(time to reach steady-state, peak velocity, energy expended, center of mass displacement, cost of transport). To test how prior knowledge of walking direction influenced behavior,*** *we compared the Random block to the Forward-only and Backward-only blocks. Additionally, we compared the Forward-only and Backward-only blocks to assess differences* ***between*** *forward and backward gait. To examine the effect of the three prescribed lean angles on forward and backward gait initiation* ***during Target trials, we analyzed the target trials using separate one-way rmANOVAs. Forward and backward initiations were analyzed separately because the functional meaning of a given lean angle depends on walking direction (e.g., a backward lean is congruent for backward but incongruent for forward walking), leading to crossover interactions that would complicate interpreting a two-way ANOVA.***

Lastly, we removed the mention of one statistical test in the Methods (Experiment 1, Eyes Open condition against all targets) that was not presented in the Results. This test was not reported and would provide little insight compared to the test of the minimum-cost target vs the other targets.

Several sentences list many outcome variables in long chains that could be simplified.

A brief description of the analyzed variables is listed in the Statistical analysis section (see rewritten section Statistical tests above).

Some methodological details are explained multiple times (e.g., use of Holm correction, paired-samples t-tests).

We have consolidated our justification for Holm as multiple testing correction into a single sentence (see rewritten section Statistical tests above).

The rationale for separating forward and backward analyses is reasonable, but the explanation is somewhat wordy and could be condensed.

We agree that the rationale can be condensed without losing scientific rigor. We have shortened the description (see at the end of the rewritten section Statistical tests above).

3 – Discussion

In several places, the Discussion includes detailed numerical results. I find that repeating exact numerical values in the Discussion tends to overwhelm the reader and is unnecessary. I would recommend removing most of these numbers and focusing on interpretation instead.

We have removed specific numerical results from most parts of the Discussion, unless they were specifically compared against other values from the literature. This primarily applies to the energetic cost outcomes in the section "Costs of gait initiation do not drive quiet standing posture".

In response to previous reviewers, we also shortened the opening paragraph of the discussion, which provided a general discussion of the study's most important results (line 315).

When discussing control strategies and the simulation results, it would be valuable to acknowledge related work in this area. In particular, the authors may wish to cite studies by K. Shiozawa, J. Lee, M. Russo, D. Sternad, and N. Hogan (e.g., Journal of NeuroEngineering and Rehabilitation, 2021; Journal of Neurophysiology, 2024), which address similar issues and would help place the current findings in a broader context.

We thank the reviewer for their suggestions of relevant works. We found the JNER study by Shiozawa et al. (2021) to be especially relevant to the current work and have incorporated it into the discussion and introduction (see also our reply to Reviewer 1's suggestion to include this work).

> *Discussion – Towards improved models of cost in posture (lines 410 & 417)*
>
> ***More broadly, it is also important to consider whether a model's mechanical representation may limit inferences about energy expenditure. A single-link inverted model cannot capture multi-segment coordination and may not accurately represent higher-frequency dynamics (30, 84), possibly underestimating the control effort and highlighting the need to select appropriate model mechanics and cost functions for the problem at hand.***

Minor comments

Lines 77–86: I would avoid foreshadowing the results and the significance of the paper in the Introduction. This section should focus on background and rationale rather than anticipated outcomes.

Reviewer 2 made a similar request, prompting us to revise the end of the introduction. As such, this paragraph was greatly simplified and shortened. It still briefly references the study's primary outcomes in a single sentence, as we believe this enhances the manuscript's clarity and is a common organizational structure in neuroscience/biology journals (see line 71 and/or the reply to Reviewer 2's first suggestion).

Lines 100–101: Although the protocol is relatively simple, I suggest providing a more complete description here so that the Methods section is fully self-contained.

We agree with the reviewer and have included a brief summary of the protocol for Experiment 1 (panel E), similar to the pre-existing description of Experiment 2 in Panel G. This allows readers to understand the fundamentals of the protocol without referring to the Methods section.

> *Figure 1 (line 128)*
>
> *(E) Protocol for Experiment 1.* ***Participants performed two blocks, both consisting of a preferred (panel C, 5 min eyes open and 5 min eyes closed) and target (panel D, 6 targets for 2min each) trial set with a ~10 min sitting break between each block. The starting set (i.e. preferred vs. target) was random in the first block and reversed in the second.***

Line 200: I recommend toning down this statement. As the authors themselves note, and as is visible in Figure 3, the model only partially captures the EMG activity. The claim should therefore be expressed more cautiously.

We agree with the reviewer that the section heading should be formulated more cautiously. We have revised the heading:

> *Musculoskeletal model simulations predict the* ***trend of*** *metabolic cost* ***across*** *postures (line 189)*

Additional requirements

Have the authors made all data and (if applicable) computational code underlying the findings in their manuscript fully available?
The PLOS Data policy requires authors to make all data and code underlying the findings described in their manuscript fully available without restriction, with rare exception (please refer to the Data Availability Statement in the manuscript PDF file). The data and code should be provided as part of the manuscript or its supporting information, or deposited to a public repository. For example, in addition to summary statistics, the data points behind means, medians and variance measures should be available. If there are restrictions on publicly sharing data or code —e.g. participant privacy or use of data from a third party—those must be specified.

Reviewer #1: **No:** While I believe the authors tried to provide a link to the code and data, I wasn't able to access the DOI link they provided. The text didn't contain any hyperlink. It should be an easily fixable issue.

Reviewer #2: Yes

Reviewer #3: Yes

The doi link to the data repository has been provided in the Data availability statement (https://doi.org/10.34894/RAWEHM).

PLOS authors have the option to publish the peer review history of their article (what does this mean?). If published, this will include your full peer review and any attached files.

If you choose “no”, your identity will remain anonymous but your review may still be made public.

Do you want your identity to be public for this peer review? For information about this choice, including consent withdrawal, please see our Privacy Policy.

Reviewer #1: No

Reviewer #2: No

Reviewer #3: No

[NOTE: If reviewer comments were submitted as an attachment file, they will be attached to this email and accessible via the submission site. Please log into your account, locate the manuscript record, and check for the action link "View Attachments". If this link does not appear, there are no attachment files.]

Figure resubmission:

While revising your submission, we strongly recommend that you use PLOS's NAAS tool (https://ngplosjournals.pagemajik.ai/artanalysis) to test your figure files. NAAS can convert your figure files to the TIFF file type and meet basic requirements (such as print size, resolution), or provide you with a report on issues that do not meet our requirements and that NAAS cannot fix.

After uploading your figures to PLOS's NAAS tool - https://ngplosjournals.pagemajik.ai/artanalysis, NAAS will process the files provided and display the results in the "Uploaded Files" section of the page as the processing is complete. If the uploaded figures meet our requirements (or NAAS is able to fix the files to meet our requirements), the figure will be marked as "fixed" above. If NAAS is unable to fix the files, a red "failed" label will appear above. When NAAS has confirmed that the figure files meet our requirements, please download the file via the download option, and include these NAAS processed figure files when submitting your revised manuscript.

Reproducibility:

To enhance the reproducibility of your results, we recommend that authors of applicable studies deposit laboratory protocols in protocols.io, where a protocol can be assigned its own identifier (DOI) such that it can be cited independently in the future. Additionally, PLOS ONE offers an option to publish peer-reviewed clinical study protocols. Read more information on sharing protocols at https://plos.org/protocols?utm_medium=editorial-email&utm_source=authorletters&utm_campaign=protocols

The current study is not applicable to Protocols.

References

Casadio, M., Morasso, P. G., & Sanguineti, V. (2005). Direct measurement of ankle stiffness during quiet standing: implications for control modelling and clinical application. *Gait Posture*, *21*(4), 410-424. https://doi.org/10.1016/j.gaitpost.2004.05.005

Collins, J. J., & De Luca, C. J. (1993). Open-loop and closed-loop control of posture: A random-walk analysis of center-of-pressure trajectories. *Experimental Brain Research*, *95*(2), 308-318. https://doi.org/10.1007/bf00229788

Crouter, S. E., Lamunion, S. R., Hibbing, P. R., Kaplan, A. S., & Bassett, D. R. (2019). Accuracy of the Cosmed K5 portable calorimeter. *PLoS One*, *14*(12), e0226290. https://doi.org/10.1371/journal.pone.0226290

Guidetti, L., Meucci, M., Bolletta, F., Emerenziani, G. P., Gallotta, M. C., & Baldari, C. (2018). Validity, reliability and minimum detectable change of COSMED K5 portable gas exchange system in breath-by-breath mode. *PLoS One*, *13*(12), e0209925. https://doi.org/10.1371/journal.pone.0209925

Horak, F. B. (2006). Postural orientation and equilibrium: what do we need to know about neural control of balance to prevent falls? *Age Ageing*, *35 Suppl 2*, ii7-ii11. https://doi.org/10.1093/ageing/afl077

Houdijk, H., Brown, S. E., & van Dieen, J. H. (2015). Relation between postural sway magnitude and metabolic energy cost during upright standing on a compliant surface. *J Appl Physiol (1985)*, *119*(6), 696-703. https://doi.org/10.1152/japplphysiol.00907.2014

Kiemel, T., Zhang, Y., & Jeka, J. J. (2011). Identification of neural feedback for upright stance in humans: stabilization rather than sway minimization. *J Neurosci*, *31*(42), 15144-15153. https://doi.org/10.1523/JNEUROSCI.1013-11.2011

Le Sant, G., Nordez, A., Andrade, R., Hug, F., Freitas, S., & Gross, R. (2017). Stiffness mapping of lower leg muscles during passive dorsiflexion. *J Anat*, *230*(5), 639-650. https://doi.org/10.1111/joa.12589

Leeuwis, M., van Aerts, N., Seth, A., & Forbes, P. A. (2026). *Data and code for "The energetic cost of human standing balance and gait initiation over a range of natural postures"*. https://doi.org/10.34894/RAWEHM

Loeb, G. E. (2012). Optimal isn't good enough. *Biological Cybernetics*, *106*(11-12), 757-765. https://doi.org/10.1007/s00422-012-0514-6

Manini, T. M. (2010). Energy expenditure and aging. *Ageing Research Reviews*, *9*(1), 1-11. https://doi.org/https://doi.org/10.1016/j.arr.2009.08.002

Morasso, P. G., & Sanguineti, V. (2002). Ankle muscle stiffness alone cannot stabilize balance during quiet standing. *J Neurophysiol*, *88*(4), 2157-2162. https://doi.org/10.1152/jn.2002.88.4.2157

Morasso, P. G., & Schieppati, M. (1999). Can muscle stiffness alone stabilize upright standing? *J Neurophysiol*, *82*(3), 1622-1626. https://doi.org/10.1152/jn.1999.82.3.1622

Moseley, A. M., Crosbie, J., & Adams, R. (2001). Normative data for passive ankle plantarflexion--dorsiflexion flexibility. *Clin Biomech (Bristol, Avon)*, *16*(6), 514-521. https://doi.org/10.1016/s0268-0033(01)00030-4

Rajagopal, A., Dembia, C. L., DeMers, M. S., Delp, D. D., Hicks, J. L., & Delp, S. L. (2016). Full-Body Musculoskeletal Model for Muscle-Driven Simulation of Human Gait. *IEEE Trans Biomed Eng*, *63*(10), 2068-2079. https://doi.org/10.1109/TBME.2016.2586891

Rodman, P. S., & McHenry, H. M. (1980). Bioenergetics and the origin of hominid bipedalism. *Am J Phys Anthropol*, *52*(1), 103-106. https://doi.org/10.1002/ajpa.1330520113

Seth, A., Hicks, J. L., Uchida, T. K., Habib, A., Dembia, C. L., Dunne, J. J., Ong, C. F., DeMers, M. S., Rajagopal, A., Millard, M., Hamner, S. R., Arnold, E. M., Yong, J. R., Lakshmikanth, S. K., Sherman, M. A., Ku, J. P., & Delp, S. L. (2018). OpenSim: Simulating musculoskeletal dynamics and neuromuscular control to study human and animal movement. *PLoS Comput Biol*, *14*(7), e1006223. https://doi.org/10.1371/journal.pcbi.1006223

Shiozawa, K., Lee, J., Russo, M., Sternad, D., & Hogan, N. (2021). Frequency-dependent force direction elucidates neural control of balance. *Journal of NeuroEngineering and Rehabilitation*, *18*(1). https://doi.org/10.1186/s12984-021-00907-2

Sun, X. G., Hansen, J. E., Garatachea, N., Storer, T. W., & Wasserman, K. (2002). Ventilatory efficiency during exercise in healthy subjects. *Am J Respir Crit Care Med*, *166*(11), 1443-1448. https://doi.org/10.1164/rccm.2202033

Tessari, F., Hermus, J., Sugimoto-Dimitrova, R., & Hogan, N. (2024). Brownian processes in human motor control support descending neural velocity commands. *Scientific Reports*, *14*(1). https://doi.org/10.1038/s41598-024-58380-5

Tessari, F., West, A. M., & Hogan, N. (2025). Explaining human motor coordination via the synergy expansion hypothesis. *Proceedings of the National Academy of Sciences*, *122*(13). https://doi.org/10.1073/pnas.2501705122

Vlutters, M., Boonstra, T. A., Schouten, A. C., & van der Kooij, H. (2015). Direct measurement of the intrinsic ankle stiffness during standing. *J Biomech*, *48*(7), 1258-1263. https://doi.org/10.1016/j.jbiomech.2015.03.004
