# Supplementary material for: The energetic cost of human standing balance and gait initiation over a range of natural postures
Source: PLoS Comput Biol. 2026 Jul 7;22(7):e1013522. doi: 10.1371/journal.pcbi.1013522 (PMC13340847; doi:10.1371/journal.pcbi.1013522)
Supplement: Peer review history

# Peer review: Decision Letter 0

**DOI:** 10.1371/journal.pcbi.1013522.r001

**Authors:** Thomas Serre, Silvia Buscaglione

19 Feb 2026

PCOMPBIOL-D-25-01863

The energetic cost of human standing balance and gait initiation over a range of natural postures

PLOS Computational Biology

Dear Dr. Forbes,

Thank you for submitting your manuscript to PLOS Computational Biology. After careful consideration, we feel that it has merit but does not fully meet PLOS Computational Biology's publication criteria as it currently stands. Therefore, we invite you to submit a revised version of the manuscript that addresses the points raised during the review process.

Please submit your revised manuscript by Apr 21 2026 11:59PM. If you will need more time than this to complete your revisions, please reply to this message or contact the journal office at ploscompbiol@plos.org. When you're ready to submit your revision, log on to https://www.editorialmanager.com/pcompbiol/ and select the 'Submissions Needing Revision' folder to locate your manuscript file.

Please include the following items when submitting your revised manuscript:

* A letter that responds to each point raised by the editor and reviewer(s). You should upload this letter as a separate file labeled 'Response to Reviewers'. This file does not need to include responses to formatting updates and technical items listed in the 'Journal Requirements' section below.

* A marked-up copy of your manuscript that highlights changes made to the original version. You should upload this as a separate file labeled 'Revised Manuscript with Track Changes'.

* An unmarked version of your revised paper without tracked changes. You should upload this as a separate file labeled 'Manuscript'.

If you would like to make changes to your financial disclosure, competing interests statement, or data availability statement, please make these updates within the submission form at the time of resubmission. Guidelines for resubmitting your figure files are available below the reviewer comments at the end of this letter

We look forward to receiving your revised manuscript.

Kind regards,

Silvia Buscaglione, Ph.D.

Guest Editor

PLOS Computational Biology

Thomas Serre

Section Editor

PLOS Computational Biology

**Additional Editor Comments:**

This study is of high quality and relevant to biomechanical field. However, some issues must be addressed before further consideration of the manuscript, particularly with respect to the completeness of the literature review, manuscript structure, and the consistency of the musculoskeletal model. Additionally, the authors are required to update the provided data/code DOI, which is currently not accessible.

We encourage the authors to carefully address all the comments from reviewrs and to provide a detailed, point-by-point response.

**Journal Requirements:**

If the reviewer comments include a recommendation to cite specific previously published works, please review and evaluate these publications to determine whether they are relevant and should be cited. There is no requirement to cite these works unless the editor has indicated otherwise.

1) Please ensure that the CRediT author contributions listed for every co-author are completed accurately and in full.

At this stage, the following Authors/Authors require contributions: Matto Leeuwis. Please ensure that the full contributions of each author are acknowledged in the "Add/Edit/Remove Authors" section of our submission form.

The list of CRediT author contributions may be found here: https://journals.plos.org/ploscompbiol/s/authorship#loc-author-contributions

2) We ask that a manuscript source file is provided at Revision. Please upload your manuscript file as a .doc, .docx, .rtf or .tex. If you are providing a .tex file, please upload it under the item type u2018LaTeX Source Fileu2019 and leave your .pdf version as the item type u2018Manuscriptu2019.

3) Please upload all main figures as separate Figure files in .tif or .eps format. For more information about how to convert and format your figure files please see our guidelines:

https://journals.plos.org/ploscompbiol/s/figures

4) Some material included in your submission may be copyrighted. According to PLOSu2019s copyright policy, authors who use figures or other material (e.g., graphics, clipart, maps) from another author or copyright holder must demonstrate or obtain permission to publish this material under the Creative Commons Attribution 4.0 International (CC BY 4.0) License used by PLOS journals. Please closely review the details of PLOSu2019s copyright requirements here: PLOS Licenses and Copyright. If you need to request permissions from a copyright holder, you may use PLOS's Copyright Content Permission form.

Please respond directly to this email and provide any known details concerning your material's license terms and permissions required for reuse, even if you have not yet obtained copyright permissions or are unsure of your material's copyright compatibility. Once you have responded and addressed all other outstanding technical requirements, you may resubmit your manuscript within Editorial Manager.

Potential Copyright Issues:

i) Please confirm (a) that you are the photographer of 1A, or (b) provide written permission from the photographer to publish the photo(s) under our CC BY 4.0 license.

ii) Figures 1, 3, and 4A. Please confirm whether you drew the images / clip-art within the figure panels by hand. If you did not draw the images, please provide (a) a link to the source of the images or icons and their license / terms of use; or (b) written permission from the copyright holder to publish the images or icons under our CC BY 4.0 license. Alternatively, you may replace the images with open source alternatives. See these open source resources you may use to replace images / clip-art:

- https://commons.wikimedia.org

- https://openclipart.org/.

5) Please note that your Data Availability Statement is currently missing the DOI/accession number of each dataset OR a direct link to access each dataset. If your manuscript is accepted for publication, you will be asked to provide these details on a very short timeline. We therefore suggest that you provide this information now, though we will not hold up the peer review process if you are unable.

**Reviewers' comments:**

Reviewer's Responses to Questions

**Comments to the Authors:**

**Please note here if the review is uploaded as an attachment.**

Reviewer #1: Dear Authors,

Your work presents a study on the energy consumption mechanisms during unperturbed quite stance with respect to the whole-body orientation.

The work is well written, novel, and of high interest to the biomechanics and human neuromotor control community.

I honestly appreciated the rigor and quality with which your wrote your manuscript.

You will be able to find all my comments, questions, and recommendations in the attached PDF file in the form of highlighted portions of text with annexed comments.

As general comments, I think your work would benefit from 2 major improvements:

- Literature Review: I provided multiple references that I believe are highly relevant to the presented work and would enrich the introduction and discussion sections of your manuscript.

- Figure presentation: In the version I was given access, most figures had a very low resolution which reduced the overall quality of the figure and possibility to appreciate the details of the presented data. I recommend switching from images to vector graphics, if possible.

Please address all of my comments here and in the attached PDF.

I very much look forward to reading the final version of this manuscript.

Kind regards,

The Reviewer

Reviewer #2: The present manuscript investigates the energetic cost as a function of body inclination angle and in relation to gait initiation, combining experimental measurements (incl. calorimetry) with musculoskeletal simulations. The study aims to critically evaluate the commonly applied assumption of symmetric energetic components in anterior and posterior lean directions.

The topic is highly relevant and well aligned with the scope of PLOS Computational Biology. The chosen methodological approach is appropriate for addressing the research question, and both the rationale and the overall methodology are clearly and logically presented.

While the manuscript is generally well written and readable, its structure is unfortunately highly redundant in several parts. For example, lines 73–86 as well as Figure 1 do not belong into the Introduction. Moreover, the boundaries between the Introduction, Materials & Methods, and Results sections are often blurred, which affects clarity. A more streamlined organization following conventional manuscript structure is strongly recommended.

With respect to measurement techniques, simulations, and data analysis, the study has been conducted according to current standards and best-practice recommendations.

A major point of concern relates to the employed musculoskeletal model. Notably, the model does not include trunk musculature (e.g., m. erector spinae) or muscles of the upper extremities. This omission raises questions regarding the completeness of the energetic analysis.

Furthermore, there are inconsistencies between measured and simulated muscle activities. The m. erector spinae is not simulated at all, while gluteal muscles are included in the simulation but neither shown in Figure 3 nor assessed via EMG measurements. Given the known relevance of these muscle groups for posture and locomotion, it is likely that they substantially contribute to overall energy expenditure.

Despite these limitations, the physiological interpretation of the results and the derived conclusions are well reasoned and plausible.

Another point requiring clarification is that the accuracies of the experimental measurement systems are not reported, whereas simulation accuracies are described. Including this information would strengthen the methodological transparency.

The data sharing plan is exemplary, clearly described, and fully in line with open science principles.

In summary, while the manuscript requires revision, particularly regarding model completeness, structural clarity, and consistency between experimental and simulation data, the study shows strong potential. Provided that the issues outlined above are adequately addressed, I believe the manuscript would be suitable for publication in PLOS Computational Biology.

Reviewer #3: Please find attached my comments.

**********

**Have the authors made all data and (if applicable) computational code underlying the findings in their manuscript fully available?**

The PLOS Data policy requires authors to make all data and code underlying the findings described in their manuscript fully available without restriction, with rare exception (please refer to the Data Availability Statement in the manuscript PDF file). The data and code should be provided as part of the manuscript or its supporting information, or deposited to a public repository. For example, in addition to summary statistics, the data points behind means, medians and variance measures should be available. If there are restrictions on publicly sharing data or code —e.g. participant privacy or use of data from a third party—those must be specified.

Reviewer #1: **No:**  While I believe the authors tried to provide a link to the code and data, I wasn't able to access the DOI link they provided. The text didn't contain any hyperlink. It should be an easily fixable issue.

Reviewer #2: Yes

Reviewer #3: Yes

**********

PLOS authors have the option to publish the peer review history of their article (what does this mean?). If published, this will include your full peer review and any attached files.

If you choose “no”, your identity will remain anonymous but your review may still be made public.

**Do you want your identity to be public for this peer review?**  For information about this choice, including consent withdrawal, please see our Privacy Policy.

Reviewer #1: No

Reviewer #2: No

Reviewer #3: No

[NOTE: If reviewer comments were submitted as an attachment file, they will be attached to this email and accessible via the submission site. Please log into your account, locate the manuscript record, and check for the action link "View Attachments". If this link does not appear, there are no attachment files.]

**Figure resubmission:**

While revising your submission, we strongly recommend that you use PLOS’s NAAS tool (https://ngplosjournals.pagemajik.ai/artanalysis) to test your figure files. NAAS can convert your figure files to the TIFF file type and meet basic requirements (such as print size, resolution), or provide you with a report on issues that do not meet our requirements and that NAAS cannot fix.

After uploading your figures to PLOS’s NAAS tool - https://ngplosjournals.pagemajik.ai/artanalysis, NAAS will process the files provided and display the results in the "Uploaded Files" section of the page as the processing is complete. If the uploaded figures meet our requirements (or NAAS is able to fix the files to meet our requirements), the figure will be marked as "fixed" above. If NAAS is unable to fix the files, a red "failed" label will appear above. When NAAS has confirmed that the figure files meet our requirements, please download the file via the download option, and include these NAAS processed figure files when submitting your revised manuscript.

**Reproducibility:**

To enhance the reproducibility of your results, we recommend that authors of applicable studies deposit laboratory protocols in protocols.io, where a protocol can be assigned its own identifier (DOI) such that it can be cited independently in the future. Additionally, PLOS ONE offers an option to publish peer-reviewed clinical study protocols. Read more information on sharing protocols at https://plos.org/protocols?utm_medium=editorial-email&utm_source=authorletters&utm_campaign=protocols

Attachment Submitted filename: PCOMPBIOL-D-25-01863_reviewer.pdf

Attachment Submitted filename: postural_control_2026.pdf

# Author comment: Author response to Decision Letter 1

**DOI:** 10.1371/journal.pcbi.1013522.r002

22 Apr 2026

Attachment Submitted filename: R1 - Rebuttal.pdf

# Peer review: Decision Letter 1

**DOI:** 10.1371/journal.pcbi.1013522.r003

**Authors:** Thomas Serre, Silvia Buscaglione, Thomas Serre, Silvia Buscaglione

8 Jun 2026

Dear Dr. Forbes,

We are pleased to inform you that your manuscript 'The energetic cost of human standing balance and gait initiation over a range of natural postures' has been provisionally accepted for publication in PLOS Computational Biology.

Before your manuscript can be formally accepted you will need to complete some formatting changes, which you will receive in a follow up email. A member of our team will be in touch with a set of requests.

Please note that your manuscript will not be scheduled for publication until you have made the required changes, so a swift response is appreciated.

IMPORTANT: The editorial review process is now complete. PLOS will only permit corrections to spelling, formatting or significant scientific errors from this point onwards. Requests for major changes, or any which affect the scientific understanding of your work, will cause delays to the publication date of your manuscript.

Should you, your institution's press office or the journal office choose to press release your paper, you will automatically be opted out of early publication. We ask that you notify us now if you or your institution is planning to press release the article. All press must be co-ordinated with PLOS.

Thank you again for supporting Open Access publishing; we are looking forward to publishing your work in PLOS Computational Biology.

Best regards,

Silvia Buscaglione, Ph.D.

Guest Editor

PLOS Computational Biology

Thomas Serre

Section Editor

PLOS Computational Biology

***********************************************************

Reviewer's Responses to Questions

**Comments to the Authors:**

**Please note here if the review is uploaded as an attachment.**

Reviewer #2: Compliments to the authors for the highly detailed and thorough revision of the manuscript. All comments have been appropriately addressed and incorporated into the revised version. In my opinion, the manuscript has significantly improved in both quality and scientific rigor; it is now clear, well structured, and the results are presented in a very coherent and comprehensible manner. Therefore, I recommend acceptance of the present manuscript for publication in PLOS Computational Biology.

Reviewer #3: The authors have addressed all my comments and the manuscript is now much improved.

Reviewer #4: The authors have sufficiently addressed all comments. The restructuring helps the flow of the article significantly.

**********

**Have the authors made all data and (if applicable) computational code underlying the findings in their manuscript fully available?**

The PLOS Data policy requires authors to make all data and code underlying the findings described in their manuscript fully available without restriction, with rare exception (please refer to the Data Availability Statement in the manuscript PDF file). The data and code should be provided as part of the manuscript or its supporting information, or deposited to a public repository. For example, in addition to summary statistics, the data points behind means, medians and variance measures should be available. If there are restrictions on publicly sharing data or code —e.g. participant privacy or use of data from a third party—those must be specified.

Reviewer #2: Yes

Reviewer #3: Yes

Reviewer #4: Yes

**********

PLOS authors have the option to publish the peer review history of their article (what does this mean?). If published, this will include your full peer review and any attached files.

If you choose “no”, your identity will remain anonymous but your review may still be made public.

**Do you want your identity to be public for this peer review?**  For information about this choice, including consent withdrawal, please see our Privacy Policy.

Reviewer #2: **Yes:**  Jörg Miehling

Reviewer #3: No

Reviewer #4: No

# Editor report: Acceptance letter

**DOI:** 10.1371/journal.pcbi.1013522.r004

**Authors:** Thomas Serre, Silvia Buscaglione, Thomas Serre, Silvia Buscaglione

PCOMPBIOL-D-25-01863R1

The energetic cost of human standing balance and gait initiation over a range of natural postures

Dear Dr Forbes,

I am pleased to inform you that your manuscript has been formally accepted for publication in PLOS Computational Biology. Your manuscript is now with our production department and you will be notified of the publication date in due course.

The corresponding author will soon be receiving a typeset proof for review, to ensure errors have not been introduced during production. Please review the PDF proof of your manuscript carefully, as this is the last chance to correct any errors. Please note that major changes, or those which affect the scientific understanding of the work, will likely cause delays to the publication date of your manuscript.

Soon after your final files are uploaded, unless you have opted out, the early version of your manuscript will be published online. The date of the early version will be your article's publication date. The final article will be published to the same URL, and all versions of the paper will be accessible to readers.

For Research, Software, and Methods articles, you will receive an invoice from PLOS for your publication fee after your manuscript has reached the completed accept phase. If you receive an email requesting payment before acceptance or for any other service, this may be a phishing scheme. Learn how to identify phishing emails and protect your accounts at https://explore.plos.org/phishing.

Thank you again for supporting PLOS Computational Biology and open-access publishing. We are looking forward to publishing your work!

With kind regards,

Sharmila Kamatchi

PLOS Computational Biology | Carlyle House, Carlyle Road, Cambridge CB4 3DN | United Kingdom ploscompbiol@plos.org | Phone +44 (0) 1223-442824 | ploscompbiol.org | @PLOSCompBiol
